# Biocatalytic and Chemo-Enzymatic Synthesis of Quinolines and 2-Quinolones by Monoamine Oxidase (MAO-N) and Horseradish Peroxidase (HRP) Biocatalysts

**DOI:** 10.1021/acscatal.2c05902

**Published:** 2023-02-22

**Authors:** Haoyue Xiang, Salvatore Ferla, Carmine Varricchio, Andrea Brancale, Nicola L. Brown, Gary W. Black, Nicholas J. Turner, Daniele Castagnolo

**Affiliations:** † Department of Chemistry, University College London, 20 Gordon Street, London WC1H 0AJ, U.K.; ‡ Medical School, Faculty of Medicine, Health and Life Science, Swansea University, Swansea SA2 8PP, U.K.; § School of Pharmacy and Pharmaceutical Sciences, Cardiff University, Cardiff CF10 3NB, U.K.; ∥ Department of Applied Sciences, Northumbria University, Newcastle upon Tyne NE1 8ST, U.K.; ⊥ Department of Chemistry, University of Manchester, Manchester Institute of Biotechnology, 131 Princess Street, Manchester M1 7DN, U.K.; ∇ University of Chemistry and Technology, Prague, 166 28 Prague 6, Czech Republic

**Keywords:** biocatalysis, monoamine oxidase, horseradish peroxidase, quinoline, quinolone

## Abstract

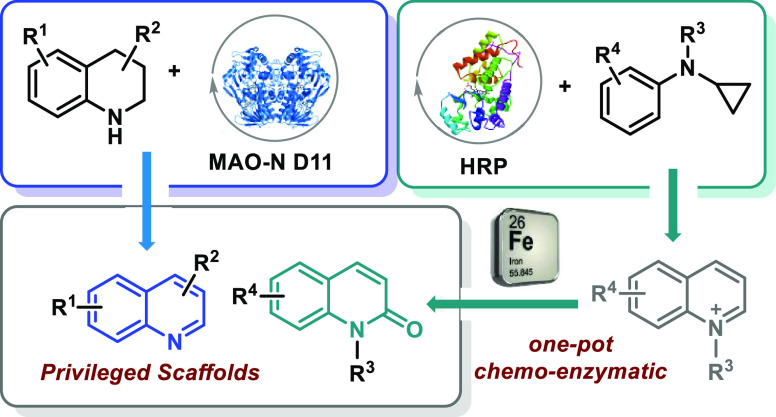

The oxidative aromatization of aliphatic *N*-heterocycles is a fundamental organic transformation for the preparation of a diverse array of heteroaromatic compounds. Despite many attempts to improve the efficiency and practicality of this transformation, most synthetic methodologies still require toxic and expensive reagents as well as harsh conditions. Herein, we describe two enzymatic strategies for the oxidation of 1,2,3,4-tetrahydroquinolines (THQs) and *N*-cyclopropyl-*N*-alkylanilines into quinolines and 2-quinolones, respectively. Whole cells and purified monoamine oxidase (MAO-N) enzymes were used to effectively catalyze the biotransformation of THQs into the corresponding aromatic quinoline derivatives, while *N*-cyclopropyl-*N*-alkylanilines were converted into 2-quinolone compounds through a horseradish peroxidase (HRP)-catalyzed annulation/aromatization reaction followed by Fe-mediated oxidation.

## Introduction

Oxidation is unarguably a fundamental transformation in synthetic chemistry and chemical industry, especially in accessing ubiquitous (hetero)aromatic compounds.^1^ Despite endless attempts to improve the efficiency and practicality of this transformation, most of the traditional oxidation reactions heavily rely on transition-metal-catalyzed or promoted strategies or on the use of toxic reagents and harsh reaction conditions.^2^ From a sustainability point of view, the development of chemical processes that extend beyond the traditional oxidations is still highly desirable and remains a long-standing challenge in synthetic chemistry. The use of enzymes as catalysts in oxidations is of great appeal because of their mild, efficient, benign, and highly selective nature.^3^ In fact, oxidases can catalyze a multitude of oxidative transformations at ambient temperature and pressure and, in some cases, without the need for any external cofactor additive or recycling system. For such reasons, oxidizing biocatalysts have been widely employed in the last decade in asymmetric reactions,^4^ like the deracemization of chiral amines by monoamine oxidases (MAO-N)^5^ or the enantioselective synthesis of alcohols by glucose oxidase (GOase M3–5)^6^ or alcohol dehydrogenases (ADH).^7^ Over the past few years, our group successfully demonstrated the possibility of employing oxidizing biocatalysts such as MAO-N and laccase also in the aromatization of aliphatic or partially saturated cyclic substrates into aromatic pyrroles, pyridines, indoles, and furans under mild reaction conditions.^8^ These transformations well demonstrated the aromatizing properties of MAO-N and laccase enzymes and encouraged us to further expand the scope of aromatic heterocycles accessible via biocatalysis.

**Figure 1:**
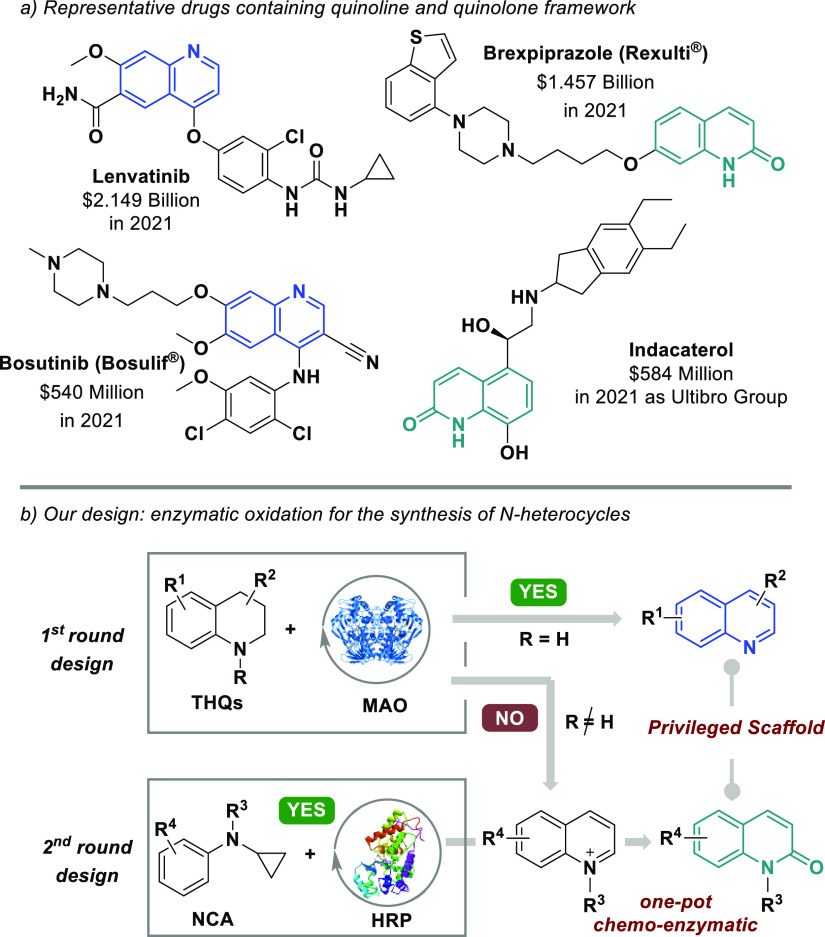
Representative bioactive molecules with the quinoline/2-quinolone motif and our design.

Quinolines and 2-quinolones are privileged scaffolds present in many drugs such as lenvatinib, brexpiprazole, bosutinib, and indacaterol (Figure 1a), and consequently, several methodologies have been reported to date for their synthesis. However, the functionalization of these *N*-heterocycles is a challenge due to the low reactivity of their π-electron-deficient skeleton.^9^ Thus, the development of practical and mild transformations that allow the synthesis of these aromatic rings from readily available starting materials is highly desirable. From a retrosynthetic standpoint, the oxidation of 1,2,3,4-tetrahydroquinolines (THQs) is the straightforward way to obtain aromatic quinolines (Figure 1b).^10^ Such an approach enables the synthesis of these heteroaromatic compounds with substitution patterns and/or functional groups that are otherwise difficult to insert via traditional aromatic functionalization reactions. As a result, several examples of oxidation of THQs into quinoline derivatives have been recently described through the use of heterogeneous polymaleimide (PMI),^11^ cobalt oxide^12^ or *o*-quinone-based^13^ catalysts, or via photocatalysis using Ru-, Ir-, or TiO_2_ photosensitizers.^14^

Nevertheless, such methods show drawbacks in terms of reaction yields and, not less important, the costs of the catalysts. Herein, we describe a new, milder, and more sustainable route to quinolines via the oxidative aromatization of THQs by MAO-N biocatalysts (Figure 1b). Although this transformation operates well for *N*-unsubstituted THQs, it was found that the presence of alkyl substituents on the THQs nitrogen posed hurdles toward the formation of quinolinium derivatives and in turn to 2-quinolone frameworks through further oxidation (Figure 1b).^15^ A different biocatalytic strategy was therefore investigated to access *N*-alkylquinolinium compounds, namely, the oxidative cyclization/aromatization of *N*-cyclopropyl-*N*-alkylanilines (NCAs) using horseradish peroxidase (HRP). Even if HRP has been the major focus of numerous structural and mechanistic investigation, due to its unusual stability in aqueous solutions,^16^ it has been rarely employed in the area of organic synthesis.^17^ Moreover, a chemo-enzymatic approach to access 2-quinolone derivatives from NCAs in a one-pot two-steps cascade, combining HRP with K_3_Fe(CN)_6_, was developed. To the best of our knowledge, this is the first example describing the direct conversion of *N*-cyclopropyl-*N*-alkylanilines into 2-quinolone scaffolds.

## Results and Discussion

The MAO-N biocatalyzed oxidation of THQs was first investigated. THQ **1a** was initially dissolved in NaPBS buffer (pH = 7.8, 1.0 M) at 37 °C in the presence of DMSO as the cosolvent and treated with three whole cell MAO-N biocatalysts (variants D5, D9, and D11)^5,8^ and a whole cell hydroxy-D-nicotine oxidase (HDNO)^8^(Table 1).

**Table 1:**
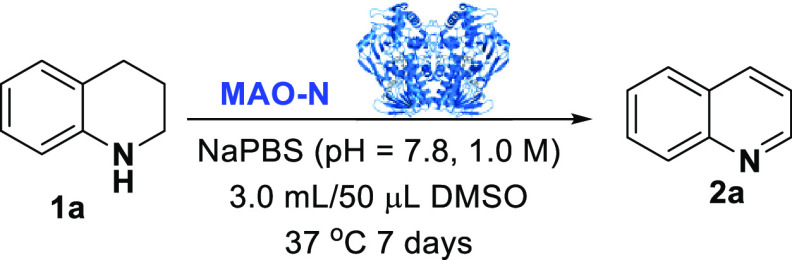
Optimization of the Reaction Conditions of the Biocatalytic Aromatization of THQs^a^

entry	MAO-N	conv.^b^
1	D5	22
2	D9	43
3	D11	56
4	HDNO	17
5^c^	D11	50
6^d^	D11	53
7^e^	D11	39
8^f^	D11	52
9	without MAO-N	0
10^g^	*E. coli* BL21(DE3) cells	0

a Reaction conditions: **1a** (0.2 mmol), freeze-dried MAO-N whole cells (190 mg), buffer (Na_2_HPO_4_/NaH_2_PO_4_, pH = 7.8, 1.0 M) (3.0 mL), DMSO (50 μL), air, 37 °C, 7 days.

b Conversion was determined by ^1^H NMR integration of the crude mixture.

c 95 mg of freeze-dried MAO-N D11 whole cells was added at the beginning of the reaction, and then, another 95 mg of freeze-dried MAO-N D11 whole cells was added at the third day of the reaction.

d 190 mg of freeze-dried MAO-N D11 whole cells was added at the beginning of the reaction, and then, another 190 mg of freeze-dried MAO-N D11 whole cells was added after 72 h.

e The reaction was stopped after 96 h.

f 2 mg of catalase was added.

g *E. coli* BL21(DE3) cells harboring no MAO-N enzymes were used.

The variant MAO-N D11 proved to be the most efficient biocatalyst affording the quinoline **2a** with 56% conversion (Table 1, *entries 1–4*). Increasing the amount of MAO-N in the reaction mixture or adding MAO-N biocatalyst in portions did not affect the reaction efficiency in a significant manner (Table 1, *entries 5–6*). Shorter reaction times provided the quinoline **2a** in lower conversion (Table 1, *entry 7*), while no improvement in the formation of the quinoline product was observed when the reaction was carried out for more than 7 days. To exclude the possibility that H_2_O_2_ produced during the biocatalytic oxidation could induce the oxidation of the THQ **1a**, catalase was added to the biotransformation mixture (Table 1, *entry 8*). Quinoline **2a** was obtained with 52% conversion in the presence of catalase, showing that H_2_O_2_ did not affect the aromatization of **1a**. Finally, two blank experiments without MAO-N or in the presence of *E. coli* BL21(DE3) cells harboring no MAO-N enzymes were carried out, confirming that the oxidation of **1a** is catalyzed by the MAO-N enzyme (Table 1, *entries 9–10*).

With the optimal conditions in hand, the reaction scope of the biocatalyzed aromatization of THQs was investigated (Table 2). A series of commercially available THQs bearing different substituents were treated with MAO-N D11 whole cells. Both the electronic nature and the substituents on the THQ backbone dramatically affected the oxidation reaction. Electron-donating substituents favored the aromatization of THQs affording the quinolines **2b–2f** with good conversions (up to 84%). On the other hand, the THQs bearing a halogen substituent (F, Cl, Br) on the aromatic ring were poorly converted into the corresponding derivatives **2g**, **2h**, and **2j**. Interestingly, the position of the substituents on the THQs also affects the reaction as shown by the 6-MeO-quinoline **2c** which was obtained with 69% conversion, while the analogue 7-MeO-quinoline **2i** resulted in only 11% conversion.

**Table 2:**
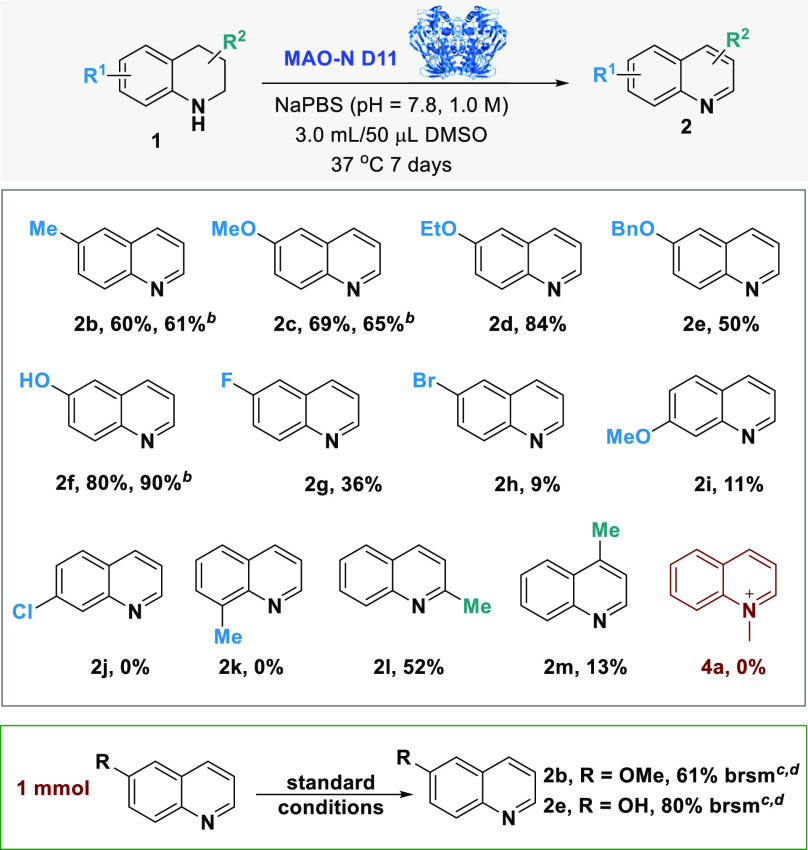
Substrate Scope of the MAO-N Biocatalytic Aromatization of THQs^a^

a Reaction conditions: **1a** (0.2 mmol), freeze-dried MAO-N D11 whole cells (190 mg), buffer (Na_2_HPO_4_/NaH_2_PO_4_, pH = 7.8, 1.0 M) (3.0 mL), DMSO (50 μL), air, 37 °C, 7 days; conversion was determined by the ^1^H NMR integration of the crude mixture.

b Purified MAO-N D11 was used.

c Isolated yield based on the recovery starting material.

d The reaction was performed in 15 mL of buffer with 250 μL DMSO.

The effect of the substituent position is clear in the series of the methyl-substituted THQs **1b** and **1k–m**. The quinolines bearing the methyl group at positions C6 (**2b**) and C2 (**2l**) were obtained with good conversions (60 and 52%, respectively) while the derivatives **2k** and **2m** bearing the methyl substituent at C8 and C4, respectively, were formed in low yields (13% for **2m**) or not formed at all (**2k**). In order to evaluate if the conversion of THQs into quinolines could be affected by the use of the whole cell biocatalyst, the substrates **1b**, **1c**, and **1f** were treated with the purified MAO-N D11 enzyme. In all cases, the quinoline derivatives **2b**, **2c**, and **2f** were formed with conversions similar to those obtained in the whole cell-catalyzed biotransformations. The scalability of this protocol was successfully demonstrated on substrates **1b** and **1e** by performing the reaction on 1 mmol scale, leading to the desired quinolines **2b** and **2e** with high yields. Finally, the MAO-N aromatization strategy was extended to the *N*-methyl derivative of **1a**. However, despite several attempts, the desired quinolinium ion **4a** was not obtained via MAO-N biocatalyzed aromatization.

In order to rationalize the results of the biocatalytic transformations and to determine if the different conversions observed were due to the diverse binding interactions of the THQs with the MAO-N active site, or to an electronic factor, or a combination of the two, a series of *in silico* studies was carried out.

**Figure 2:**
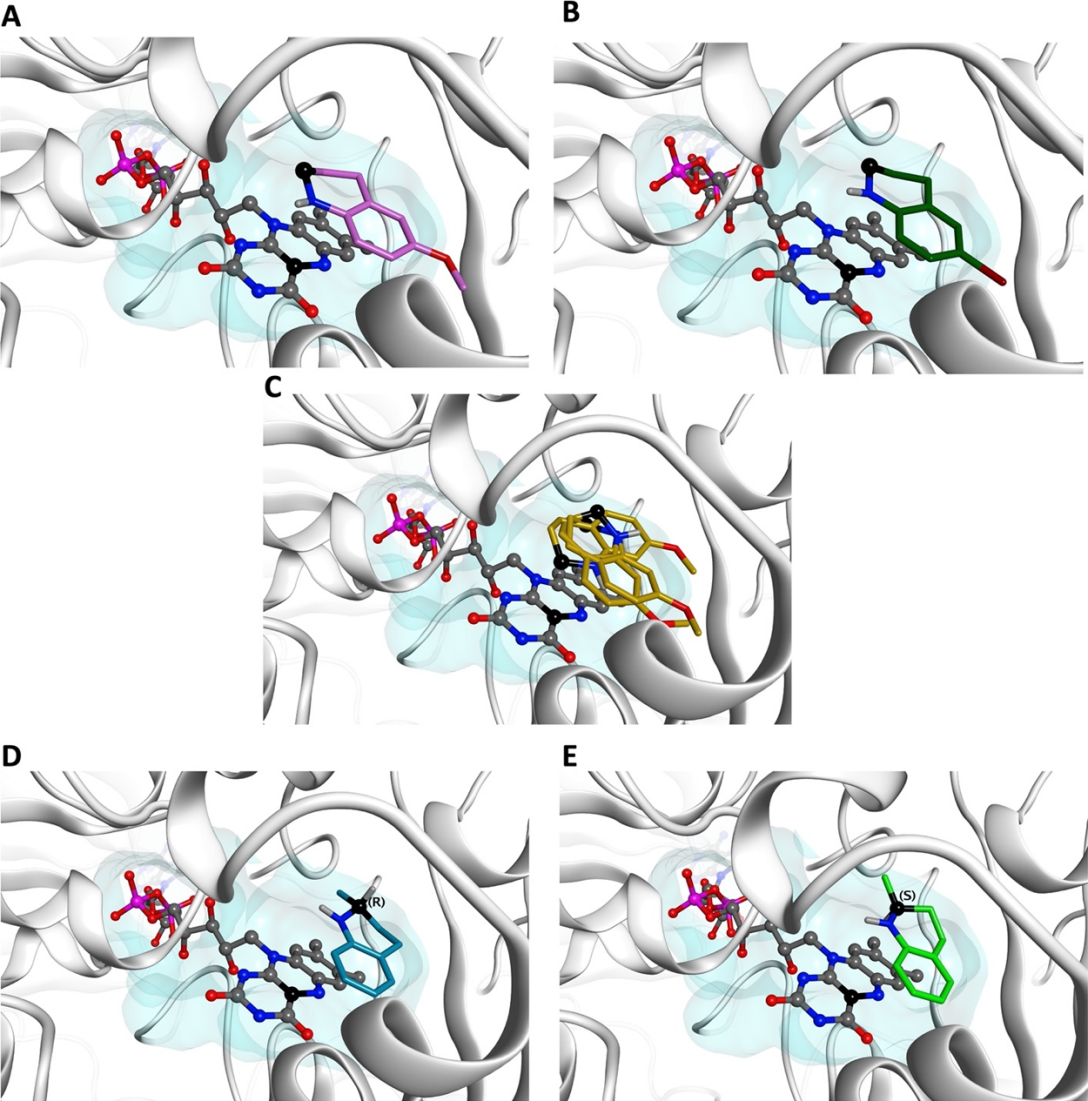
Proposed binding modes for compounds (A) **1c**, (B) **1h**,(C) **1i**, (D) **(*R*)-1l**, and (E) **(*S*)-1l** in the MAO-N D11 catalytic site (PDB ID: 3ZDN). Carbon atoms of compound **1c** are shown in purple, compound **1h** in dark green, compound **1i** in gold, compound **(*R*)-1l** in teal, and compound **(*S*)-1l** in green. The binding area of the catalytic site is represented as a transparent surface. FAD is represented as a ball-and-stick model. Nitrogen atoms of **1c**, **1h**, **1i**, **(*R*)-1l**, **(*S*)-1l** and FAD are shown in blue. The α-methylene group is shown as black ball. The nitrogen atom of **1c** and **1h** is oriented toward the FAD nitrogen and carbon atom (represented as a black atom) involved in the mechanism of the reaction, whereas the presence of 7-MeO group forces compound **1i** to adopt several possible binding modes, but none of them in line with the plausible mechanism of MAO-N biocatalytic aromatization. The methyl substituent on C2 is not impeding a correct binding to the active site for both **1l** enantiomers, but only the (*S*)-enantiomer has the α-hydride facing the FAD cofactor, while the (*R*)-enantiomer present the α-hydride pointing away.

According to the generally accepted mechanism of the MAO-N catalyzed oxidation,^18,8c^ the abstraction of a hydride from the methylene group in the α-position to the nitrogen of the THQ by the FAD cofactor represents the initial step of the catalytic cycle. All the substrates **1b–1h** bearing a substituent at C6 showed a similar and consistent binding mode to the MAO-N D11 catalytic site, with the methylene group at the α position of the THQ nitrogen correctly oriented toward the FAD cofactor (Figure 2A,B, compounds **1c** and **1h**).

**Figure 3:**
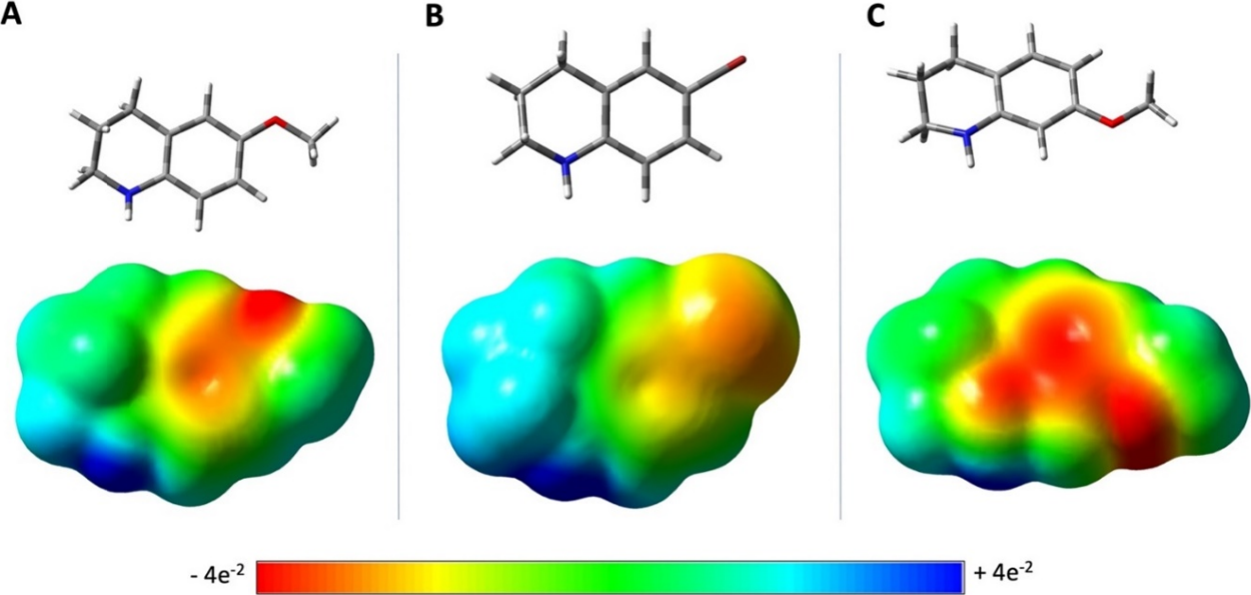
Electrostatic potential surface (EPS) for (A) **1c**, (B) **1h**, and (C) **1i**.

A closer evaluation of the electrostatic potential surface (EPS) of substrate **1c** shows a high electron density localized on its nitrogen-containing ring and on its α-methylene group which may favor the abstraction of the hydride unit by the FAD (Figure 3A).

In contrast, the presence of a Br substituent in **1h** withdraws electrons from the α-methylene group reducing its electron density (Figure 3B). As a consequence, the hydride abstraction step by the FAD cofactor and the following aromatization is facilitated for the THQ **1c** as compared to **1h**, accounting for the different conversions observed (65 versus 9%). This finding suggests the importance of the electronic factor over the binding site occupation for the substrates bearing a substituent at position C6. Differently, the lower conversions observed for the substrates **1i–1m** bearing substituents in different positions than C6 seem to be due mainly to their diverse binding interactions with the MAO-N binding site. In fact, the difference in the abundance of electrons around the nitrogen atom and the α-methylene group between **1c** and its C7 analogue **1i** is not substantial and both substrates have comparable EPS (Figure 3A,C). However, moving the methoxy group from C6 to C7 forces **1i** to modify its occupation of the MAO-N site and to adopt several possible binding modes, none of which is however in line with the plausible mechanism of the biocatalytic aromatization (Figure 2C). To accommodate the 7-MeO group in the MAO-N binding pocket, **1i** moves the α-methylene group and the nitrogen atom away from the FAD, thus potentially affecting the ability of the cofactor to abstract the α-hydride and leading in turn to quinoline **2i** in poor yields. Similarly, the presence of a substituent on the C8 (**1k**), C4 (**1m**), or directly on the THQ nitrogen (*N*-Met-**1a**) does not allow the correct orientation of these molecules (Figure S4A–D) in the MAO-N binding site. Also, in these cases, the electronic factor seems to be less relevant for the biotransformation outcomes (Figure S5).

**Figure 4:**
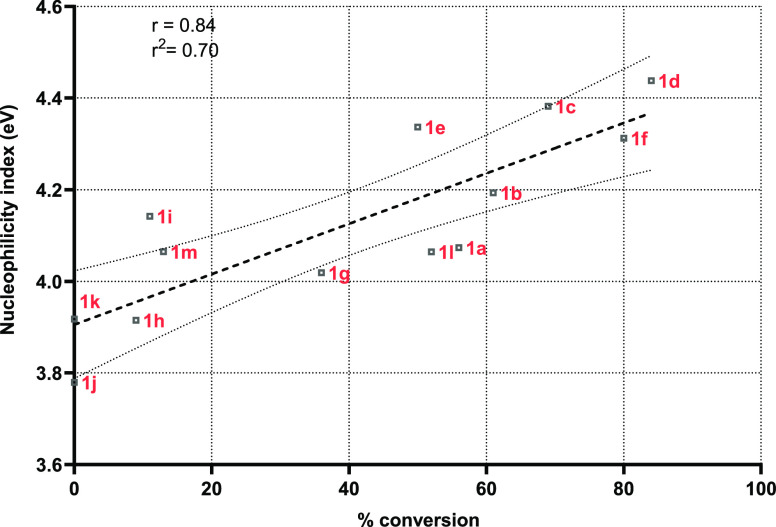
Fukui functions showing the relation between the nucleophilicity N index of THQs and the conversions of the MAO-N biocatalyzed aromatization. The nucleophilicity index N prediction was performed using Multiwfn based on the HOMO energies obtained within the Kohn–Sham scheme and defined as N = *E*_HOMO(Nu)_ – *E*_HOMO(TCE)_.

Figure 4 shows how the addition of electron-withdrawing and electron-donating groups to the THQ core influences the nucleophilicity of the α-methylene group and their consequent conversion to quinolines. Overall, there is a linear correspondence between the nucleophilicity index of the α-methylene group and the observed conversions, confirming that the electronic factor plays an important role, at different degrees, in the biotransformation outcomes (e.g., it is the main factor for compound **1c**, but it is less relevant for **1i**). All these data suggest that the MAO-N aromatization of THQs may be affected both by steric and electronic factors as well as by the binding mode of the substrates within the enzyme catalytic pocket.

Finally, the aromatization of the racemic THQ **1l** was analyzed. The docking results obtained show that the methyl substituent on C2 of **1l** does not obstruct a correct binding to the active site of MAO-N D11 and suggest that the (*S*)-enantiomer of **1l** could potentially bind the MAO-N binding pocket better than the (*R*)-enantiomer. In fact, while both enantiomers occupy the MAO-N binding site in a similar manner, only the (*S*)-enantiomer has the α-hydride facing the FAD cofactor, while the (*R*)-enantiomer presents the α-hydride pointing away (Figure 2ED). However, the unreacted THQ **1l** was recovered at the end of the biocatalytic reaction as a racemic mixture, thus suggesting that the (*R*)-enantiomer modifies its occupation of the binding site during the biotransformation.

From previous experiments, the biocatalyst MAO-N D11 was unable to convert the substrate *N*-Me-**1a** into the corresponding quinolinium product **4a** (Table 2). Inspired by former studies on the cytochrome P450 and HRP-mediated metabolism of *N*-cyclopropyl-anilines^18^ as well as on the Pd-mediated synthesis of dihydroquinolines from phenyl cyclopropyl carbamates,^19^ we decided to explore an alternative biocatalytic pathway to synthesize *N*-alkyl-quinolinium compounds **4** through an HRP-catalyzed cyclization/aromatization of *N*-cyclopropyl-*N*-alkylanilines substrates (Table 3). *N*-cyclopropyl-*N*-methylaniline **3a**(20) was initially investigated and treated with HRP and H_2_O_2_ in NaPBS buffer (pH = 5.5) in the presence of different cosolvents. Although the reaction proceeds in the absence of any organic cosolvent (Table 3, *entry 1*) affording the quinolinium **4a** in 54% NMR yield, the addition of acetone as the cosolvent proved to be beneficial, increasing the yield of **4a** to 67% (Table 3, *entry 2*). Lower yields were observed instead when other cosolvents were used (Table 3, *entries 3–*-*6*). Different oxidants than H_2_O_2_, such as the urea-hydrogen peroxide adduct (UHP), oxone, and *meta*-chloroperoxybenzoic acid (*m*CPBA) were then tested. When UHP was used, quinolinium **4a** was formed with 56% yield, comparable to the data obtained with H_2_O_2_ (Table 3, *entry 7*). On the other hand, no formation of **4a** was achieved with oxone and *m*CPBA (Table 3, *entries 8–9*). Importantly, no desired product **4a** was detected in the absence of H_2_O_2_, thus suggesting that the reaction is catalyzed by the HRP activated by the peroxide (Table 3, *entry 10*). Furthermore, when the biocatalytic cyclization/aromatization reaction was carried out without HRP and in the presence of sole H_2_O_2_, only a low amount of the desired product **4a** was observed (Table 3, *entry 11*). Lowering or increasing the amount of hydrogen peroxide led to inferior results (Table 3, *entries 12–13*).^21^

**Table 3:**
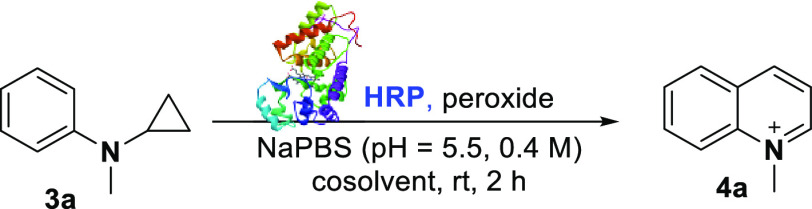
Optimization of the Reaction Conditions of the Biocatalytic Cyclization/Aromatization of *N*-Cyclopropyl-*N*-methylaniline^a^

entry	cosolvent	peroxide^c^	yield (%)^b^
1		H_2_O_2_	54
2	acetone	H_2_O_2_	67
3	THF	H_2_O_2_	50
4	DCE	H_2_O_2_	28
5	MeCN	H_2_O_2_	37
6	DMSO	H_2_O_2_	36
7	acetone	UHP	56
8	acetone	oxone	0
9	acetone	mCPBA	0
10	acetone		0
11^d^	acetone	H_2_O_2_	<10
12^e^	acetone	H_2_O_2_	33
13^f^	acetone	H_2_O_2_	52
14^g^	acetone	H_2_O_2_	58
15^h^	acetone	H_2_O_2_	61
16^i^	acetone	H_2_O_2_	60
17^i^^,^^j^	acetone	H_2_O_2_	67

a Reaction conditions: **3a** (0.08 mmol, 1 equiv), NaPBS (pH = 5.5, 0.4 M, 1 mL), cosolvent (1% v/v), peroxide (2.5 equiv), 100 μL HRP (4 mg per 1 mL NaPBS, 240 U/mg), rt, 2 h.

b NMR yield determined by ^1^H-NMR with sodium 4-methylbenzenesulfonate as the internal standard.

c Unless otherwise specified, 30% H_2_O_2_ was used.

d No HRP was added.

e 10 μL 30% H_2_O_2_ was used.

f 30 μL 30% H_2_O_2_ was used.

g The reaction was carried out under N_2_.

h 50 μL HRP was added after 0.5 h.

i 100 μL HRP was added after 0.5 h.

j 2 mL NaPBS and 20 μL acetone were used.

The nitrogen atmosphere was also unhelpful to improve the yield of **4a** (Table 3, *entry 14*), as well as increasing the loading of HRP or the volume of the buffer had no obvious effect on the yield of this biotransformation (Table 3, *entries 15–17*).

The scope and versatility of the HRP-catalyzed cyclization/aromatization strategy were then explored. A range of *N*-cyclopropyl-*N*-alkylanilines **3a–n** bearing different substituents on the phenyl ring were prepared and treated with HRP/H_2_O_2_ under the previously identified optimal reaction conditions (Table 4).

**Table 4:**
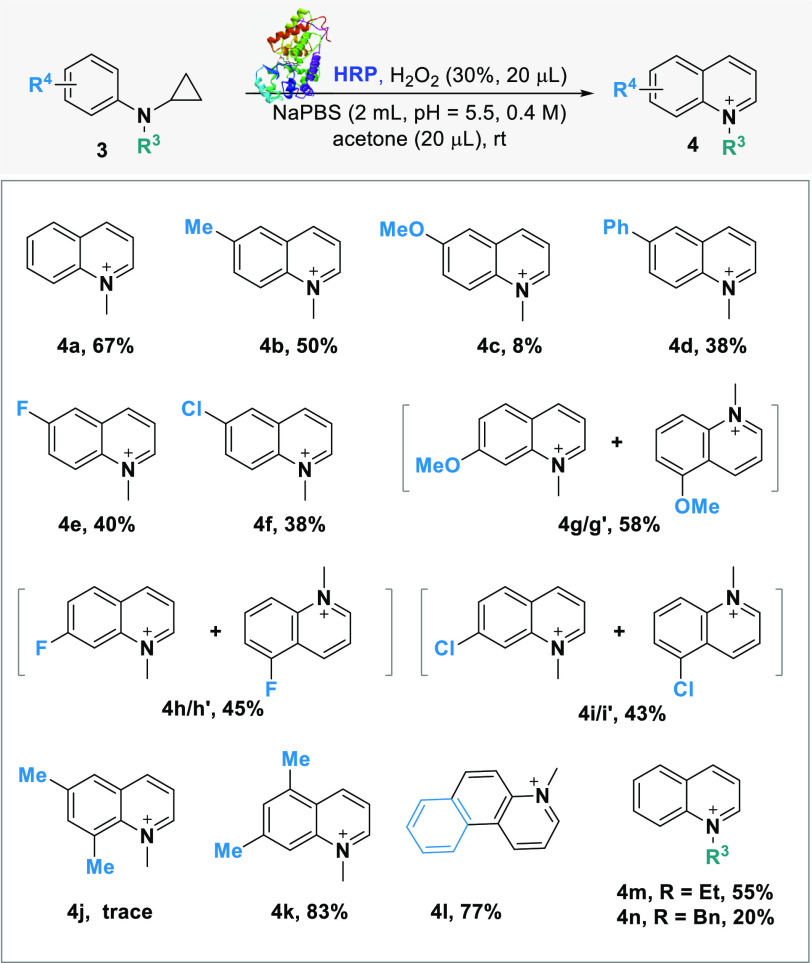
Substrate Scope of the Cyclization/Aromatization of *N*-Cyclopropyl-*N*-alkylanilines 3^a^^,^^b^

a Reaction conditions: **3** (0.08 mmol, 1 equiv), 2 mL NaPBS (pH = 5.5, 0.4 M), 20 μL acetone, 20 μL H_2_O_2_ (30%), 100 μL HRP (4 mg per 1 mL NaPBS, 240 U/mg) and then 50 μL after 0.5 h, rt.

b NMR yields determined by ^1^H NMR with sodium 4-methylbenzenesulfonate as the internal standard.

As a general trend, rather than the nature of the substituent (electron-donating groups, halogens, phenyl), the cyclization/aromatization was more affected by the position of the substituents on the phenyl ring. The *para*-Me-substituted derivative **3b** led to quinolinium **4b** with good 50% yield, while the aniline **3c** bearing a MeO-group at the *para*-position on the phenyl ring was converted to **4c** with low yield. It was found that **4c** was quite unstable even under air conditions, due to its electron-rich properties, and this may have affected the outcome of the reaction. On the other hand, aniline **3g** bearing a MeO-group at the *meta*-position was converted into an inseparable mixture of the isomers **4g/4 g′** (1:1 ratio) with good 58% yield, thus highlighting the effect of the substituents on the outcome of the reaction. Similarly, the di-substituted anilines **3j** (*ortho*-*para*-dimethyl) and **3k** (*meta*-dimethyl) showed opposite reactivity. No reaction occurred for substrate **3j**, showing that a second substituent at the *ortho*-position was detrimental for the biotransformation. Such an effect, especially if compared with substrate **3b**, may be attributed to steric hindrance. In contrast, derivative **3k** was converted into quinolinium **4k** with excellent 83% yield.

The anilines **3d**, **3e**, and **3f** bearing, respectively, a phenyl, a fluorine, and a chlorine substituent at the *para*-position of the phenyl ring were all converted into the corresponding quinolinium ions **4d–f** with good yields, while both the F- and Cl-*meta*-substituted anilines **3h** and **3i** were converted with good yields into the corresponding **4h/4h′** and **3i/3i′** as isomeric mixtures (1:1 ratio of 5- and 7-substituted quinolinium ions). Remarkably, the tricyclic product **4L** was smoothly synthesized in 77% yield. Finally, the two N-substituted anilines **3m–n**, bearing an Et or Bn group on the *N*-atom, were also investigated. Both anilines were converted into the *N*-alkyl quinolinium ions **4m** and **4n**, respectively, in good-moderate yields.^22^

**Scheme 1:**
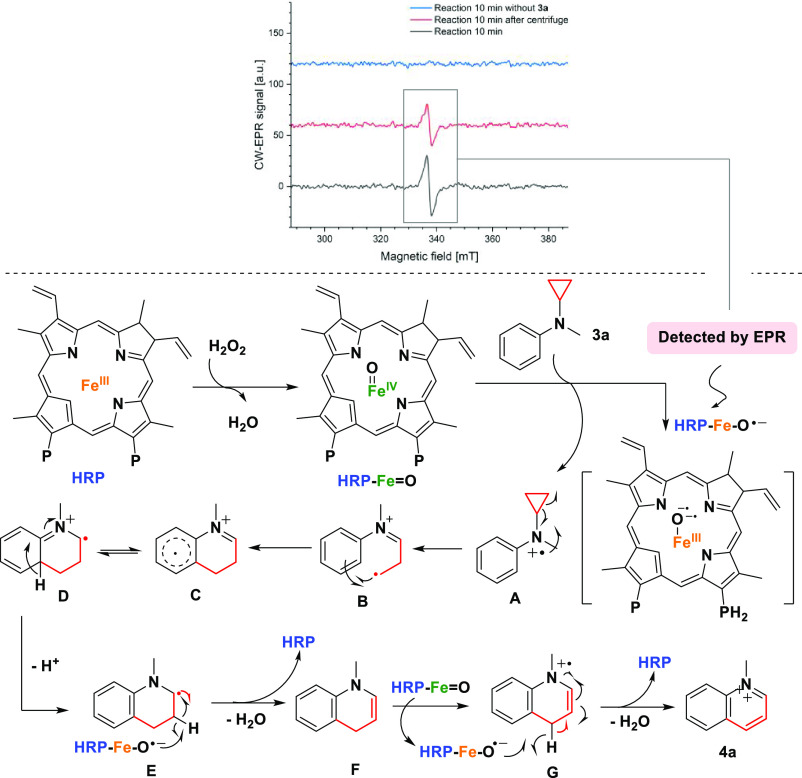
Proposed Mechanism for the HRP-Catalyzed Oxidation Process

Based on previous reports^23^ and our experiments, a proposed mechanism for the HRP biocatalyzed cyclization/aromatization of *N*-cyclopropyl-*N*-alkylanilines **3** was proposed (Scheme 1). Initially, the rapid transfer of an oxygen atom from H_2_O_2_ to the ferric heme cofactor of HRP produced a porphyrin cation-radical/oxoiron complex HRP-Fe=O and a molecule of water. The amine substrates were oxidized by HRP-Fe=O through a single electron transfer (SET) process, giving rise to an *N*-center radical **A** and HRP-Fe–O^.–^. In situ EPR experiments provided further evidence for the possible involvement of the HRP-Fe–O^. –^ radical in this transformation. The ring opening of **A** formed the radical **B** which collapsed on the aromatic ring leading, after deprotonation, to the intermediate **E**. A second one-electron oxidation of **E** forms the enamine **F**, with the regeneration of HRP. The enamine **F** is ultimately oxidized by HRP-Fe-O^.-^ into the final quinolinium ion **4a** in a similar way.

Finally, the synthetic potential of this HRP biocatalyzed transformation was explored through the development of a chemo-enzymatic sequence to convert anilines **3** into 2-quinolone compound **5** in a two-step one-pot manner (Table 5).

**Table 5:**
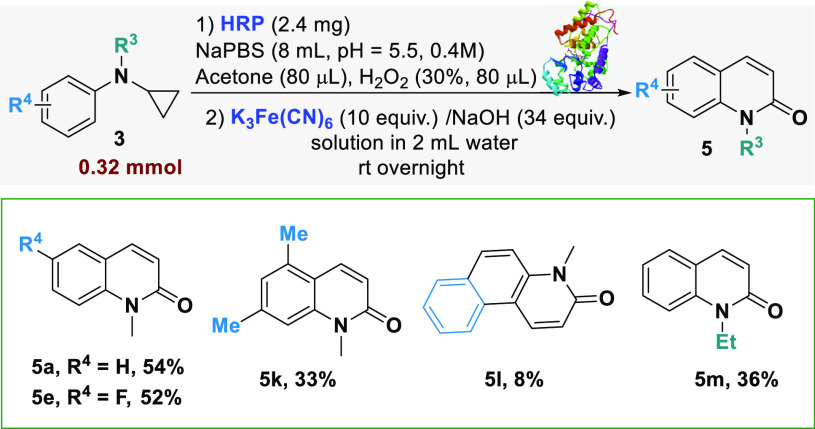
Chemo-Enzymatic Cascade for the Synthesis of 2-Quinolones **5**^a^

a Two steps one-pot cascade. K_3_Fe(CN)_6_ was added after the completion of the first biocatalytic step.

Quinolinium ions are reactive substrates which can be further carbonylated into quinolone derivates through the addition of K_3_Fe(CN)_6_.^24^ The four *N*-cyclopropyl-*N*-alkylanilines which gave the best results in the substrate scope screening were treated with HRP and H_2_O_2_ and then added with K_3_Fe(CN)_6_ and NaOH. The 2-quinolone derivates **5a**, **5e**, **5k**, and **5m** (Table 5) were all obtained with good isolated yields from the corresponding anilines in a one-pot two-step chemo-enzymatic cascade. The tricyclic product **5l** was also obtained and isolated from the chemo-enzymatic cascade, albeit with a lower yield.

## Conclusions

In conclusion, we have developed two new biocatalytic methodologies for the synthesis and construction of quinoline and 2-quinolone heterocycles using two different oxidase enzymes. A series of quinoline derivatives were obtained from 1,2,3,4-tetrahydroquinoline substrates using monoamine oxidase (MAO-N) biocatalysts in good yields. Computational studies highlighted that the MAO-N biotransformation may be affected both by steric and binding effects as well as by the electronic properties of the THQ substrates. In parallel, HPR was successfully employed in the construction of a range of quinolinium derivatives from *N*-cyclopropyl-*N*-alkylaniline substrates through a cyclization/aromatization radical cascade, highlighting the possibility to construct heteroaromatic rings from aliphatic substrates. Furthermore, a chemo-enzymatic sequence was designed and successfully developed to convert *N*-cyclopropyl-*N*-alkylanilines into 2-quinolones in a one-pot procedure. These results further confirm the key role of biocatalysis in the synthesis of a variety of organic molecules, including nonchiral heteroaromatic compounds.

## Supporting Information Available

Experimental details; procedures; and copies of spectra for new compounds (PDF)

## Abbreviations

MAO-N: monoamine oxidase
NCA: *N*-cyclopropyl-*N*-alkylaniline
HRP: horseradish peroxidase
UHP: urea-hydrogen peroxide
*m*CPBA: *meta*-chloroperoxybenzoic acid
