# Supplementary material for: Biocatalytic and Chemo-Enzymatic Synthesis of Quinolines and 2-Quinolones by Monoamine Oxidase (MAO-N) and Horseradish Peroxidase (HRP) Biocatalysts
Source: ACS Catal. 2023 Feb 22;13(5):3370–8. doi: 10.1021/acscatal.2c05902 (PMC9990064; doi:10.1021/acscatal.2c05902)

Biocatalytic and chemo-enzymatic synthesis of quinolines and 2-quinolones by monoamine oxidase (MAO-N) and horseradish peroxidase (HRP) biocatalysts

Haoyue Xiang,[1] Salvatore Ferla,[2] Carmine Varricchio,[3] Andrea Brancale,[3,†] Nicola L. Brown,[4] Gary W. Black,[4] Nicholas J. Turner,[5] Daniele Castagnolo[1,*]

[1]Department of Chemistry, University College London, 20 Gordon Street, WC1H 0AJ, London, United Kingdom. [2]Medical School, Faculty of Medicine, Health and Life Science, Swansea University, Swansea, UK SA2 8PP, United Kingdom. [3]School of Pharmacy and Pharmaceutical Sciences, Cardiff University, Cardiff CF10 3NB, United Kingdom. [4]Department of Applied Sciences, Northumbria University, Newcastle upon Tyne NE1 8ST, United Kingdom. [5]School of Chemistry, Manchester Institute of Biotechnology, University of Manchester, Manchester M1 7DN, United Kingdom.

Corresponding Author: ***d.castagnolo@ucl.ac.uk***

Supporting Information

Table of Contents

1. General Information S2
2. General Procedure for the Synthesis of N-cyclopropyl-N-methylanilines 3 .... S3
3. Preparation of the whole cell MAO-N biocatalysts S3
4. Preparation of the Pure MAO-N D11 .... S3
5. General Procedure for the biocatalyzed synthesis of quinolines 2 .... S5
6. General Procedure for the biocatalyzed synthesis of quinoliniums 4 and quinolones 5 .... S6
7. *In Situ* EPR Experiments S8
8. Computation Methods S8
9. Characterization data for the new compounds S10
10. References S16
11. Copies of NMR Spectra of new compounds S18

1. General Information

Unless noted, all solvents and commercially available reagents were purchased from Sigma Aldrich and used as without further purifications. Horseradish peroxidase (HRP) was purchased from Alfa Aesar (240 units/mg dry weight). 1,2,3,4-Tetrahydroquinolines were purchased from Fluorochem. ^{1}H and ^{13}C Nuclear Magnetic Resonance (NMR) spectra were recorded using a Bruker Ascend 400 spectrometer at 298 K. Chemical shifts (δ) are reported in ppm, referenced to tetramethylsilane. Coupling constants (J) are reported in Hertz. Splitting patterns are abbreviated as follows: singlet (s), doublet (d), triplet (t), quartet (q), multiplet (m). TLC was performed using commercially available pre-coated plated and visualized with UV light at 254 nm. Flash column chromatography was carried out using Sigma Aldrich silica gel particle size, 40-63 µm particle size 60 Å. Biocatalytic reactions (MAO) were performed by shaking the mixtures contained in 15 mL Falcon tubes using a Grant Bio™ PSU-10i Orbital Platform Shaker. A JouanB4 centrifuge with exchangeable buckets was used to centrifuge and isolate the biocatalytic products. HRMS (high-resolution mass) were measured on a Thermo Q-Exactive mass spectrometer with an EI/ESI/APCI source. The NMR data of the known compounds were consistent with the data already reported in literature.

2. General Procedure for the Synthesis of N-cyclopropyl-N-methylanilines 3

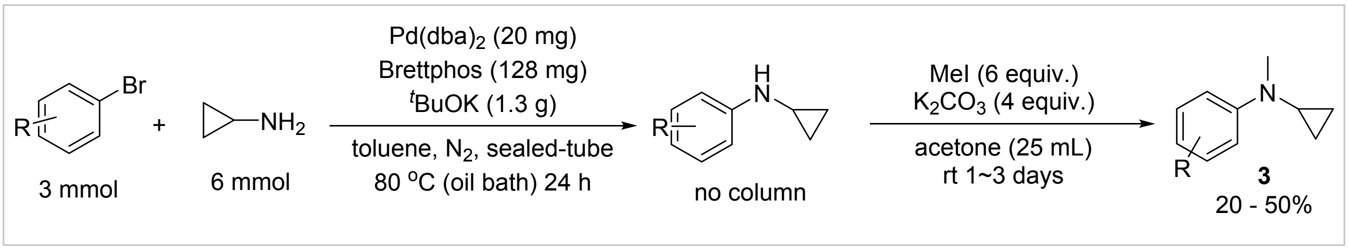

Step 1: A heavy wall cylindrical vessels (15 mL, Synthware) equipped with a Teflon coated magnetic stir bar was loaded with $Pd(dba)_2$ (20 mg), BrettPhos (128 mg) and ^{t}BuOK (1.3 g). The vessel was purged with N_2 (three times), and then toluene (5 mL), bromobenzene (3 mmol) and cyclopropylamine (6 mmol) were added. The vessel was purged with N_2 for 1 minute and screw-capped. The reaction mixture was heated at 80 °C (pre-heated oil bath) for 24 hours. Then the reaction mixture was cooled to room temperature, diluted with ethyl acetate and filtered. The filtrate was concentrated in vacuo and used for the next step without any further purification.

Step 2: To a solution of the obtained *N*-cyclopropylbenzenamine in 25 mL acetone was added K_2CO_3 (12 mmol) and MeI (18 mmol). The mixture was then stirred at room temperature for 1-3 days. Once the reaction was completed (by TLC monitoring) the solvent was removed in vacuo and the residue was purified by flash column chromatography (hexane/EtOAc = 80/1) to afford the desired products **3**.

3. Preparation of the whole cell MAO-N biocatalysts

MAO-N biocatalysts (monoamine oxidase from *Aspergillus niger*) were expressed in *E. coli* cells according to previously reported procedures.

MAO-N D5 was produced in *E. coli* BL21(DE3)

MAO-N D9 was produced in *E. coli* BL21(DE3)

MAO-N D11 was produced in *E. coli* C43(DE3)

In all cases, an overnight 10 mL starter culture of each clone was grown in LB broth + Ap (100 μg/ml) at 37 °C, 200 rpm. The starter culture was then inoculated into 1 L of Auto Induction Media Super Broth Base including trace elements (Formedium Ltd, UK) + Ap (100 μg/ml), in a 2 L baffled flask, and grown at 30 °C, 180 rpm for 2 days. Cells were then harvested by centrifugation at 4000 x g for 10 min at 4 °C. The supernatant was discarded and the cell pellet was resuspended in 10 mL of 18.2 MΩ/cm H_2O. The resuspended cells were then frozen and freeze-dried. Typically 4 g of lyophilized E. coli cells were obtained from a 600 mL culture.

4. Preparation of the Pure MAO-N D11

The plasmid DNA for MAO-N D11 was transformed into *E. coli* expression strain C43 (DE3).

Whole cell production: Expression of MAO-N D11 was carried out in 2 x 1 L Auto-induction media + Ampicillin (100μg/ml final) + 10ml MAO-N D11 C43 (DE3) starter culture and incubated at 30°C 200rpm for 48h. Culture was then centrifuged at 4000 x g for 10 min at +4°C. The pellet was resuspended in 10ml/L 18.2 MΩ H_2O, frozen at -80°C and freeze dried overnight.

Purified Cell free extract (CFE) production: Expression of MAO-N D11 was carried out in 2 x 1 L Auto-induction media + Ampicillin (100µg/ml final) + 10ml MAO-N D11 C43 (DE3) starter culture and incubated at 30°C 200rpm for 48h. Culture was then centrifuged at 4000 x g for 10 min at 4°C. The pellet was resuspended in 10ml/L 18.2 MΩ H_2O, sonicated on ice for 2 min (10sec on; 10 sec off) and centrifuged at 24 000 x g 40 min +4°C. The supernatant = CFE. Protein purification was carried out on CFE using gravity flow TALON columns. Purified proteins from TALON fractions, as judged by SDS-polyacrylamide gel, were concentrated and buffer exchanged into 20mM TRIS-HCl, pH 8.0.

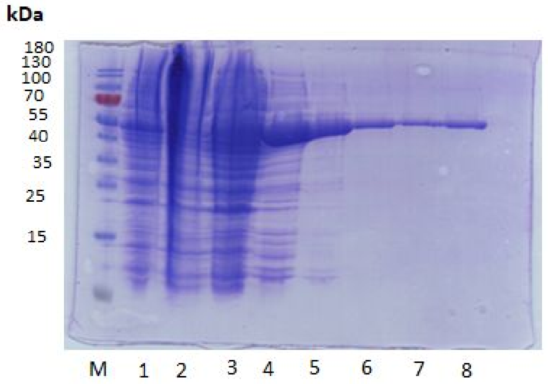

Lane M: Page Ruler Prestained Markers
1: Cell free extract (CFE; 10µl) AIM 30°C
2: TALON Flow through (15 µl)
3: TALON 10mM Imidazole (15 µl)
4: TALON 100mM Imidazole (15 µl)
5: TALON 200mM Imidazole (15 µl)
6: TALON 300mM Imidazole (15 µl)
7: TALON 400mM Imidazole (15 µl)
8: TALON 500mM Imidazole (15 µl)

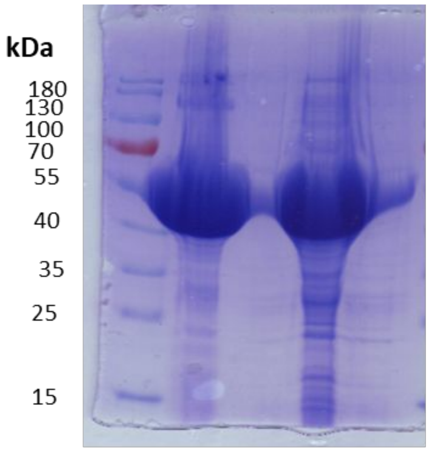

1= ~0.5ml
2= ~07ml

Buffer exchanged into 20mM Tris-HCl, pH8.0

Lane M: Page Ruler Prestained Markers
1: Concentrated 100mM TALON fraction (1:2 Dilution; 10µl)
2: Concentrated 200-500mM pooled TALON fractions (1:5 Dilution; 10 µl)

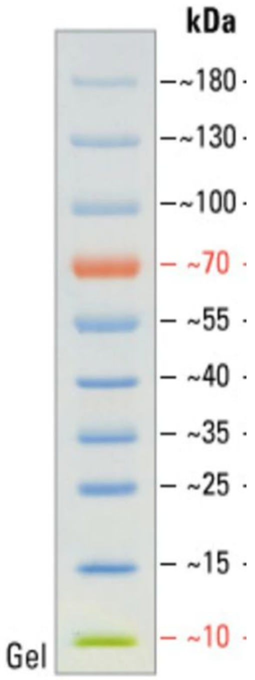

SDS-PAGE band profile of the PageRuler Prestained Protein Ladder from ThermoFisher Scientific

5. General Procedure for the biocatalyzed synthesis of quinolines 2

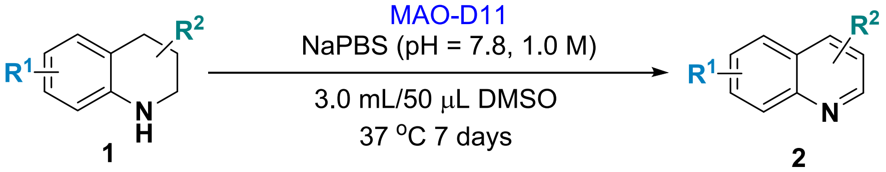

In a Falcon tube (15 mL), freeze-dried whole cells of *E.coli* expressing recombinant monoamine oxidase MAO-D11 (190 mg obtained from bulk production) or pure MAO-N D11 were suspended in buffer (Na_2HPO_4/NaH_2PO_4, pH = 7.8, 1.0 M) (3.0 mL). Thereafter, the appropriate 1,2,3,4-tetrahydroquinoline substrates (0.2 mmol) dissolved in DMSO (50 μL) was added. After addition, the mixture was incubated at 37 ºC and shaken at 160 rpm for 7 days. Then, the reaction mixture was extracted with ethyl acetate (3 x 3.0 mL) and centrifuged at 4000 x g for 10 minutes. The organic layers were then separated and combined and then dried over anhydrous $MgSO_4$. After filtration and removal of the solvents in vacuo, the crude product was analyzed through ^{1}H-NMR and the conversion value was determined by integration. Taking **2b** and **2e** (1 mmol scale) as example, the crude products were finally purified by column chromatography (hexane/EtOAC = 50/1) to afford the pure quinoline products.

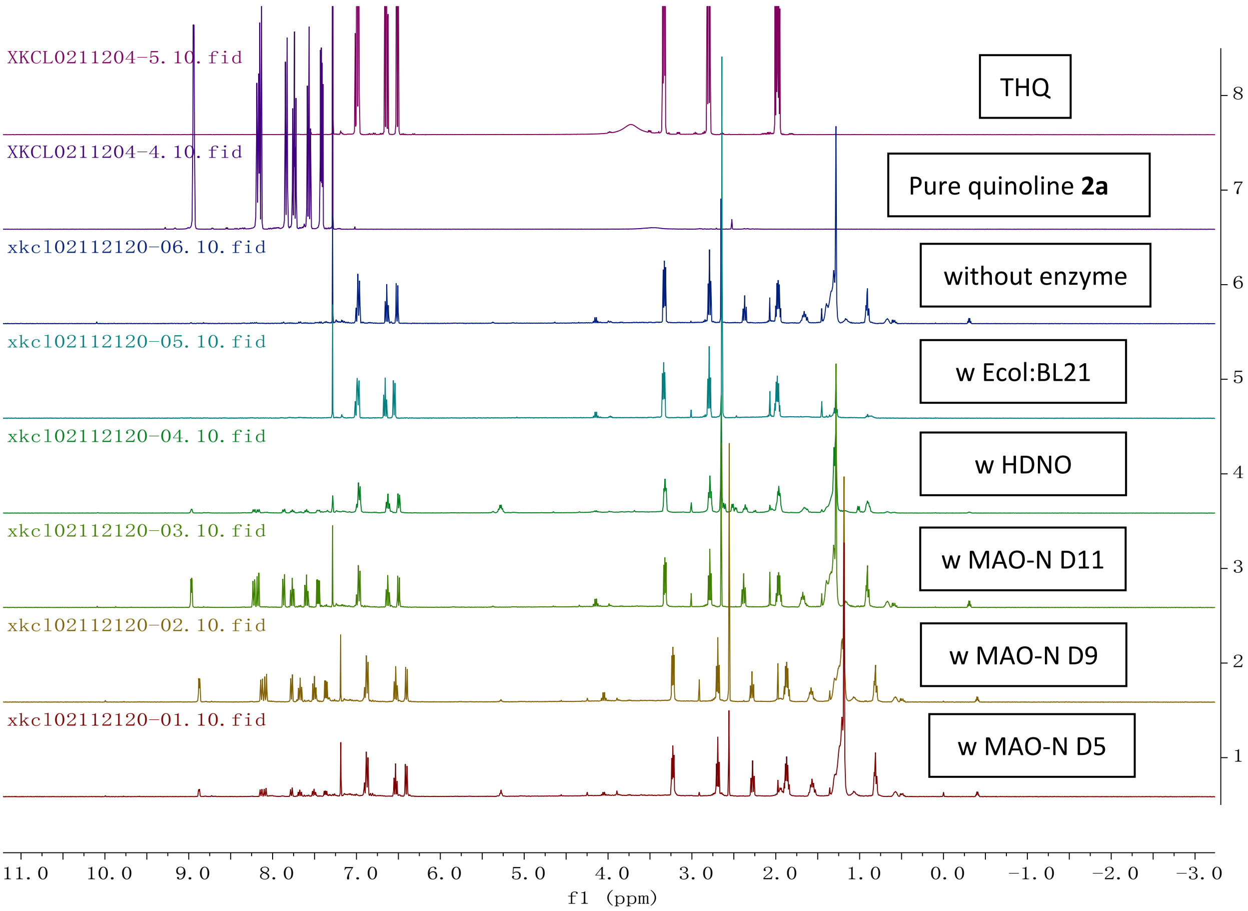

Figure S1. ^{1}H-NMR spectra for the crude product **2a** in $CDCl_3$ for conversion calculation

6. General Procedure for the biocatalyzed synthesis of quinoliniums 4 and quinolones 5

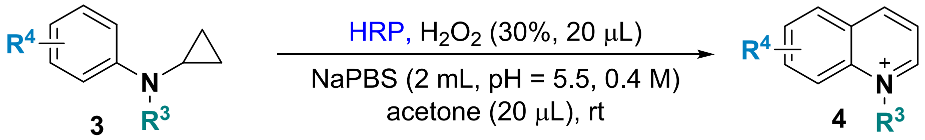

In a glass rolled rim vial (5 mL) equipped with a magnetic stirring bar, *N*-cyclopropyl-*N*-methylaniline **3** was suspended in buffer (Na_2HPO_4/NaH_2PO_4, pH = 5.5, 0.4 M) (2.0 mL). Thereafter, 20 µL acetone, 100 µL HRP solution (4 mg per 1 mL buffer, 240 U/mg) and 20 µL H_2O_2 (30%) were added successively, while stirring. The reaction mixture was stirred at room temperature for 0.5 h and then additional 50µL HRP solution was added. The reaction was detected by TLC and the crude product was analyzed through ^{1}H-NMR and the yield was calculated by integration with sodium 4-methylbenzenesulfonate as internal standard.

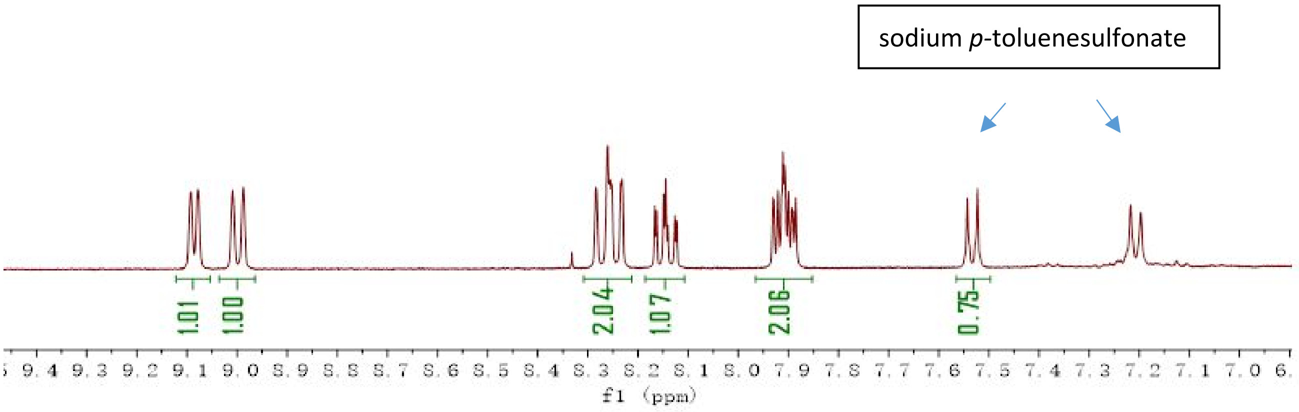

Figure S2. ^{1}H-NMR spectra for **4a** in D_2O with sodium 4-methylbenzenesulfonate as internal standard

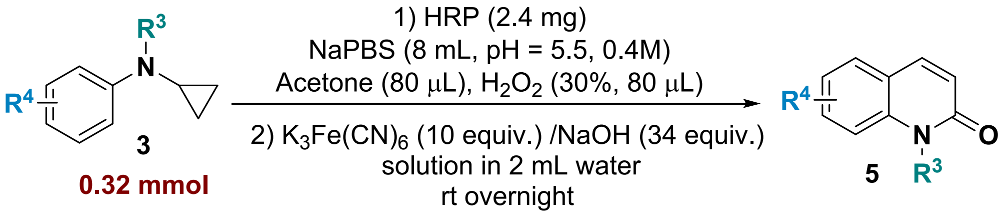

Step 1: Following the previous procedure, the scale-up of the reaction to 0.32 mmol was carried out accordingly. However, solid HRP (2.4 mg) was added directly to the reaction mixture.

Step 2: After the full consumption the starting materials **3** detected by TLC, $K_3Fe(CN)_6$ (10 equiv.) and NaOH (34 equiv.) in 2 mL water were added to the reaction mixture. The obtained suspension was stirred at room temperature overnight. The reaction mixture was extracted with EtOAc (3 x 20 mL) and the combined organic layer was washed with saturated salt solution and water. Then the organic layer was dried over anhydrous $MgSO_4$. After filtration and removal of the solvents in vacuo, the crude product was purified by column chromatography (hexance/EtOAc = 40/1).

7. *In Situ* EPR Experiments

The X-band continuous-wave EPR spectra were recorded on a Magnettech ESR5000 spectrometer (Bruker) equipped with a TCH04 temperature controller and a quartz Dewar insert.

For each experiment, the microwave power was set to 10 mW and a field modulation of 1 mT at 100 kHz was used; 4 traces recorded with a field sweep rate of 1.67 mT/s were accumulated.

The EPR signals clearly indicate the involvement of a radical in this process, which disappeared without **3a**. Based on these results, this EPR signals presumably should be the corresponding radical from HRP. However, the EPR signals were unable to be precisely identified due to their insufficient intensity.

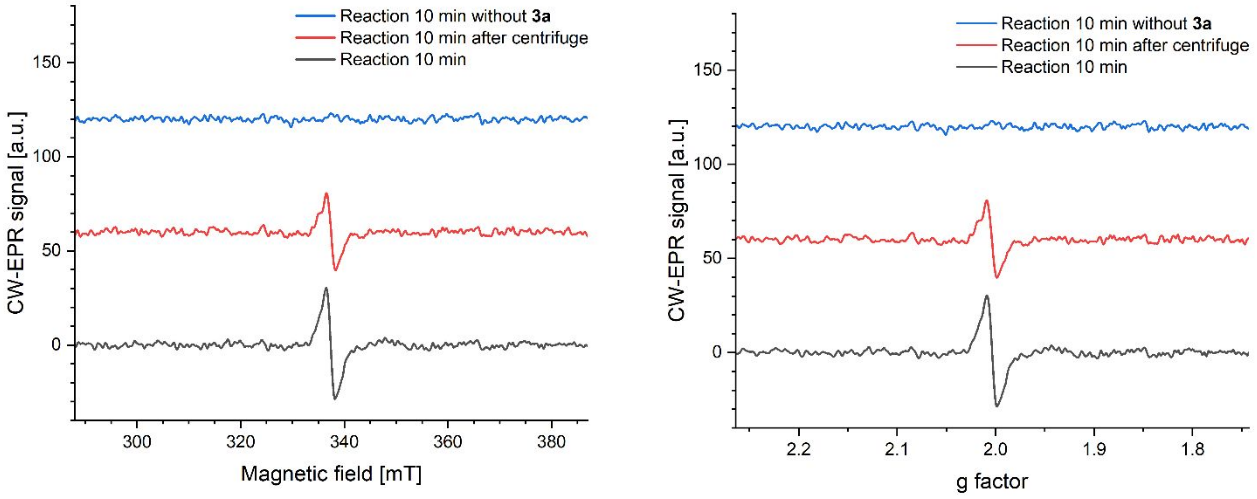

Figure S3. ESR spectrum of the HRP biocatalyzed cyclization/aromatization of **3a**

8. Computation Methods

All molecular docking studies were performed on a Viglen Genie Intel®CoreTM i7-3770 vPro CPU@ 3.40 GHz x 8 running Ubuntu 18.04. Molecular Operating Environment (MOE) 2022.10, Maestro (Schrödinger Release 2020-2) and Multiwfn were used as molecular modelling software.[1,2,3] The MAO-N D11 structure was downloaded from the PDB data bank (http://www.rcsb.org/; PDB code 3ZDN). The protein was pre-processed using the Schrödinger Protein Preparation Wizard by assigning bond orders, adding hydrogens and performing a restrained energy minimisation of the added hydrogens using the OPLS_2005 force field. Ligand structures were built with MOE and then prepared using the Maestro LigPrep tool by energy minimising the structures (OPLS_2005 force filed), generating possible ionization states at pH 7±2, generating tautomers and low-energy ring conformers. A 11 Å docking grid (inner-box 10 Å and outer-box 21 Å) was prepared using as centroid the co-crystallized FAD. Molecular docking studies were performed using Glide SP precision keeping the default parameters and setting 10 as number of output poses per input ligand to include in the solution. The output poses were saved as mol2 file. The docking results were visually inspected for their ability to bind the active site in MOE.

Quantum-chemical computations were performed using Gaussian.[3] Molecular structures were optimised at the B3LYP/6-31G(d) level of theory, in the gas phase, while the ESP was calculated using a constant electronic density of 0.002 au. The ESP values were shown by means of coloured maps on isodensity surface (range between -4.0e^{-2} and 4.0e^{-2} a.u). The nucleophilicity index N prediction was performed using Multiwfn based on the HOMO energies is obtained within the Kohn–Sham scheme, and defined as $N = E_{HOMO(Nu)} - E_{HOMO(TCE)}$[5].

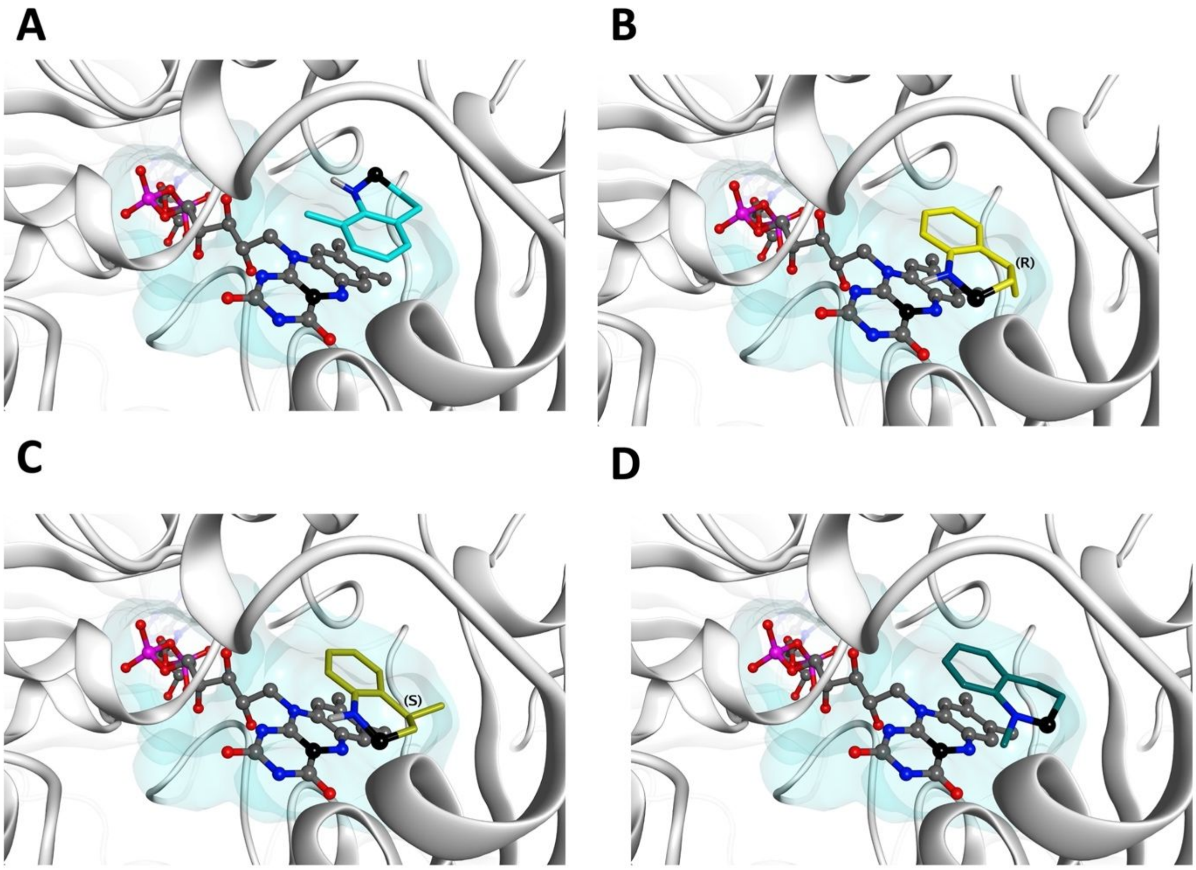

Figure S4. Proposed binding modes for compounds (A) **1k**, (B) **(*R*)-1m**, (C) **(*S*)-1m** and (D) *N*-Met-**1a** in the MAO-N D11 catalytic site (PDB ID: 3ZDN). Carbon atoms of compound **1k** are shown in turquoise, compound **(*R*)-1m** in yellow, compound **(*S*)-1m** in gold and compound *N*-Met-**1a** in teal. The binding area of the catalytic site is represented as a transparent surface. FAD is represented as a ball-and-stick model. Nitrogen atoms of **1k**, **(*R*)-1m**, **(*S*)-1m**, *N*-Met-**1a** and FAD are shown in blue. The α-methylene group is shown as black ball.

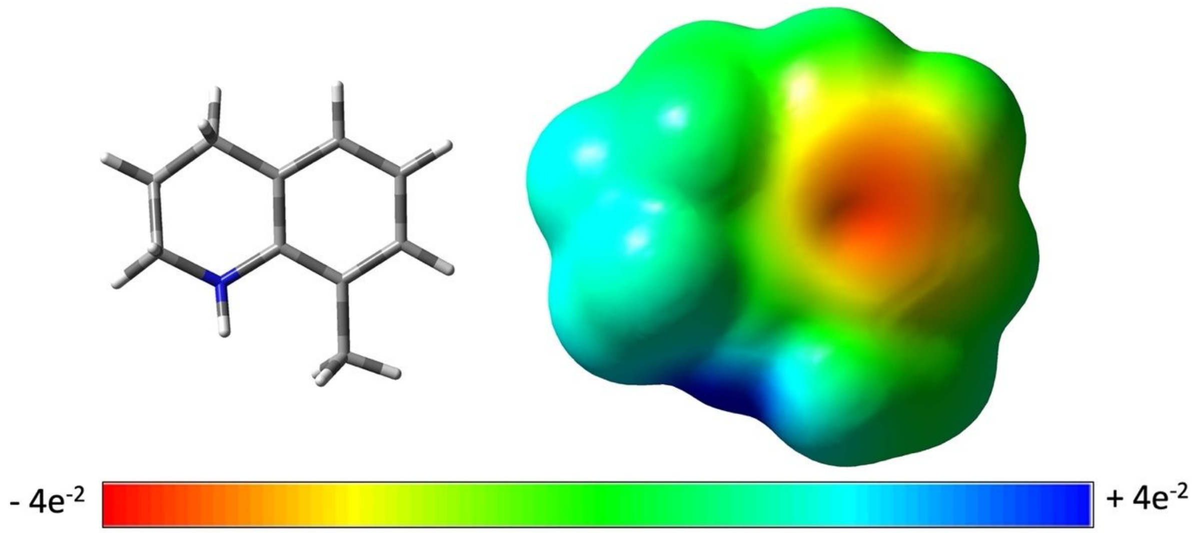

Figure S5. Electrostatic potential surface (EPS) for **1k**.

9. Characterization data for the new compounds

References for known compounds. The data of the known quinoline and quinolinium compounds prepared in this work were compared and found in agreement with those reported in literature. The references for the known compounds are reported in the Table S1.

Table S1. References for known compounds.

Compd	Structure	Reference	Compd	Structure	Reference
**2a**	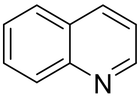	Commercially available	**2b**	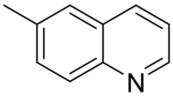	6
**2c**	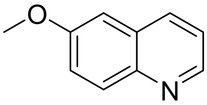	7	**2d**	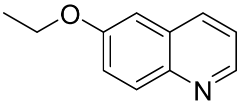	8
**2e**	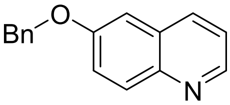	9	**2g**	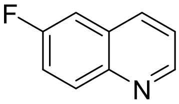	10
**2h**	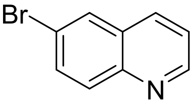	11	**2i**	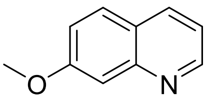	12
**2j**	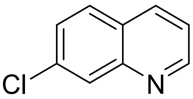	13	**2k**	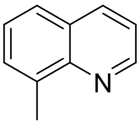	14
**2l**	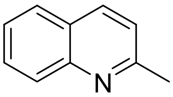	10	**2m**	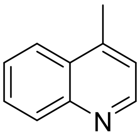	7
**3a**	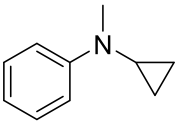	15	**3n**	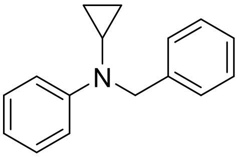	16
**4a**	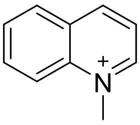	17	**4b**	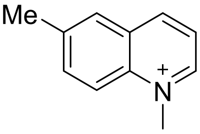	18
**4c**	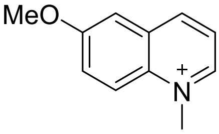	18	**4d**	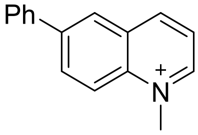	19
**4e**	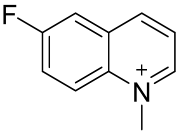	18	**4f**	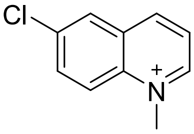	18

**4g**	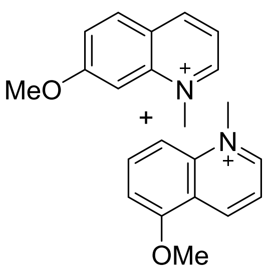	20	**4h**	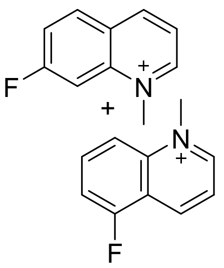	21
**4i**	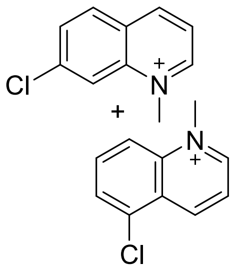	21	**4k**	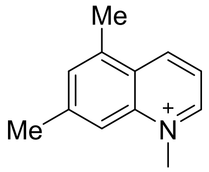	22
**4m**	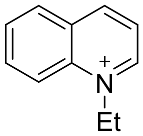	21	**4n**	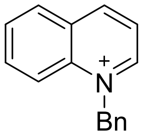	23

Characterization of new compounds

6-Ethoxy-1,2,3,4-tetrahydroquinoline 1d

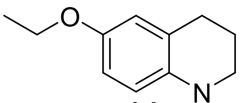

1d

^{1}H NMR (400 MHz, Chloroform-*d*) δ 6.64 – 6.54 (m, 2H), 6.44 (d, *J* = 8.4 Hz, 1H), 3.94 (q, *J* = 7.0 Hz, 2H), 3.40 – 3.19 (m, 2H), 2.75 (t, *J* = 6.5 Hz, 2H), 1.93 (p, *J* = 6.2 Hz, 2H), 1.36 (t, *J* = 7.0 Hz, 3H) ppm; **^{13}C NMR (100 MHz, Chloroform-*d*)** δ 151.2, 138.9, 122.9, 116.0, 115.6, 113.8, 64.2, 42.4, 27.2, 22.6, 15.1 ppm; **HRMS (ESI)** m/z: $[M+H]^+$ Calcd for $C_{11}H_{16}ON^+$ 178.1226, Found 178.1219.

6-(Benzyloxy)-1,2,3,4-tetrahydroquinoline 1e

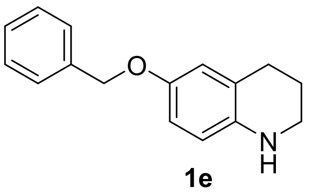

1e

^{1}H NMR (400 MHz, Chloroform-*d*) δ 7.43 (d, *J* = 7.4 Hz, 2H), 7.38 (t, *J* = 8.1 Hz, 2H), 7.31 (t, *J* = 7.2 Hz, 1H), 6.71 – 6.62 (m, 2H), 6.45 (d, *J* = 8.2 Hz, 1H), 4.98 (s, 2H), 3.37 – 3.14 (m, 2H), 2.76 (t, *J* = 6.5 Hz, 2H), 1.93 (p, *J* = 6.2 Hz, 2H) ppm; **^{13}C NMR (100 MHz, Chloroform-*d*)** δ 151.1, 139.2, 137.8, 128.6, 127.8 , 127.6, 122.9, 116.3, 115.5, 114.1, 70.9, 42.4, 27.2, 22.5 ppm; **HRMS (ESI)** m/z: $[M+H]^+$ Calcd for $C_{16}H_{18}ON^+$ 240.1383, Found 240.1376.

6-Hydroxhyquinoline 2f

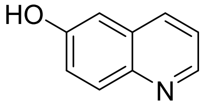

2f

^{1}H NMR (400 MHz, Acetone-d_6) δ 9.18 (*br* s, 1H), 8.70 (dd, *J* = 4.2, 1.6 Hz, 1H), 8.10 (dd, *J* = 8.3, 1.6 Hz, 1H), 7.93 (d, *J* = 9.1 Hz, 1H), 7.43 – 7.33 (m, 2H), 7.22 (d, *J* = 2.7 Hz, 1H) ppm; **^{13}C NMR (100 MHz, Acetone-d_6)** δ 156.5, 148.2, 144.8, 135.0, 131.7, 130.6, 122.6, 122.2, 109.4 ppm; **HRMS (ESI)** m/z: $[M+H]^+$ Calcd for $C_9H_8ON^+$ 146.0600, Found 146.0596.

***N*-Cyclopropyl-*N*-4-dimethylaniline 3b**

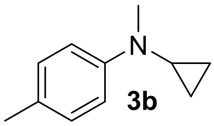

3b

^{1}H NMR (400 MHz, Chloroform-*d*) δ 7.17 – 7.05 (m, 2H), 7.03 – 6.82 (m, 2H), 3.01 (s, 3H), 2.50 – 2.34 (m, 1H), 2.33 (s, 3H), 0.95 – 0.75 (m, 2H), 0.75 – 0.41 (m, 2H) ppm; **^{13}C NMR (100 MHz, Chloroform-*d*)** δ 148.9, 129.4, 126.8, 114.2, 39.6, 33.6, 20.3, 9.0 ppm; **HRMS (ESI)** m/z: $[M+H]^+$ Calcd for $C_{11}H_{16}N^+$ 162.1277, Found 162.1272.

***N*-Cyclopropyl-4-methoxy-*N*-methylaniline 3c**

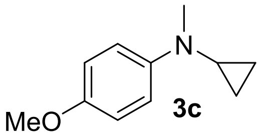

3c

^{1}H NMR (400 MHz, Chloroform-*d*) δ 7.01 – 6.81 (m, 2H), 6.76 – 6.73 (m, 2H), 3.66 (s, 3H), 2.83 (s, 3H), 2.22 – 2.03 (m, 1H), 0.77 – 0.58 (m, 2H), 0.55 – 0.37 (m, 2H) ppm; **^{13}C NMR (100 MHz, Chloroform-*d*)** δ 152.3, 145.6, 115.5, 114.5, 55.8, 40.2, 34.1, 8.9 ppm; **HRMS (ESI)** m/z: $[M+H]^+$ Calcd for $C_{11}H_{16}ON^+$ 178.1226, Found 178.1220.

***N*-Cyclopropyl-4-phenyl-*N*-methylaniline 3d**

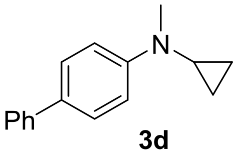

3d

^{1}H NMR (400 MHz, Chloroform-*d*) δ 7.52 – 7.46 (m, 2H), 7.44 – 7.37 (m, 2H), 7.36 – 7.27 (m, 2H), 7.22 – 7.15 (m, 1H), 7.01 – 6.91 (m, 2H), 2.93 (s, 3H), 2.35 (tt, *J* = 6.5, 3.8 Hz, 1H), 0.76 (td, *J* = 6.8, 4.7 Hz, 2H), 0.62 – 0.48 (m, 2H) ppm; **^{13}C NMR (100 MHz, Chloroform-*d*)** δ 150.2, 141.3, 130.2, 128.7, 127.5, 126.4, 126.1, 114.0, 39.1, 33.4, 9.1 ppm; **HRMS (ESI)** m/z: $[M+H]^+$ Calcd for $C_{16}H_{18}N^+$ 224.1434, Found 224.1426.

N-Cyclopropyl-4-fluoro-N-methylaniline 3e

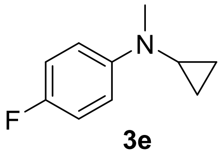

3e

^{1}H NMR (400 MHz, Chloroform-*d*) δ 6.98 – 6.68 (m, 4H), 2.85 (s, 3H), 2.22 (tt, J = 6.6, 3.7 Hz, 1H), 0.77 – 0.59 (m, 2H), 0.59 – 0.43 (m, 2H) ppm; **^{19}F NMR (376 MHz, Chloroform-*d*)** δ -128.35 ppm; **^{13}C NMR (100 MHz, Chloroform-*d*)** δ 156.03 (d, $^1J_{C\text{-}F}$ = 235.7 Hz), 147.50 (d, J = 1.9 Hz), 39.9, 33.8, 9.1 ppm; **HRMS (ESI)** m/z: $[M+H]^+$ Calcd for $C_{10}H_{13}NF^+$ 166.1027, Found 166.1022.

N-Cyclopropyl-4-chloro-N-methylaniline 3f

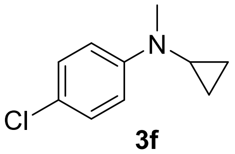

3f

^{1}H NMR (400 MHz, Chloroform-*d*) δ 7.21 (d, J = 9.2 Hz, 2H), 6.93 (d, J = 9.1 Hz, 2H), 2.99 (s, 3H), 2.40 (tdd, J = 6.5, 4.1, 3.4 Hz, 1H), 0.98 – 0.77 (m, 2H), 0.71 – 0.56 (m, 2H) ppm; **^{13}C NMR (100 MHz, Chloroform-*d*)** (100 MHz, Chloroform-*d*) δ 149.4, 128.6, 122.3, 114.9, 39.1, 33.4, 9.1 ppm; **HRMS (ESI)** m/z: $[M+H]^+$ Calcd for $C_{10}H_{13}N^{35}ClF^+$ 182.0731, Found 182.0726.

N-Cyclopropyl-3-methoxy-N-methylaniline 3g

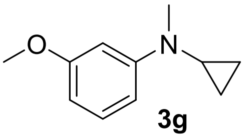

3g

^{1}H NMR (400 MHz, Chloroform-*d*) δ 7.07 (t, J = 8.1 Hz, 1H), 6.57 – 6.44 (m, 2H), 6.30 – 6.21 (m, 1H), 3.71 (s, 3H), 2.88 (s, 3H), 2.37 – 2.18 (m, 1H), 0.80 – 0.67 (m, 2H), 0.58 – 0.47 (m, 2H) ppm; **^{13}C NMR (100 MHz, Chloroform-*d*)** δ 160.5, 152.3, 129.5, 106.9, 102.3, 100.4, 55.1, 39.2, 33.4, 9.1 ppm; **HRMS (ESI)** m/z: $[M+H]^+$ Calcd for $C_{11}H_{16}ON^+$ 178.1226, Found 178.1221.

N-Cyclopropyl-3-fluoro-N-methylaniline 3h

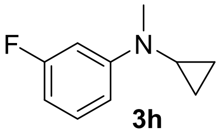

3h

^{1}H NMR (400 MHz, Chloroform-*d*) δ 7.12 – 6.94 (m, 1H), 6.69 – 6.49 (m, 2H), 6.36 (ddd, J = 9.4, 7.7, 2.1 Hz, 1H), 2.88 (s, 2H), 2.32 (tt, J = 6.5, 3.9 Hz, 1H), 0.89 – 0.65 (m, 2H), 0.55 (dd, J = 4.3, 2.3 Hz, 2H) ppm; **^{19}F NMR (376 MHz, Chloroform-*d*)** δ -112.96 ppm; **^{13}C NMR (100 MHz, Chloroform-*d*)** δ 163.8 (d, $^1J_{C\text{-}F}$ = 241.5 Hz), 152.6 (d, $^3J_{C\text{-}F}$ = 10.5 Hz), 129.7 (d, $^3J_{C\text{-}F}$ = 10.2 Hz), 109.2 (d, $^4J_{C\text{-}F}$ = 2.3 Hz), 103.7 (d, $^2J_{C\text{-}F}$ = 21.6 Hz), 100.7 (d, $^2J_{C\text{-}F}$ = 25.8 Hz), 38.9 , 33.3, 9.1 ppm; **HRMS (ESI)** m/z: $[M+H]^+$ Calcd for $C_{10}H_{13}NF^+$ 166.1027, Found 166.1022.

***N*-Cyclopropyl-3-chloro-*N*-methylaniline 3i**

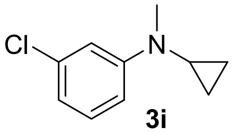

3i

^{1}H NMR (400 MHz, Chloroform-*d*) δ 7.06 (t, *J* = 8.1 Hz, 1H), 6.87 (t, *J* = 2.1 Hz, 1H), 6.76 (dd, *J* = 8.4, 1.7 Hz, 1H), 6.65 (dd, *J* = 7.8, 1.1 Hz, 1H), 2.89 (s, 3H), 2.33 (tt, *J* = 6.5, 3.8 Hz, 1H), 0.85 – 0.69 (m, 2H), 0.60 – 0.49 (m, 2H) ppm; **^{13}C NMR (100 MHz, Chloroform-*d*)** δ 151.8, 134.7, 129.7, 117.2, 113.6, 111.8, 38.9, 33.3, 9.1 ppm; **HRMS (ESI)** m/z: $[M+H]^+$ Calcd for $C_{10}H_{13}N^{35}ClF^+$ 182.0731, Found 182.0726.

***N*-Cyclopropyl-2,4-dimethyl-*N*-methylaniline 3j**

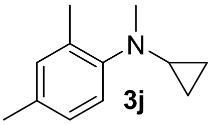

3j

^{1}H NMR (400 MHz, Chloroform-*d*) δ 7.07 (d, *J* = 7.9 Hz, 1H), 6.99 – 6.75 (m, 2H), 2.61 (s, 3H), 2.40 – 2.29 (m, 1H), 2.20 (s, 3H), 2.11 (s, 3H), 0.59 – 0.41 (m, 2H), 0.34 – 0.14 (m, 2H) ppm; **^{13}C NMR (100 MHz, Chloroform-*d*)** δ 149.7, 132.5, 132.0, 131.6, 126.8, 120.5, 43.4, 35.8, 20.8, 18.2, 8.1 ppm; **HRMS (ESI)** m/z: $[M+H]^+$ Calcd for $C_{12}H_{18}N^+$ 176.1434, Found 176.1428.

***N*-Cyclopropyl-3,5-dimethyl-*N*-methylaniline 3k**

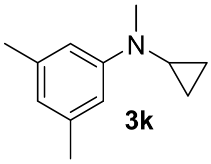

3k

^{1}H NMR (400 MHz, Chloroform-*d*) δ 6.67 (s, 2H), 6.48 (s, 1H), 2.98 (s, 3H), 2.44 – 2.33 (m, 1H), 2.32 (s, 6H), 0.93 – 0.73 (m, 2H), 0.69 – 0.55 (m, 2H) ppm; **^{13}C NMR (100 MHz, Chloroform-*d*)** δ 151.1, 138.4, 119.5, 111.9, 39.4, 33.4, 21.8, 9.1 ppm; **HRMS (ESI)** m/z: $[M+H]^+$ Calcd for $C_{12}H_{18}N^+$ 176.1434, Found 176.1428.

***N*-Cyclopropyl-*N*-methylnaphthalen-2-amine 3l**

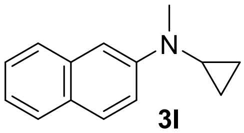

3l

^{1}H NMR (400 MHz, Chloroform-*d*) δ 7.91 – 7.65 (m, 3H), 7.47 – 7.33 (m, 2H), 7.28 – 7.14 (m, 2H), 3.13 (s, 3H), 2.61 – 2.39 (m, 1H), 1.09 – 0.82 (m, 2H), 0.82 – 0.35 (m, 2H) ppm; **^{13}C NMR (100 MHz, Chloroform-*d*)** δ 148.6, 134.8, 128.4, 127.5, 126.4, 126.1, 122.3, 117.1, 108.1, 39.5 , 33.7, 9.2 ppm; **HRMS (ESI)** m/z: $[M+H]^+$ Calcd for $C_{14}H_{16}N^+$ 198.1277, Found 198.1270.

N-Cyclopropyl-N-ethylaniline 3m

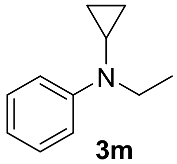

3m

^{1}H NMR (400 MHz, Chloroform-*d*) δ 7.36 – 7.13 (m, 2H), 7.00 (d, J = 8.2 Hz, 2H), 6.75 (t, J = 7.2 Hz, 1H), 3.49 (q, J = 7.0 Hz, 2H), 2.43 (tt, J = 6.9, 3.9 Hz, 1H), 1.11 (t, J = 7.0 Hz, 3H), 0.88 – 0.73 (m, 2H), 0.65 – 0.50 (m, 2H) ppm; **^{13}C NMR (100 MHz, Chloroform-*d*)** δ 149.3, 128.9, 117.2, 114.3, 45.4, 30.9, 11.6, 9.0 ppm; **HRMS (ESI)** m/z: $[M+H]^+$ Calcd for $C_{11}H_{16}N^+$ 162.1277, Found 162.1272.

1-Methylquinolin-2(1H)-one 5a

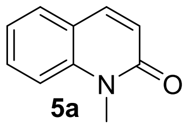

5a

^{1}H NMR (400 MHz, Chloroform-*d*) δ 7.66 (d, J = 9.5 Hz, 1H), 7.61 – 7.52 (m, 2H), 7.36 (d, J = 8.4 Hz, 1H), 7.23 (t, J = 7.5 Hz, 1H), 6.71 (d, J = 9.4 Hz, 1H), 3.72 (s, 3H) ppm; **^{13}C NMR (100 MHz, Chloroform-*d*)** δ 162.4, 140.1, 139.0, 130.7, 128.8, 122.2, 121.8, 120.8, 114.2, 29.5 ppm; **HRMS (ESI)** m/z: $[M+H]^+$ Calcd for $C_{10}H_{10}ON^+$ 160.0757, Found 160.0751.

6-Fluoro-1-methylquinolin-2(1H)-one 5e

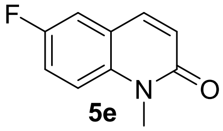

5e

^{1}H NMR (400 MHz, Chloroform-*d*) δ 7.60 (d, J = 9.5 Hz, 1H), 7.40 – 7.15 (m, 3H), 6.75 (d, J = 9.5 Hz, 1H), 3.71 (s, 3H) ppm; **^{19}F NMR (376 MHz, Chloroform-*d*)** δ -121.3 ppm; **^{13}C NMR (100 MHz, Chloroform-*d*)** δ 162.0, 157.8 (d, $^1J_{C-F}$ = 242.5 Hz), 138.0 (d, $^4J_{C-F}$ = 3.1 Hz), 136.7, 123.3, 121.5 (d, $^3J_{C-F}$ = 8.5 Hz), 118.4 (d, $^2J_{C-F}$ = 23.7 Hz), 115.8 (d, $^3J_{C-F}$ = 8.1 Hz), 113.8 (d, $^2J_{C-F}$ = 22.4 Hz), 29.8 ppm; **HRMS (ESI)** m/z: $[M+H]^+$ Calcd for $C_{10}H_9ONF^+$ 178.0663, Found 178.0657.

1,5,7-Trimethylquinolin-2(1H)-one 5k

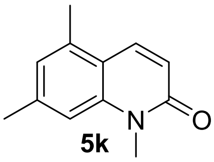

5k

^{1}H NMR (400 MHz, Chloroform-*d*) δ 7.85 (d, J = 9.7 Hz, 1H), 7.03 (s, 1H), 6.90 (s, 1H), 6.65 (d, J = 9.7 Hz, 1H), 3.70 (s, 3H), 2.52 (s, 3H), 2.45 (s, 3H) ppm; **^{13}C NMR (100 MHz, Chloroform-*d*)** δ 162.4, 141.0, 140.7, 136.0, 135.2, 125.3, 119.9, 117.3, 112.8, 29.7, 22.2, 19.0 ppm; **HRMS (ESI)** m/z: $[M+H]^+$ Calcd for $C_{12}H_{14}ON^+$ 188.1070, Found 188.1063.

4-Methylbenzo[f]quinolin-3(4H)-one 5l

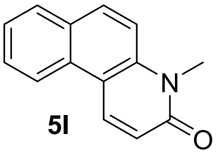

5l

¹H NMR (400 MHz, Chloroform-*d*) δ 8.56 (d, *J* = 9.7 Hz, 1H), 8.37 (d, *J* = 8.5 Hz, 1H), 8.02 (d, *J* = 9.2 Hz, 1H), 7.90 (d, *J* = 8.0 Hz, 1H), 7.68 (t, *J* = 7.7 Hz, 1H), 7.61 (d, *J* = 9.3 Hz, 1H), 7.54 (t, *J* = 7.5 Hz, 1H), 6.90 (d, *J* = 9.7 Hz, 1H) ppm; **^{13}C NMR (100 MHz, Chloroform-*d*)** δ 162.4, 139.2, 133.9, 132.1, 129.9, 129.0, 128.8, 128.2, 125.5, 121.6, 120.8, 114.8, 114.7, 30.3 ppm; **HRMS (ESI)** m/z: $[M+H]^+$ Calcd for $C_{14}H_{12}ON^+$ 210.2555, Found 210.0906.

1-Ethylquinolin-2(1H)-one 5m

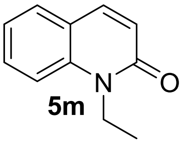

5m

¹H NMR (400 MHz, Chloroform-*d*) δ 7.63 (d, *J* = 9.6 Hz, 1H), 7.57 – 7.44 (m, 2H), 7.35 (d, *J* = 8.7 Hz, 1H), 7.18 (t, *J* = 7.5 Hz, 1H), 6.67 (d, *J* = 9.4 Hz, 1H), 4.33 (q, *J* = 7.1 Hz, 2H), 1.33 (t, *J* = 7.2 Hz, 3H) ppm; **^{13}C NMR (100 MHz, Chloroform-*d*)** δ 161.9, 139.1, 139.0, 130.7, 129.1, 122.0, 121.8, 121.0, 114.1, 37.3, 12.8 ppm; **HRMS (ESI)** m/z: $[M+H]^+$ Calcd for $C_{11}H_{12}ON^+$ 174.0913, Found 174.0908.

10. References

1. ULC, C. C. G. Molecular Operating Environment (MOE), 2022.10, 1010 Sherbooke St. West, Suite 910, Montreal, QC, Canada, H3A 2R7, 2018.
2. Schrödinger Release 2020-2: Maestro, Schrödinger, LLC, New York, NY, 2020.
3. Gaussian 09; Gaussian, Inc.: Pittsburgh, PA, 1998. http://www.gaussian.com/
4. Lu, T.; Chen, F., Multiwfn: A multifunctional wavefunction analyser. *J. Comput. Chem.* **2012**, *33*, 580-592.
5. Domingo, L. R.; Chamorro, E.; Pérez, P., Understanding the reactivity of captodative ethylenes in polar cycloaddition reactions. A theoretical study. *J. Org. Chem.* **2008**, *73* (12), 4615-4624.
6. Kim, K, D.; Lee, J. H., Visible-Light Photocatalyzed Deoxygenation of N-Heterocyclic N-Oxides. *Org. Lett.* **2018**, *20*(23), 7712-7716.
7. Wendlandt, A, E.; Stahl, S, S., Modular o-Quinone Catalyst System for Dehydrogenation of Tetrahydroquinolines under Ambient Conditions. *J. Am. Chem. Soc.* **2014**, *136*(34), 11910-11913.
8. Chang, G.; Hamanaka, E, S., McCarthy, P, A.; Truong, T.; Walker, F, J. US5362878 A 1994-11-08.
9. Solbak, S, M, Ø.; Zang, J.; Narayanan, D.; Høj, L, J.; Bucciarelli, S.; Softley, C.; Meier, S.; Langkilde, A, E.; Gotfredsen, C, H.; Sattler, M.; Bach, A., Developing Inhibitors of the p47phox–p22phox Protein–Protein Interaction by Fragment-Based Drug Discovery. *J. Med. Chem.* **2020**, *63*(3), 1156-1177.
10. He, K, H.; Tan, F, F.; Zhou, C, Z.; Zhou, G, J.; Yang, X, L.; Li, Y., Acceptorless dehydrogenation of

n-heterocycles by merging visible-light photoredox catalysis and cobalt catalysis. *Angew. Chem. Int. Ed.* **2017**, *56*(11), 3080-3084.

11. Losub, A, V.; Stahl, S, S., Catalytic aerobic dehydrogenation of nitrogen heterocycles using heterogeneous cobalt oxide supported on nitrogen-doped carbon. *Org. Lett.* **2015**, *17*(18), 4404-4407.

12. Zheng, M.; Shi, J.; Yuan, T.; Wang, X., Metal-Free Dehydrogenation of N-Heterocycles by Ternary h-BCN Nanosheets with Visible Light. *Angew. Chem. Int. Ed.* **2018**, *57*(19), 5487-5491.

13. Flanagan, J, C, A.; Dornan, L.; McLaughlin, M, G.; McCreanor, N, G.; Cook, M, J.; Muldoon, M, J., The synthesis of N-heterocycles via copper/TEMPO catalysed aerobic oxidation of amino alcohols. *Green Chem.* **2012**, *14*(5), 1281-1283.

14. Ge, D.; Hu, L.; Wang, J.; Li, X.; Qi, F.; Lu, J.; Cao, X.; Gu, H., Reversible hydrogenation–oxidative dehydrogenation of quinolines over a highly active Pt nanowire catalyst under mild conditions. *ChemCatChem* **2013**, *5*(8), 2183-2186.

15. Shaffer, C, L,; Morton, M, D.; Hanzlik, R, P., N-Dealkylation of an N-Cyclopropylamine by Horseradish Peroxidase. Fate of the Cyclopropyl Group. *J. Am. Chem. Soc.* **2001**, 123(35), 8502-8508.

16. Wimalasena, K.; Wickman, H, B.; Mahindaratne, M, P. D., Autocatalytic Radical Ring Opening of N-Cyclopropyl-N-phenylamines Under Aerobic Conditions − Exclusive Formation of the Unknown Oxygen Adducts, N-(1,2-Dioxolan-3-yl)-N-phenylamines. *Eur. J. Org. Chem.* **2001**, 20, 3811-3817.

17. Carreon, J, R.; Mahon, K, P.; Kelley, S, O., Thiazole orange− peptide conjugates: sensitivity of DNA binding to chemical structure. *Org. Lett.* **2004**, *6*(4), 517-519.

18. Li, S.; Li, W, J.; Yang, X. Sun, R.; Tang, J.; Zheng, X, L.; Yuan, M, L.; Li, R, X.; Chen, H.; Fu, H, Y., Selective direct C–H polyfluoroarylation of electron-deficient N-heterocyclic compounds. *Org. Chem. Front.* **2020**, *7*(23), 3887-3895.

19. Zhou, Y.; Liu, W.; Xing, Z.; Guan, J.; Song, Z.; Peng, Y., External-photocatalyst-free visible-light-mediated aerobic oxidation and 1,4-bisfunctionalization of N-alkyl isoquinolinium salts. *Org. Chem. Front.* **2020,** *7*(17), 2405-2413.

20. Zhu, J, K.; Gao, J, M.; Yang, C, J.; Shang, X, F.; Zhao, Z, M.; Lawoe, R, K.; Zhou, R.; Sun, Y.; Yin, X, D.; Liu, Y, Q., Design, synthesis, and antifungal evaluation of neocryptolepine derivatives against phytopathogenic fungi. *J. Agric. Food Chem.* **2020**, *68*(8), 2306-2315.

21. Tang, J.; Chen, X.; Zhao, C.; Li, W.; Li, S.; Zheng, X.; Yuan, M.; Fu, H.; Li, R.; Chen, H., Iodination/Amidation of the N-Alkyl (Iso)quinolinium Salts. *J. Org. Chem.* **2021**, *86*(1), 716-730.

22. Walther, B.; Heinrich, G., Acylation of indoles and quinolines by the Friedel-Crafts reaction. *Justus Liebigs Annalen der Chemie* **1941**, 549, 238-55.

23. Tan, Z.; Ci, C.; Yang, J.; Wu, Y.; Cao, L.; Jiang, H.; Zhang, M., Catalytic Conversion of N-Heteroaromatics to Functionalized Arylamines by Merging Hydrogen Transfer and Selective Coupling. ACS Catal. **2020**, *10*(9), 5243-5249.

11. Copies of NMR Spectra of new compounds

NMR spectra of 1d

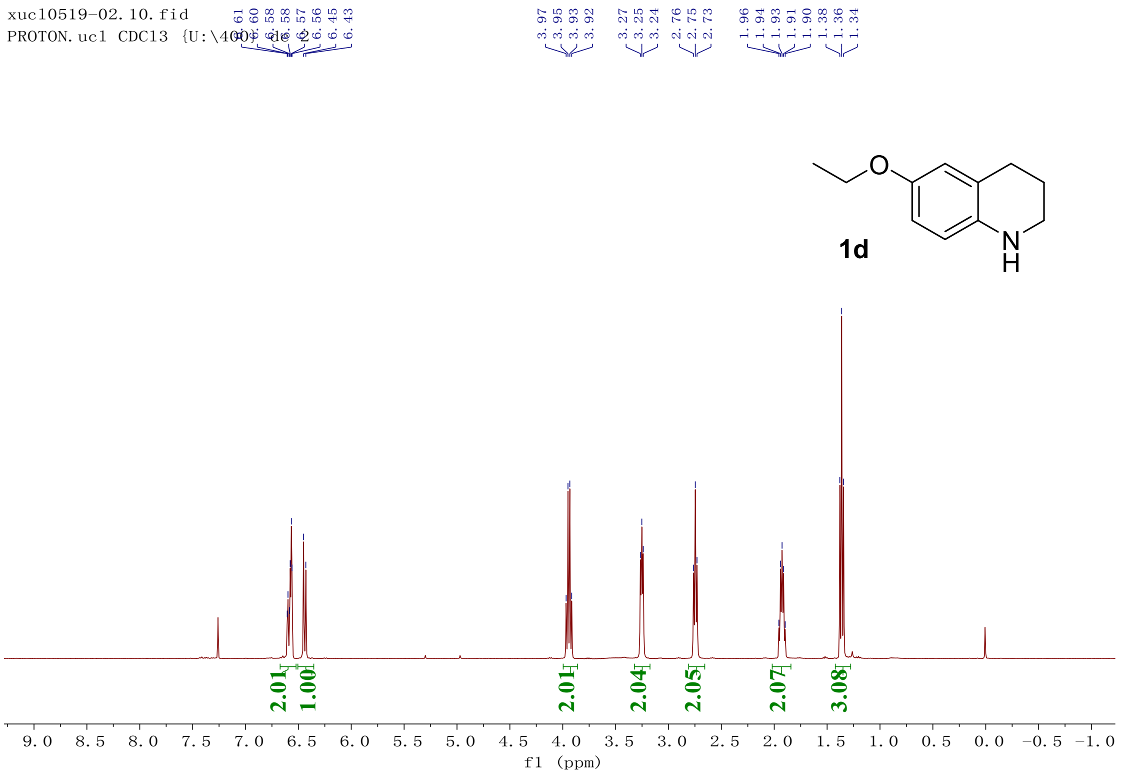

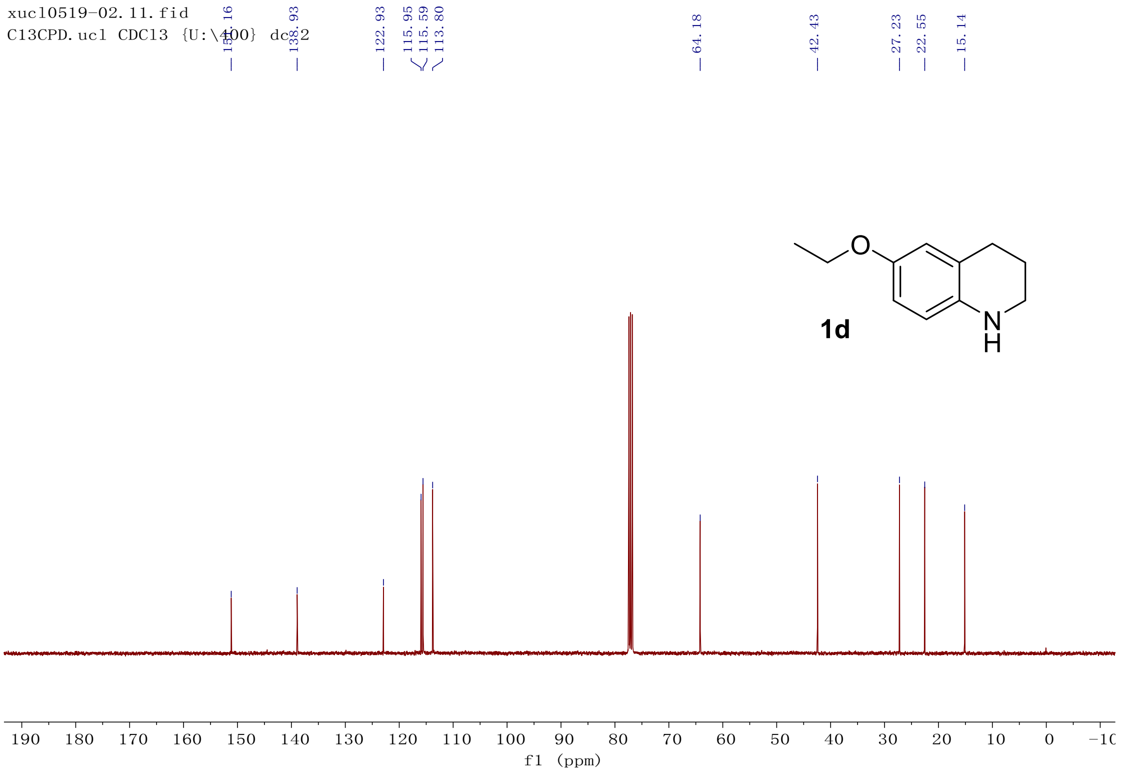

NMR spectra of 1e

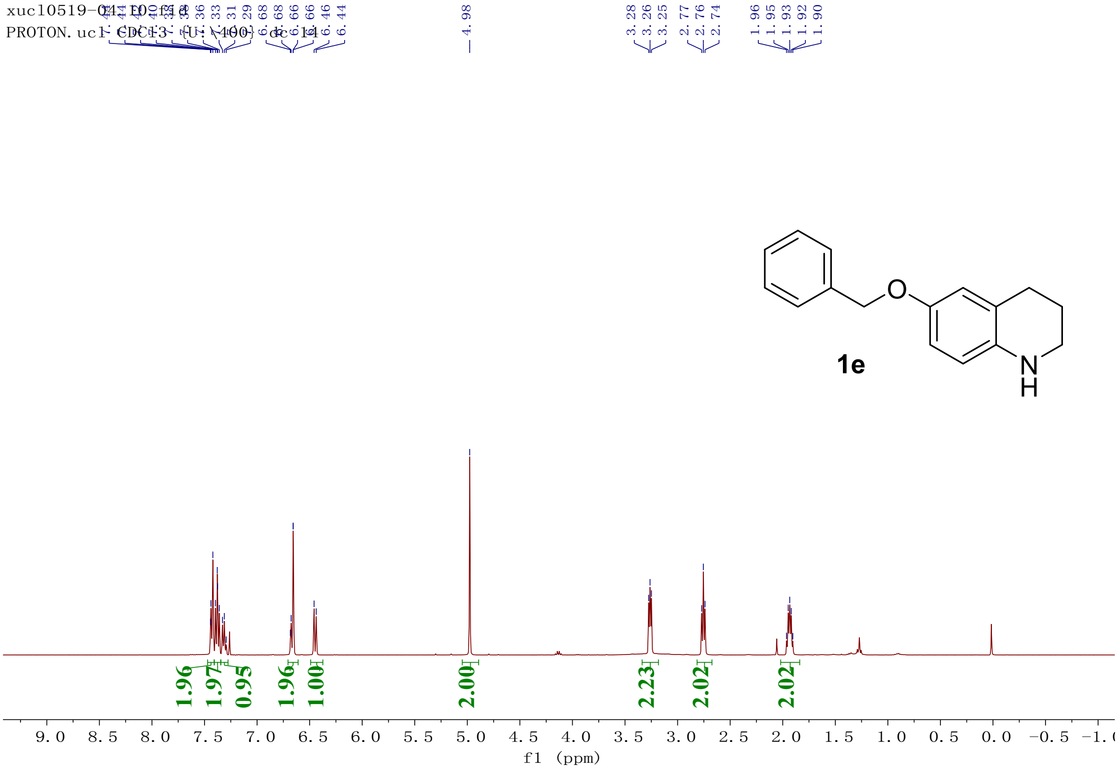

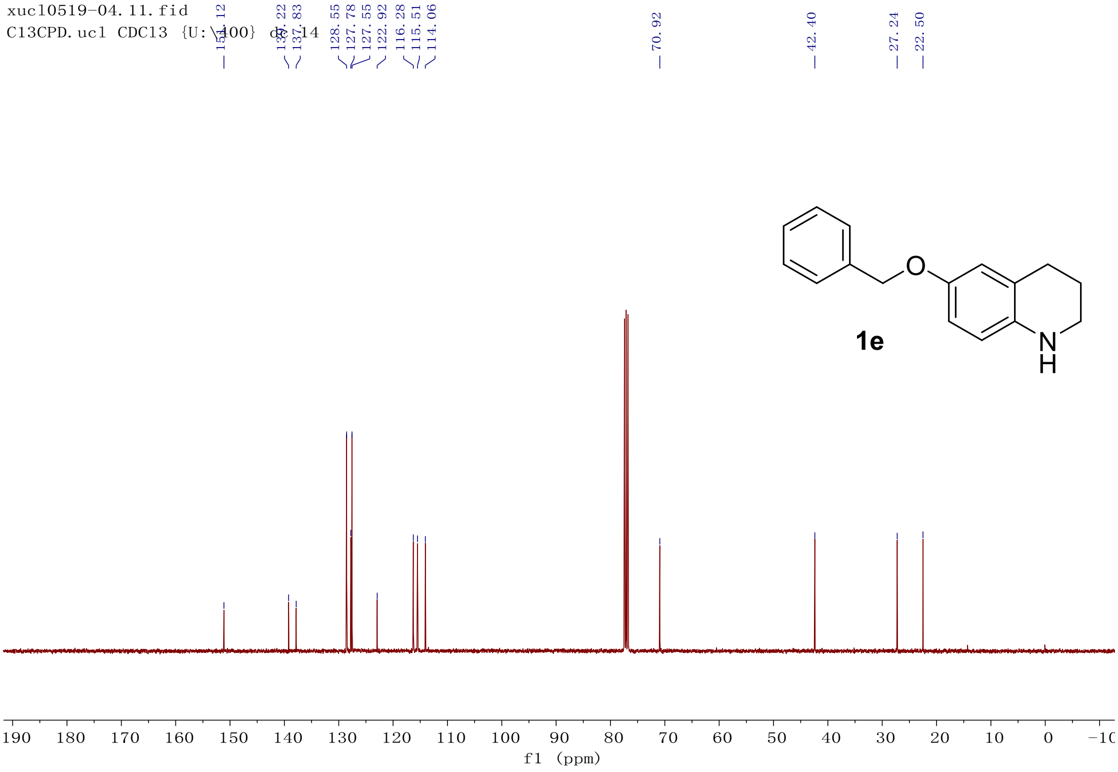

NMR spectra of 2f

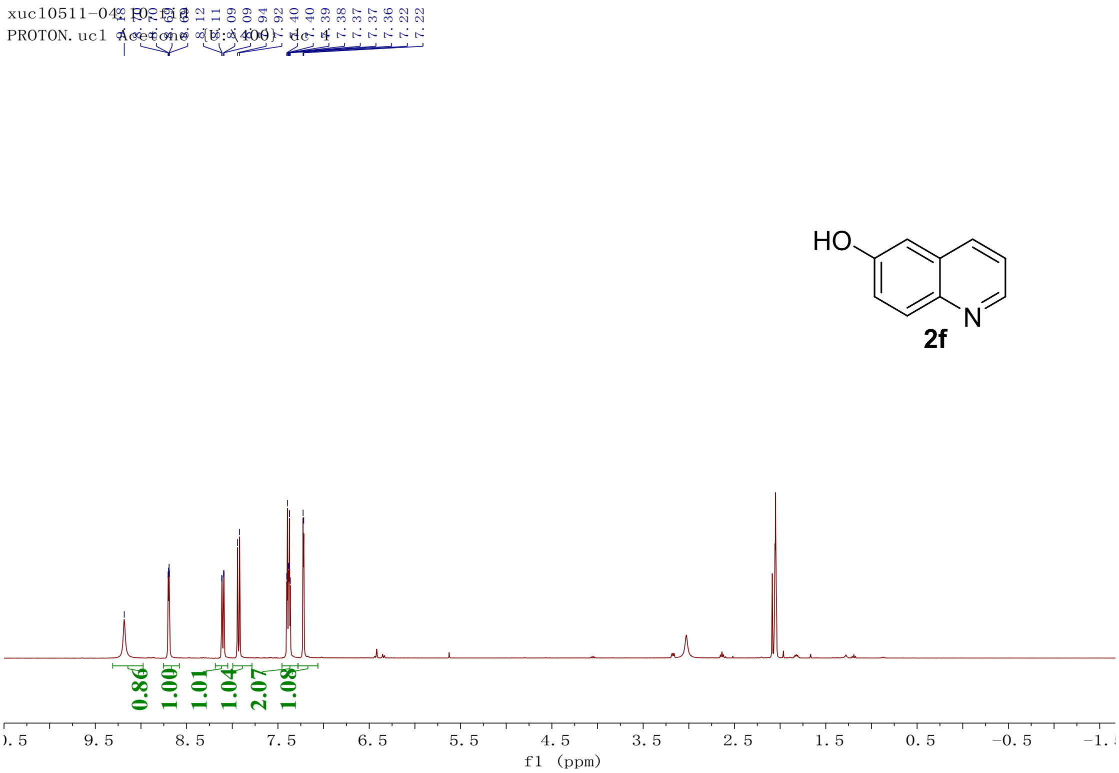

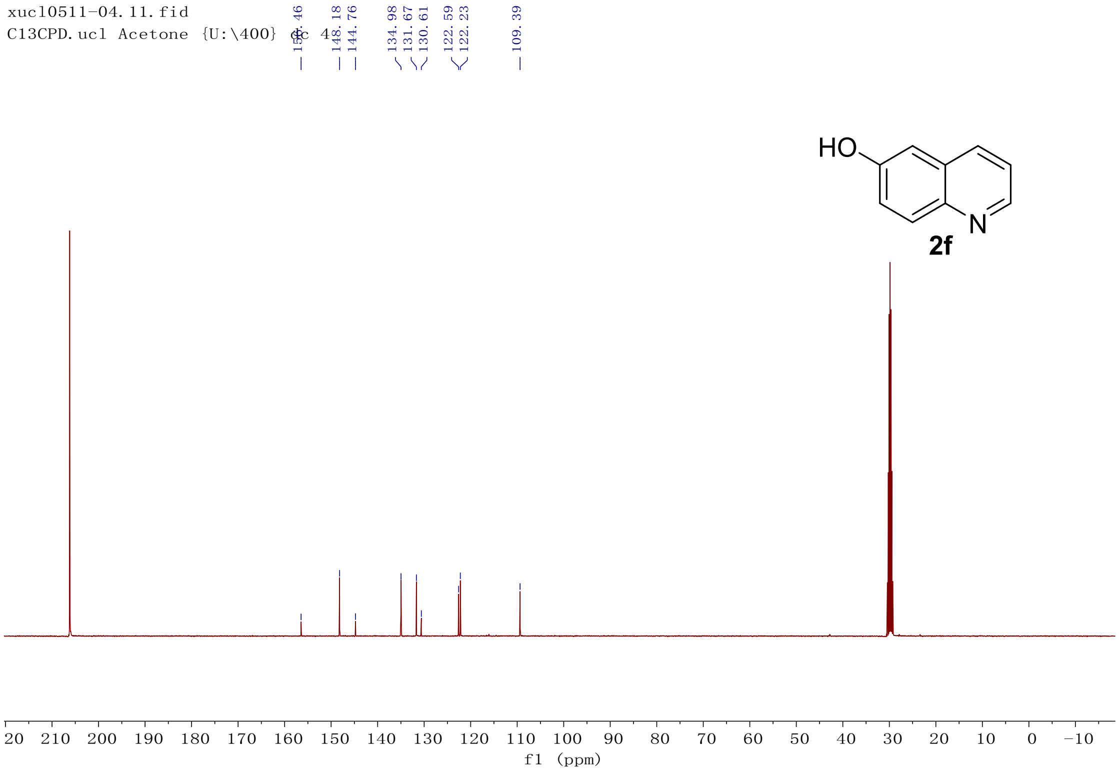

NMR spectra of 3b

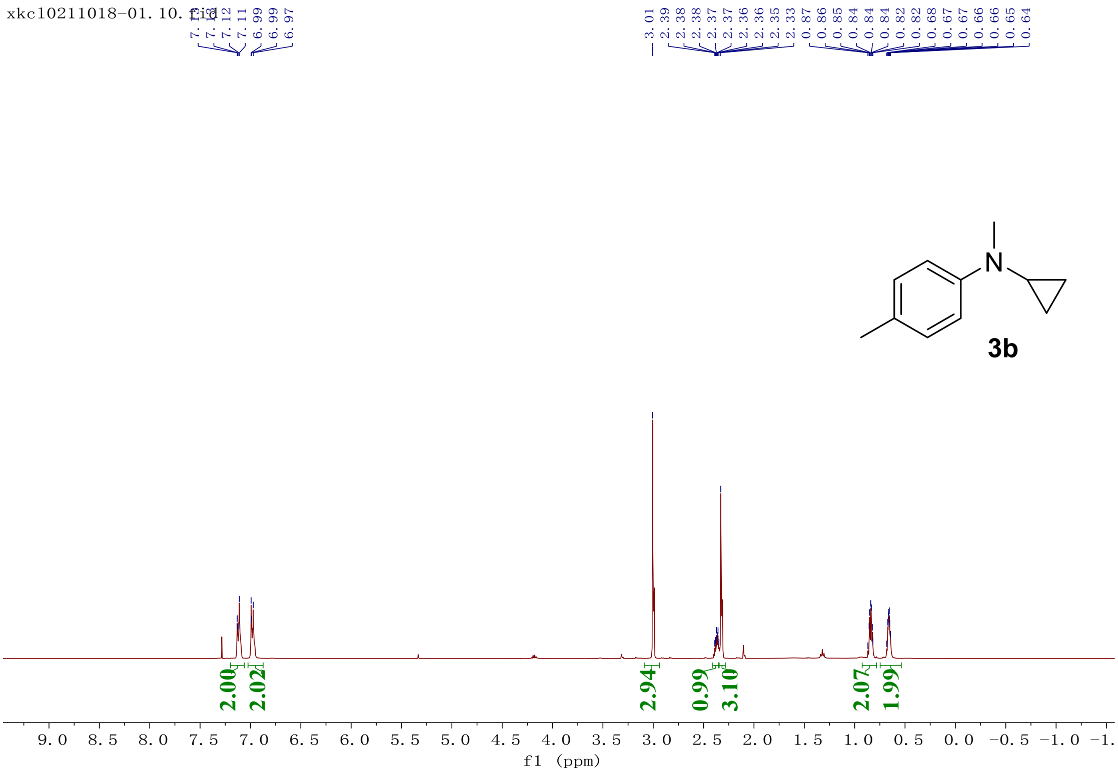

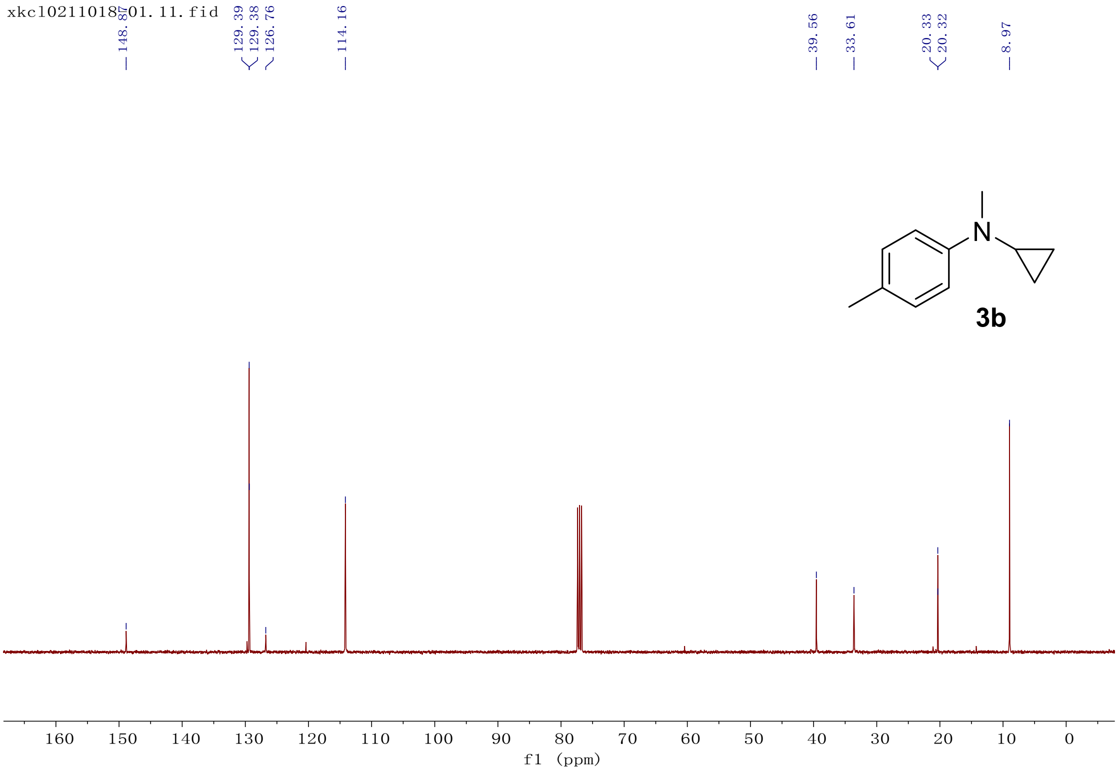

NMR spectra of 3c

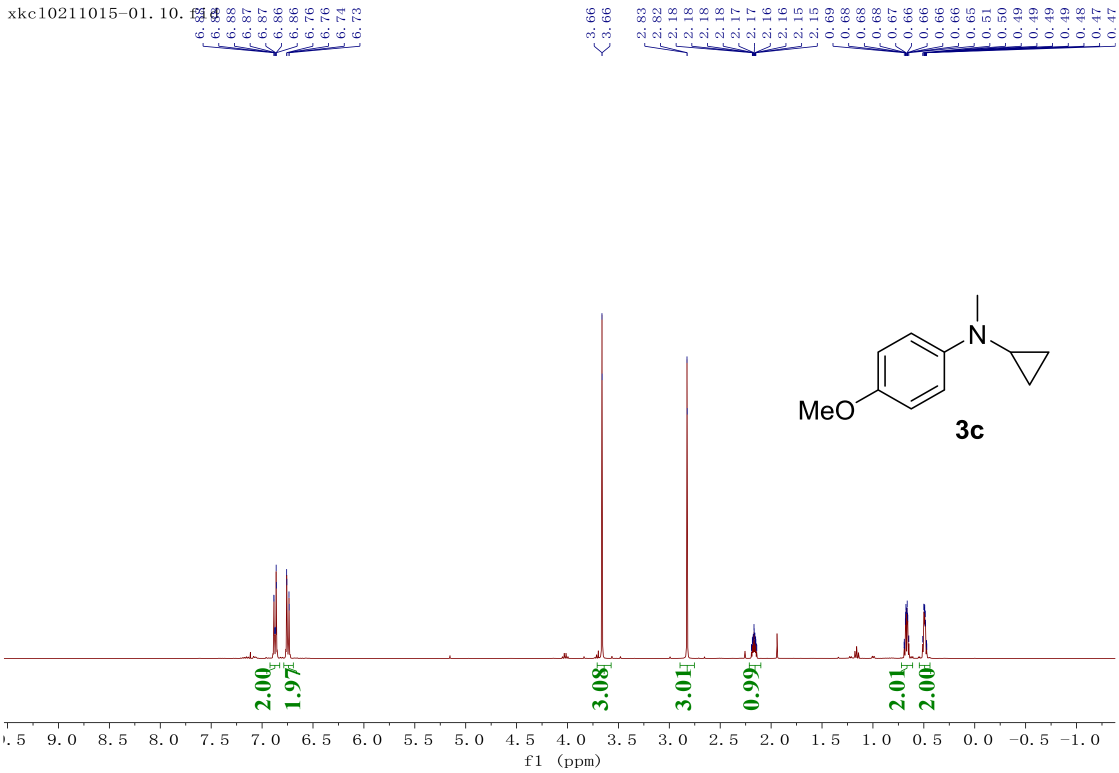

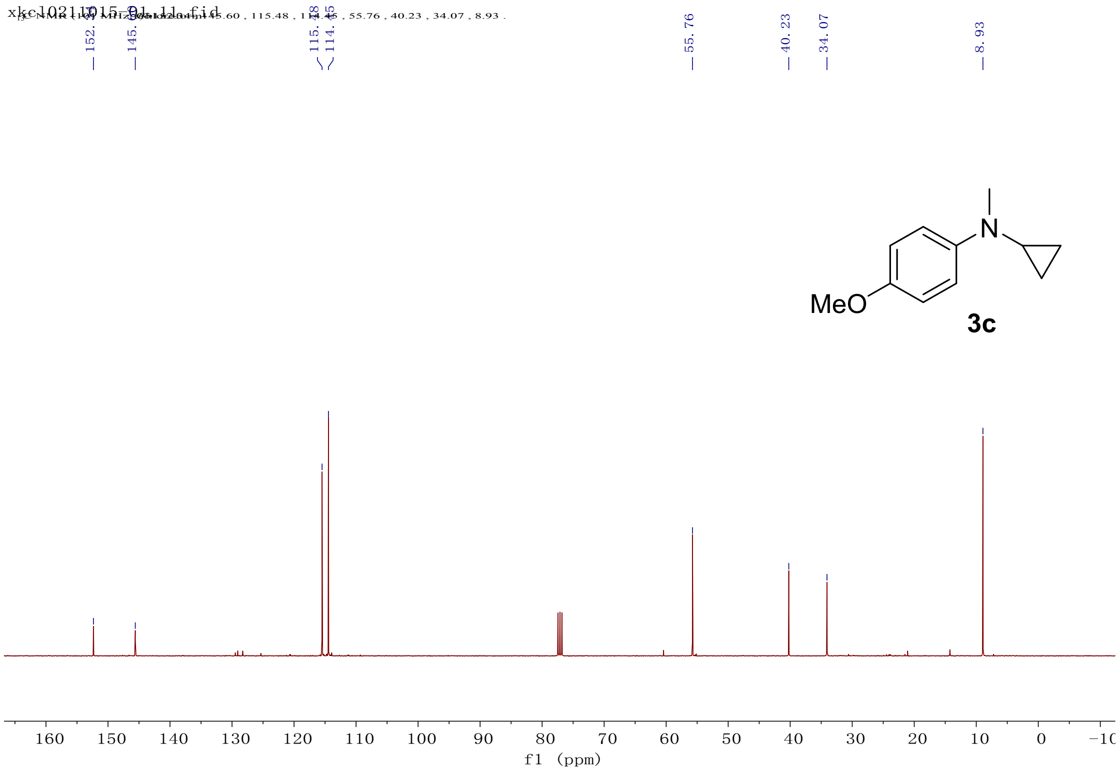

NMR spectra of 3d

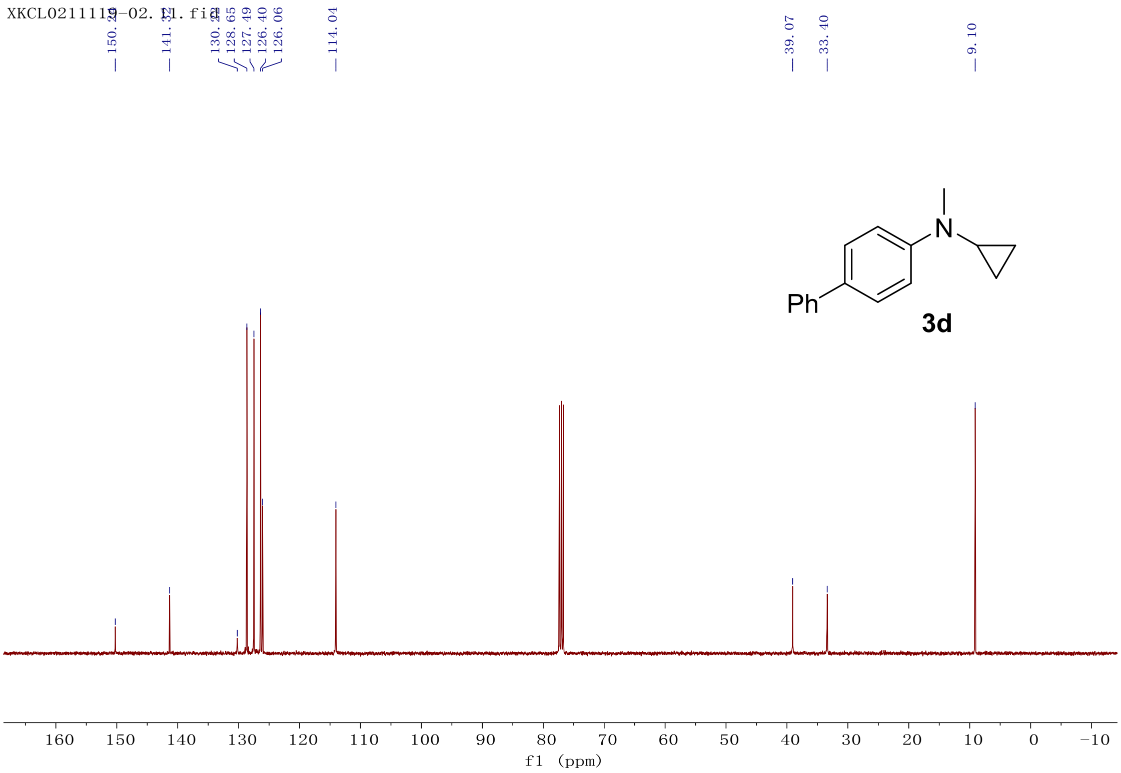

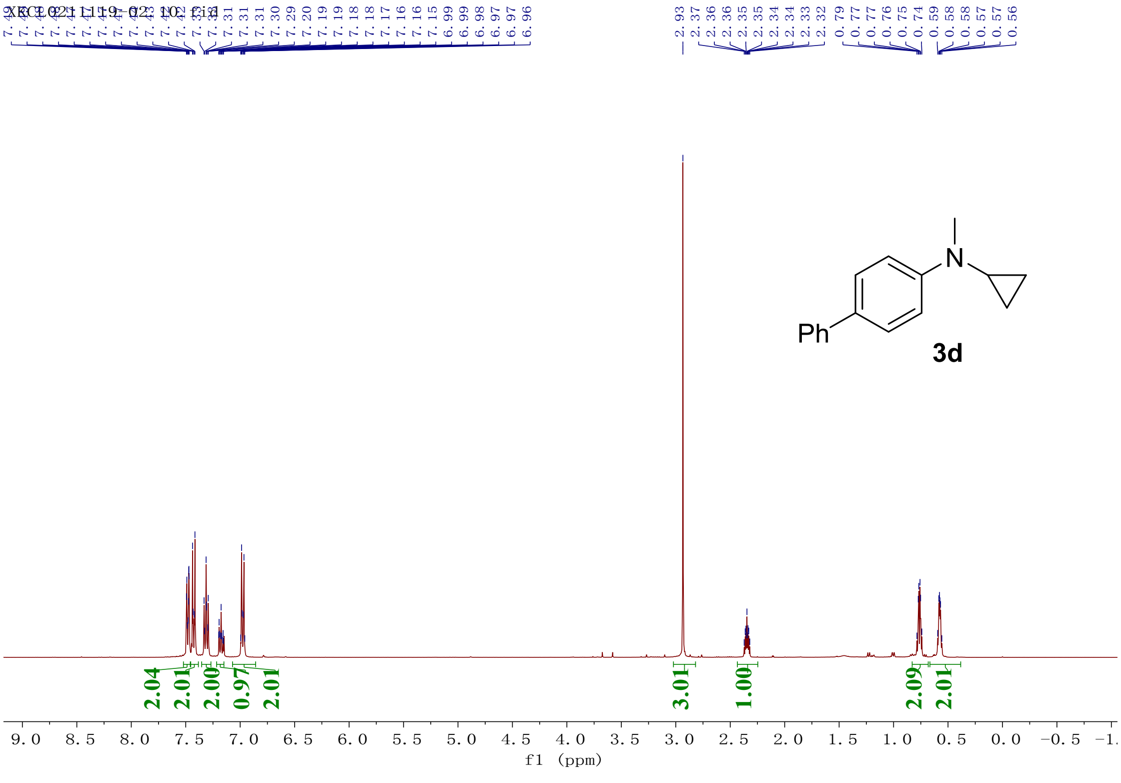

NMR spectra of 3e

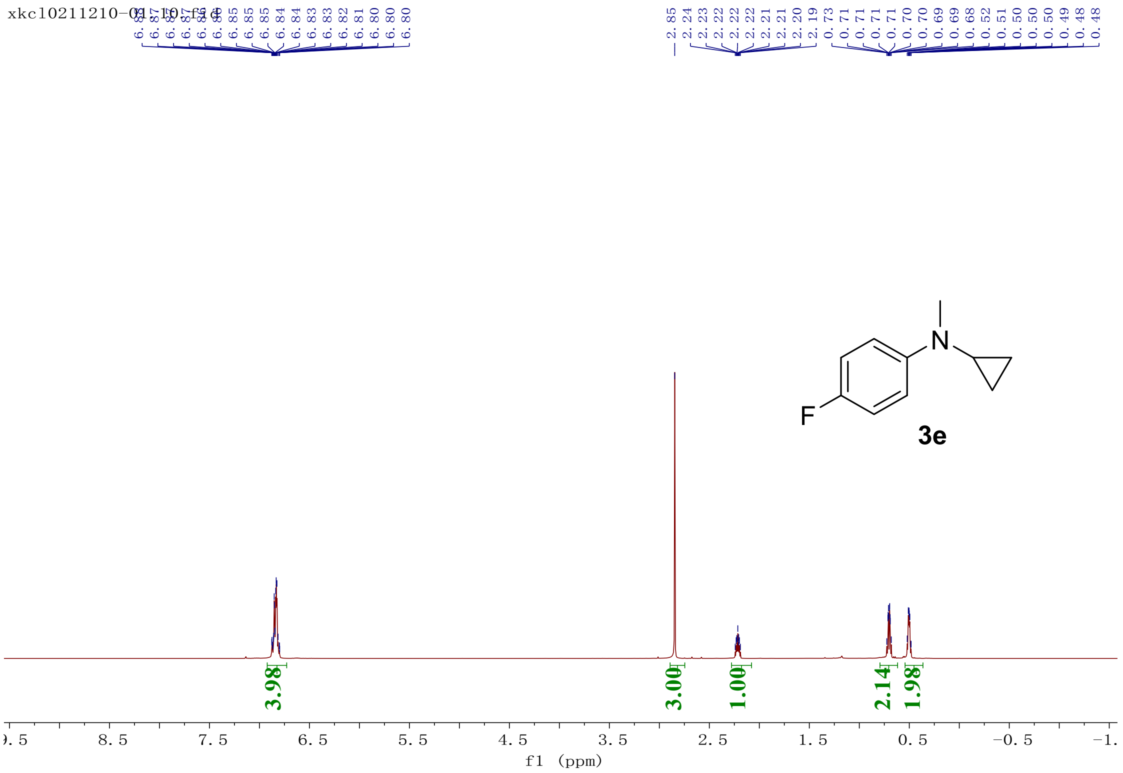

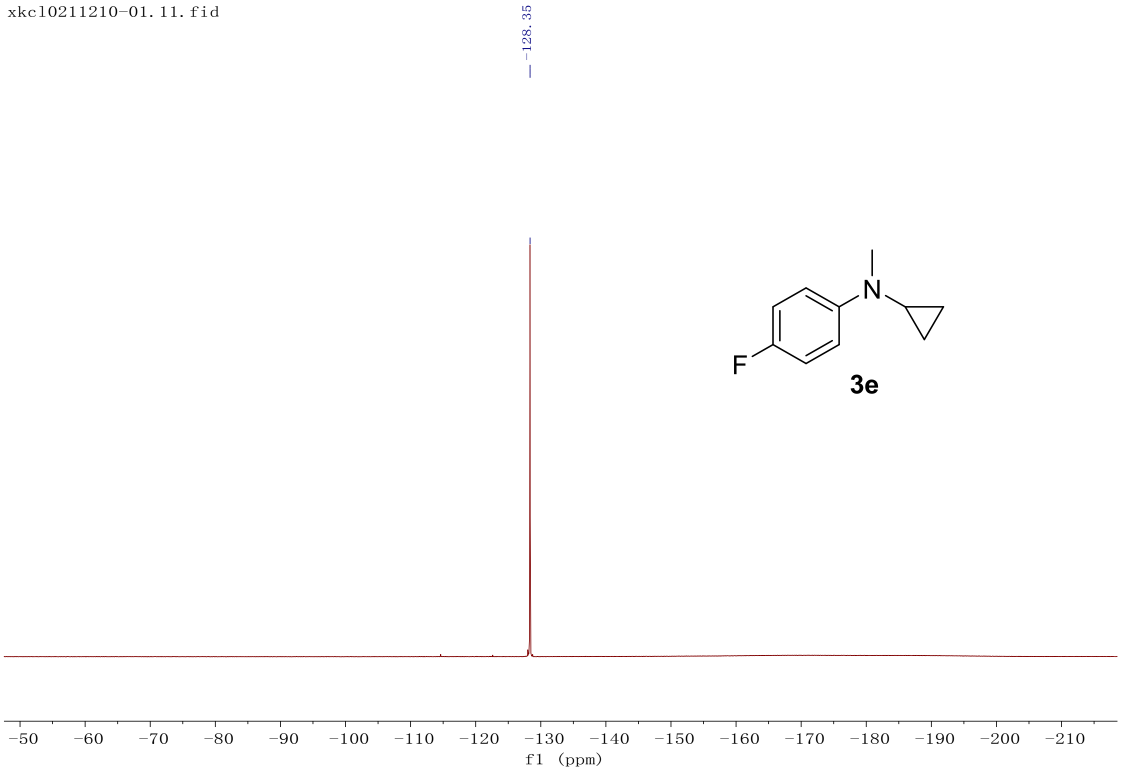

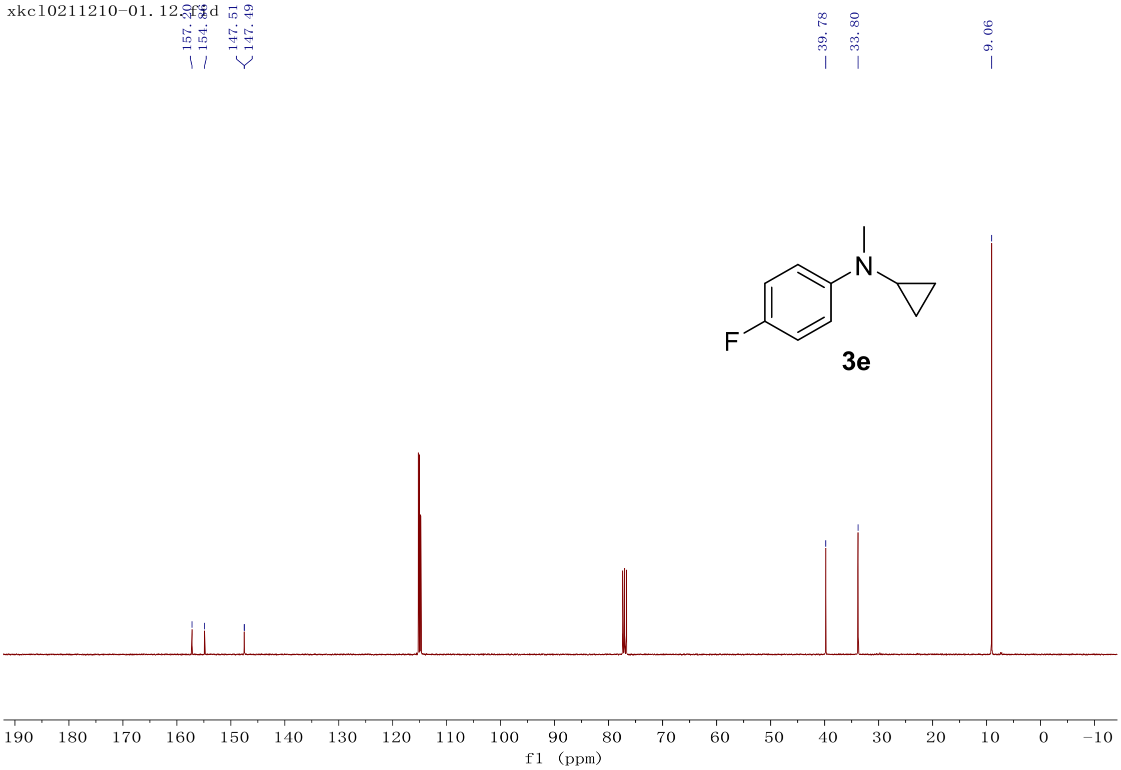
xkc10211210-01.12.fid
157.20
154.86
147.51
147.49
39.78
33.80
9.06
F
N
3e
190 180 170 160 150 140 130 120 110 100 90 80 70 60 50 40 30 20 10 0 −10
f1 (ppm)

NMR spectra of 3f

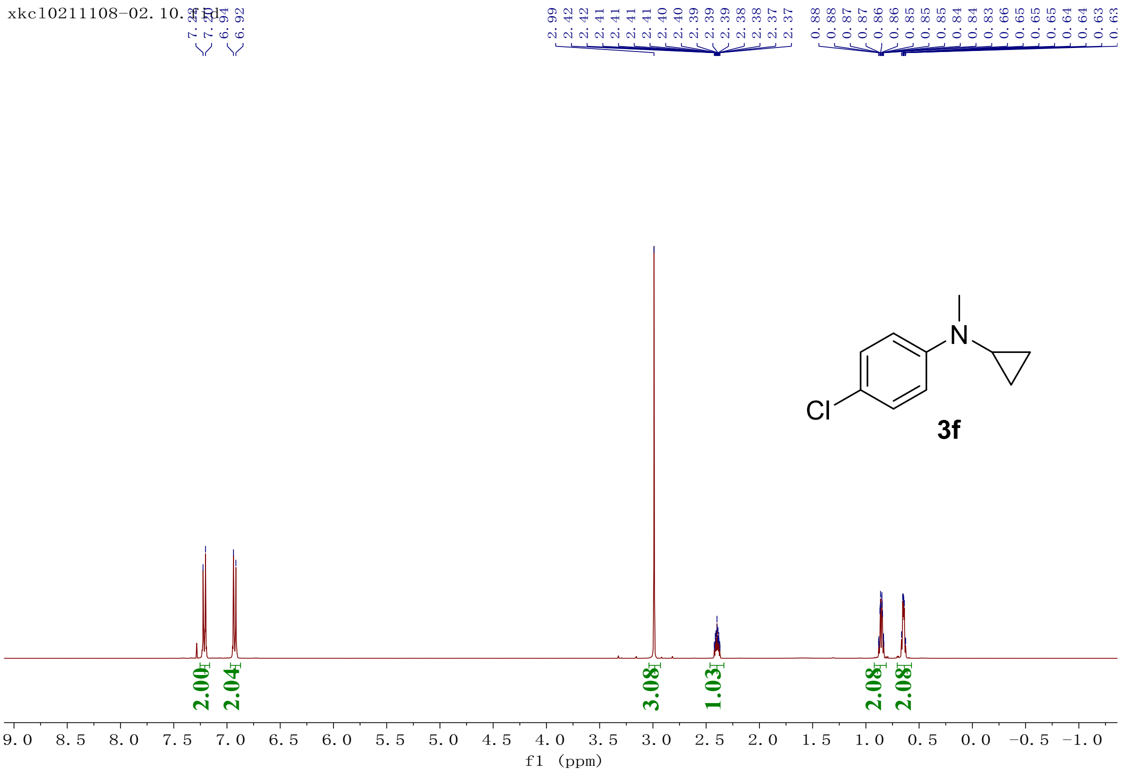

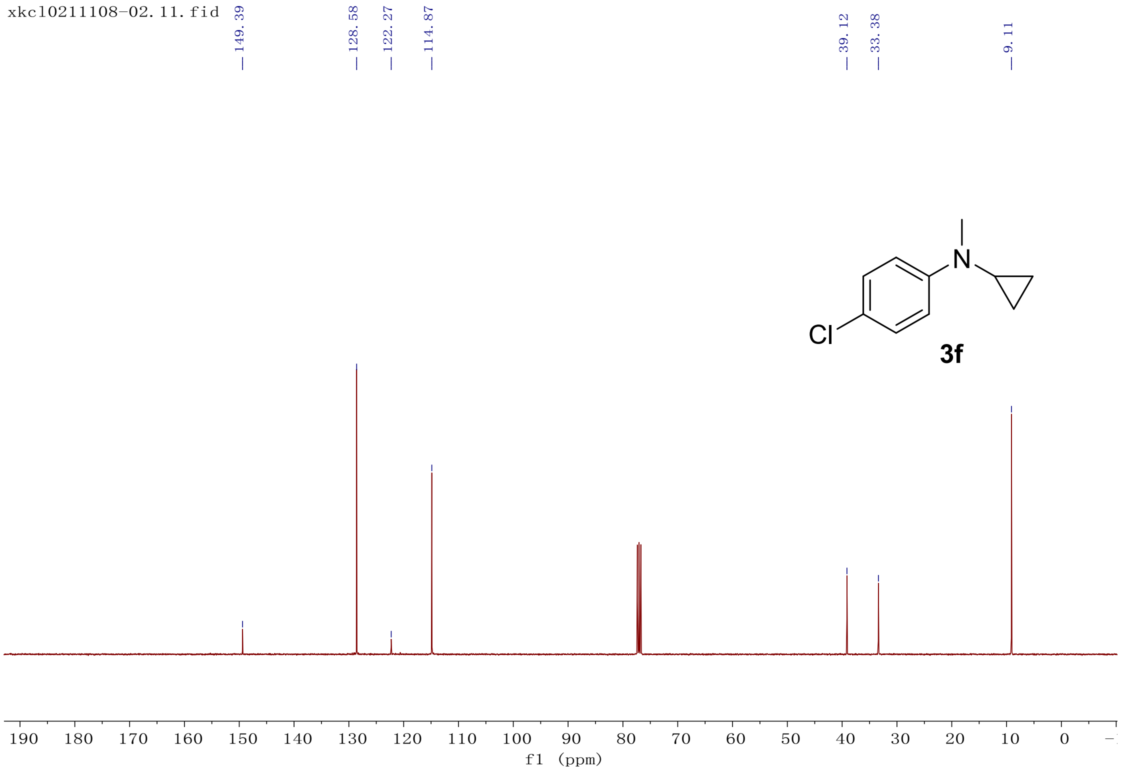

NMR spectra of 3g

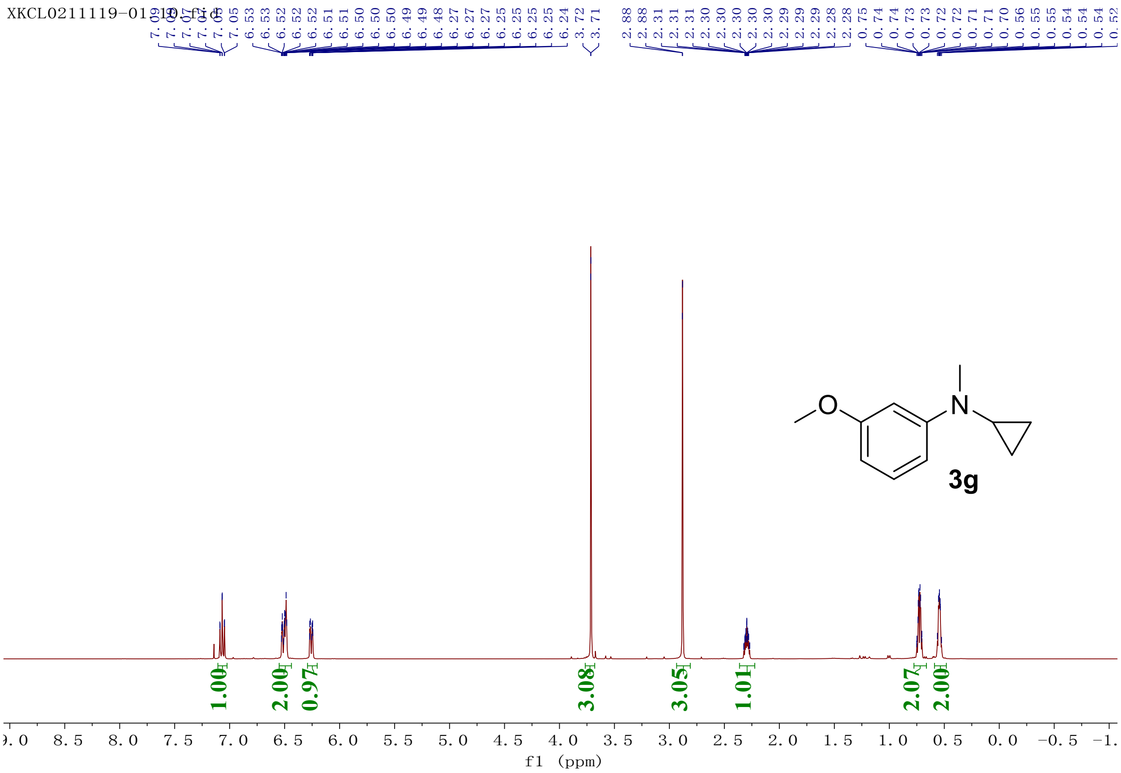

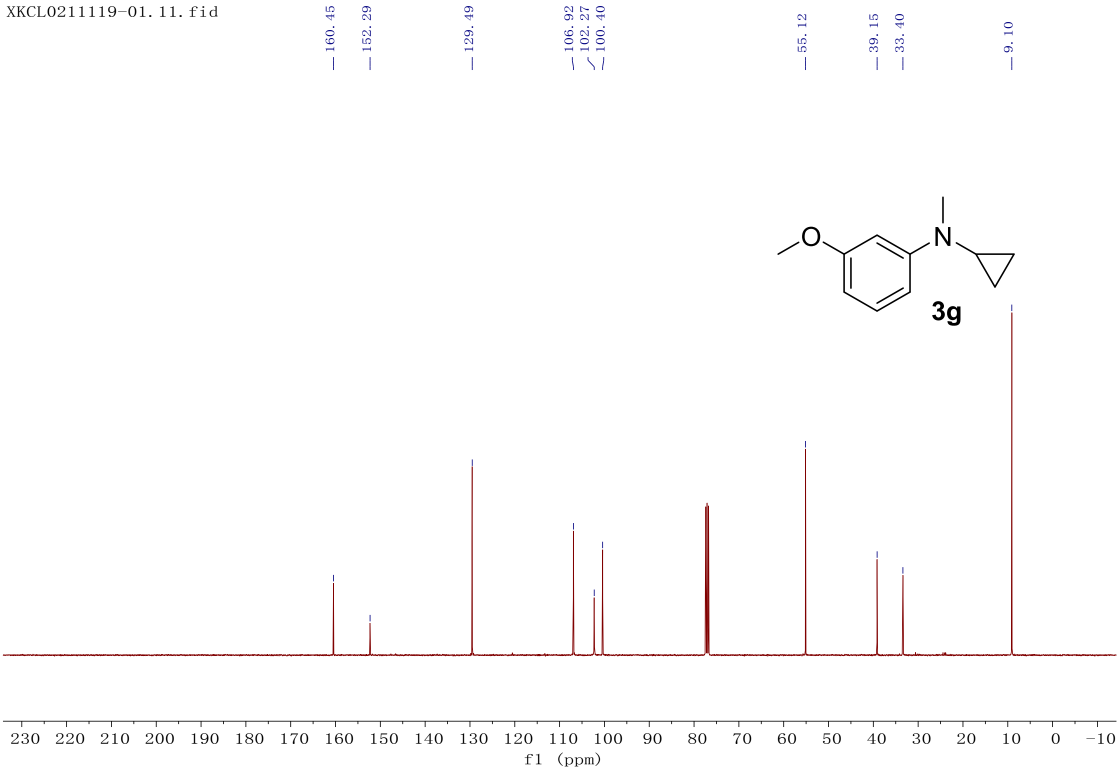

NMR spectra of 3h

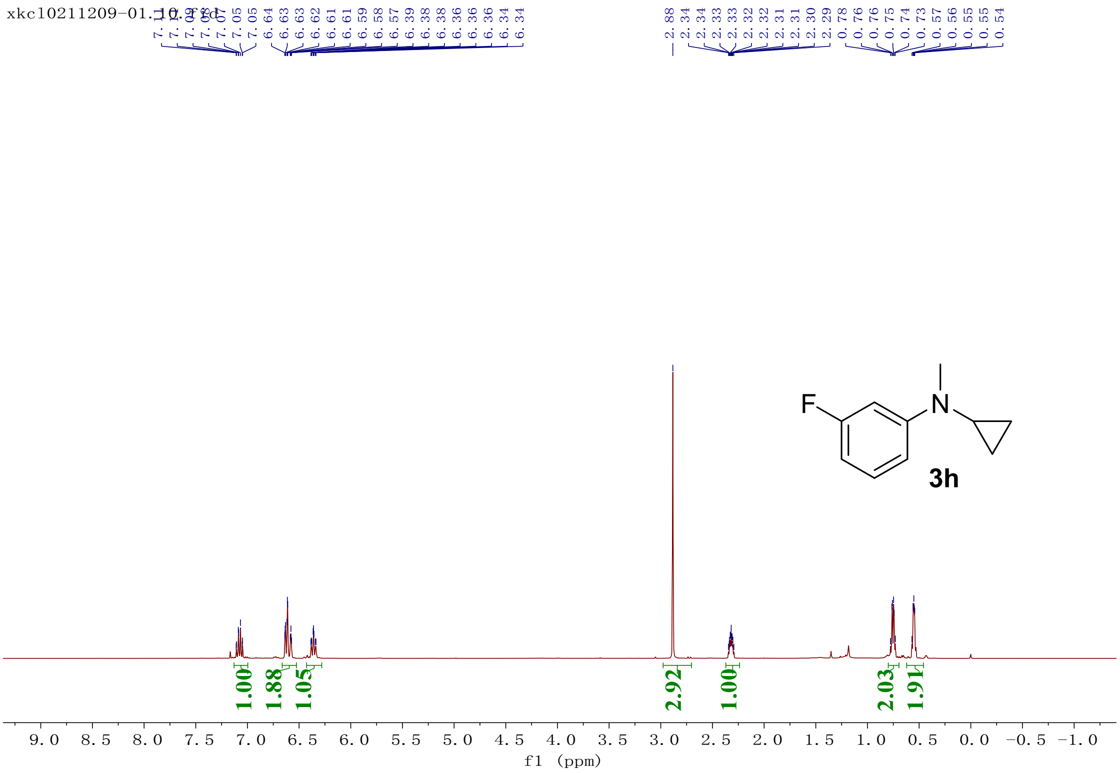

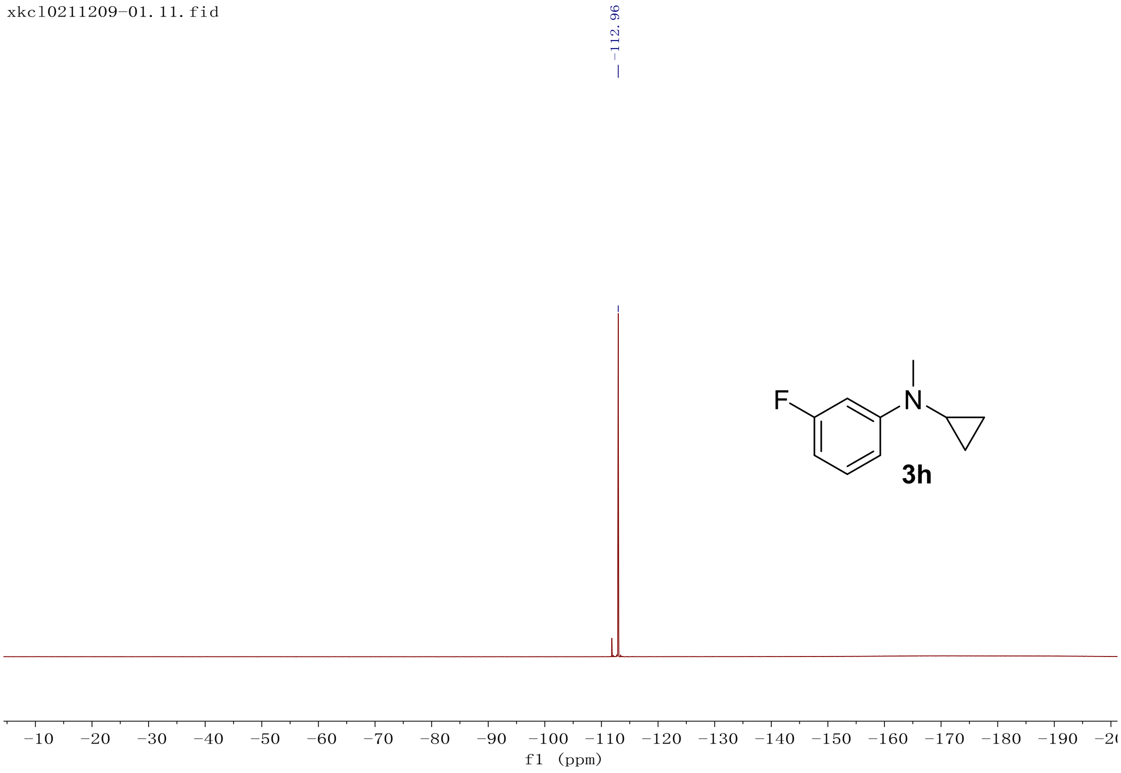

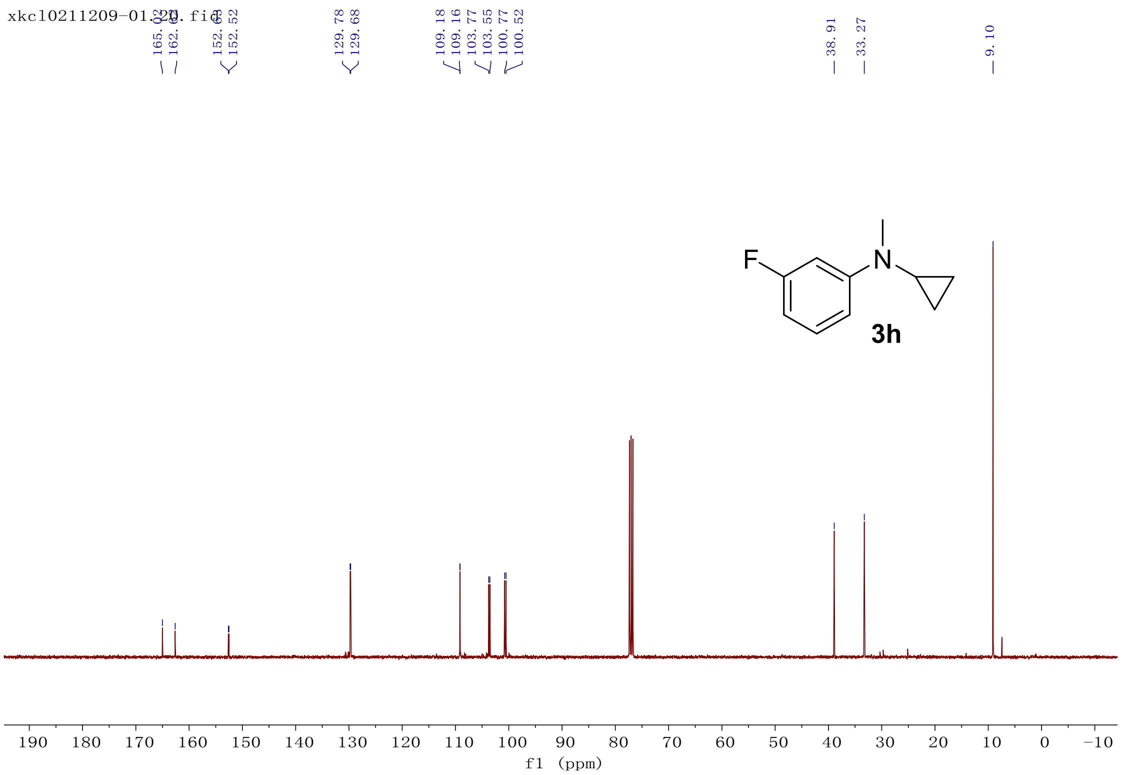
152.52
129.78
129.68
109.18
109.16
103.77
103.55
100.77
100.52
38.91
33.27
9.10
F
N
3h
190 180 170 160 150 140 130 120 110 100 90 80 70 60 50 40 30 20 10 0 −10
f1 (ppm)

NMR spectra of 3i

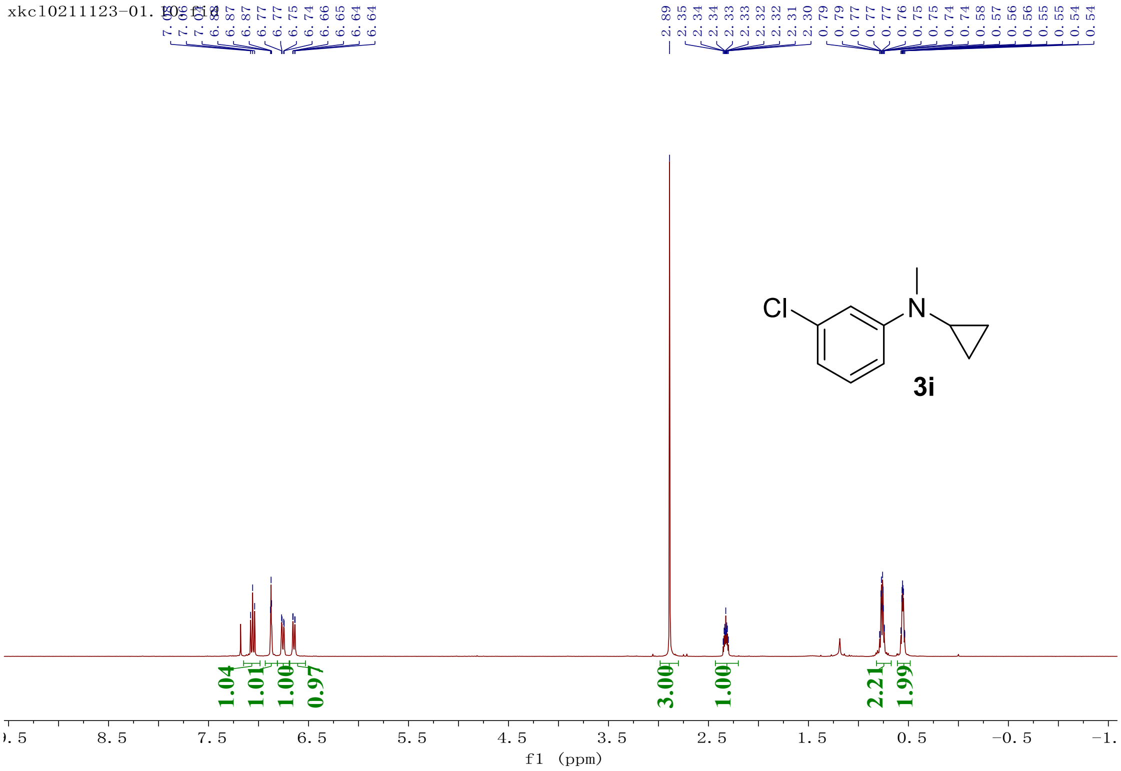

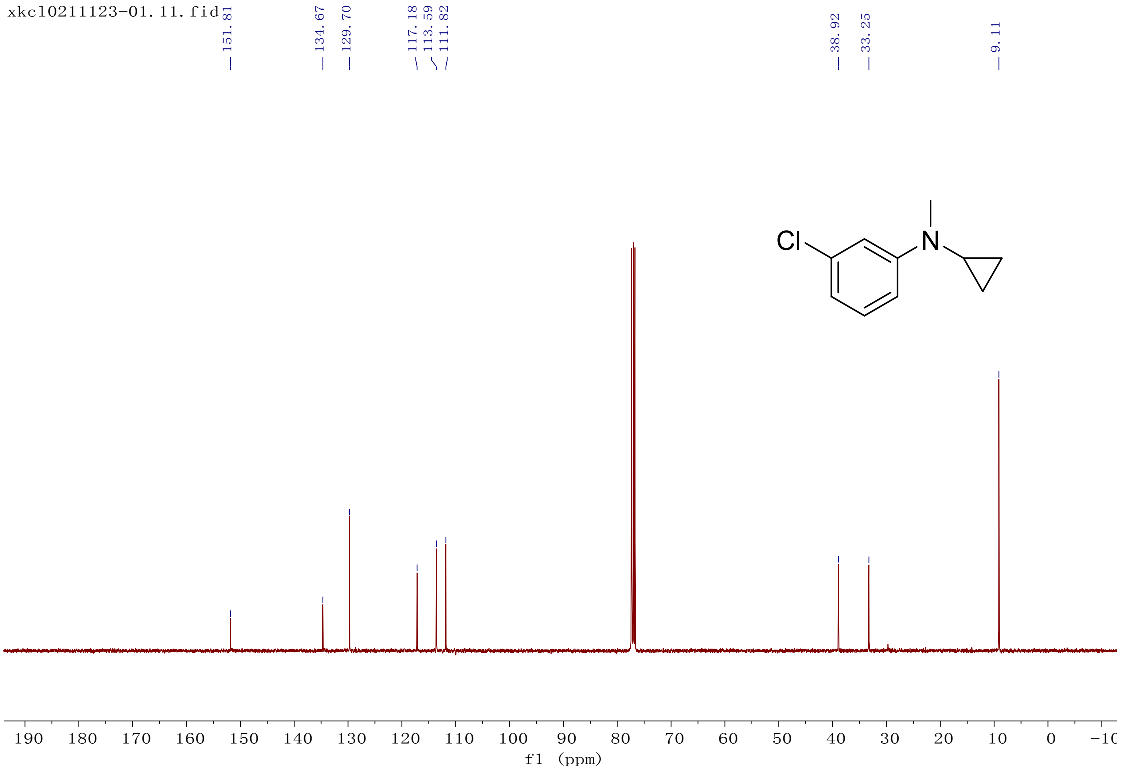

NMR spectra of 3j

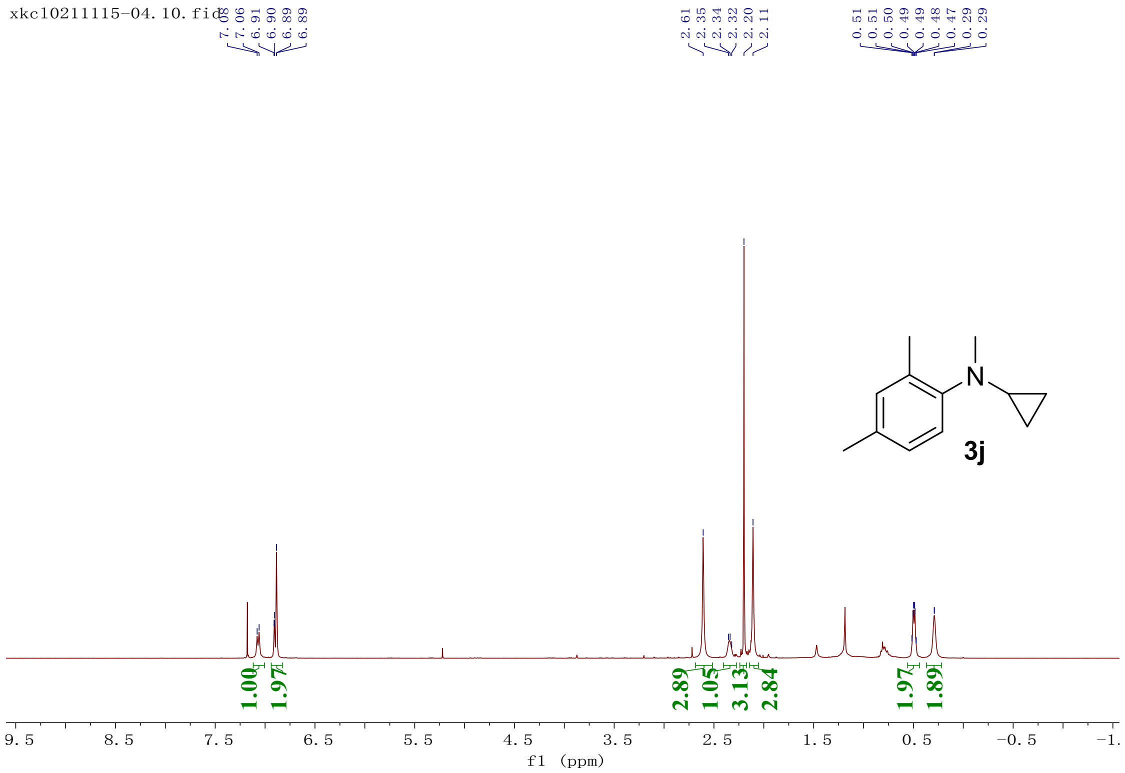

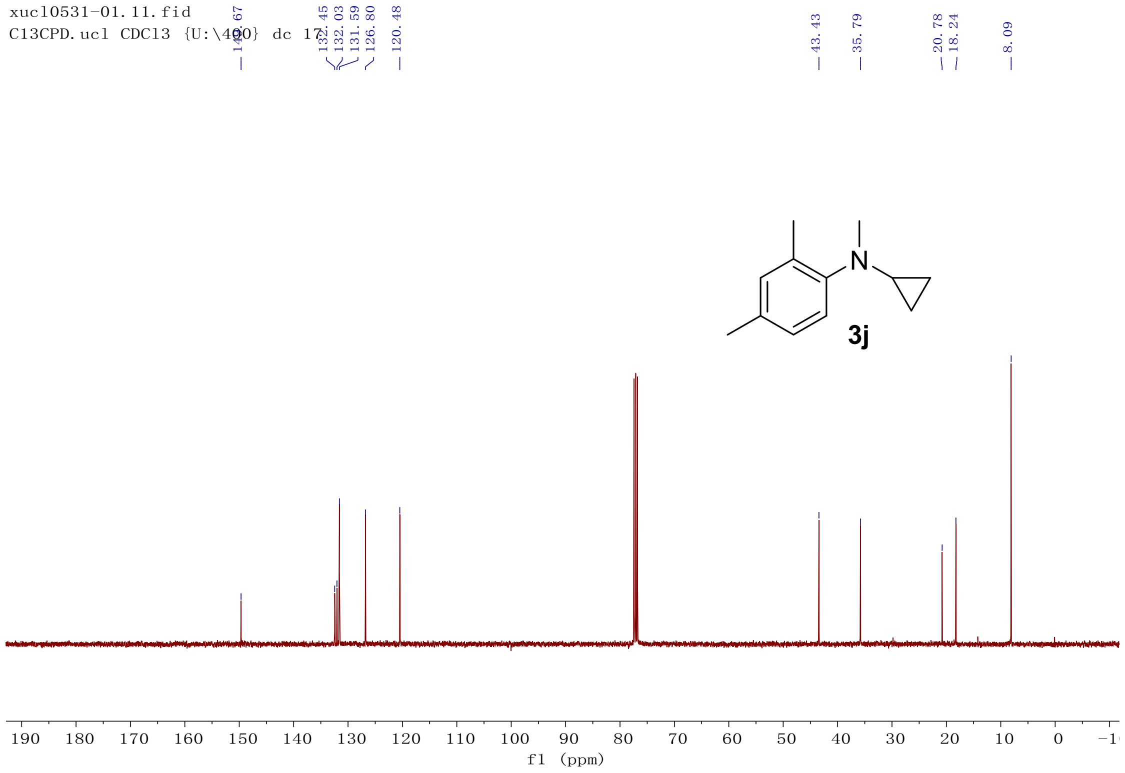

NMR spectra of 3k

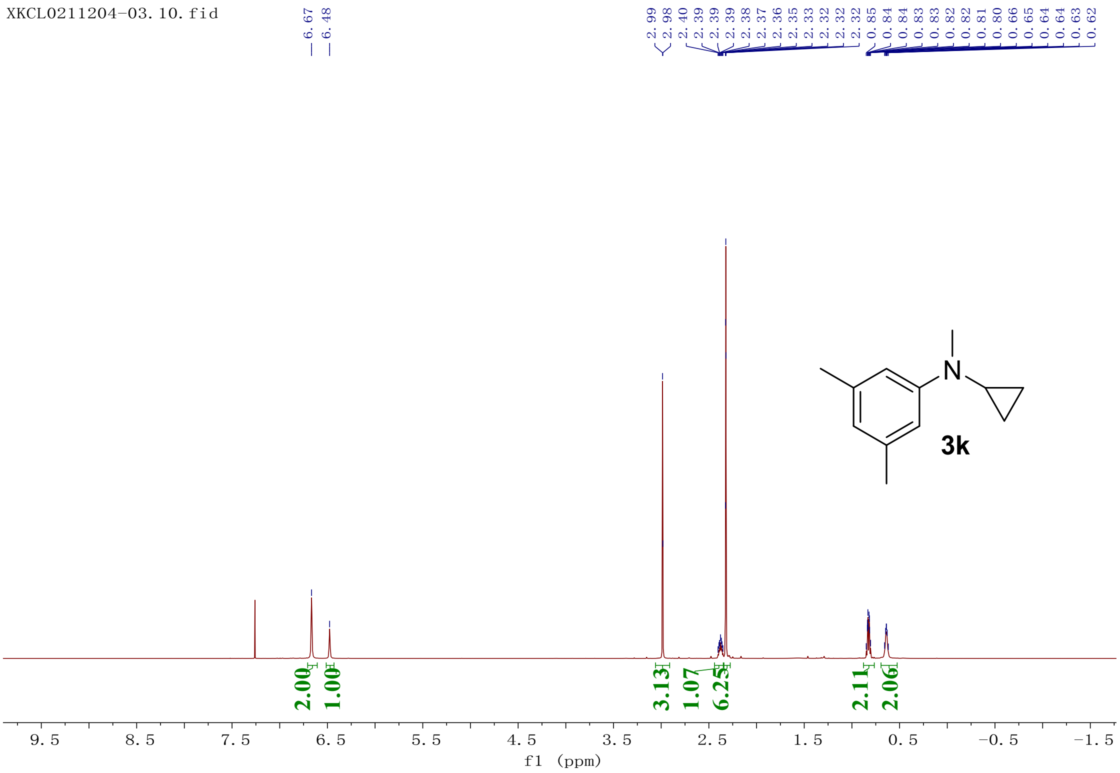

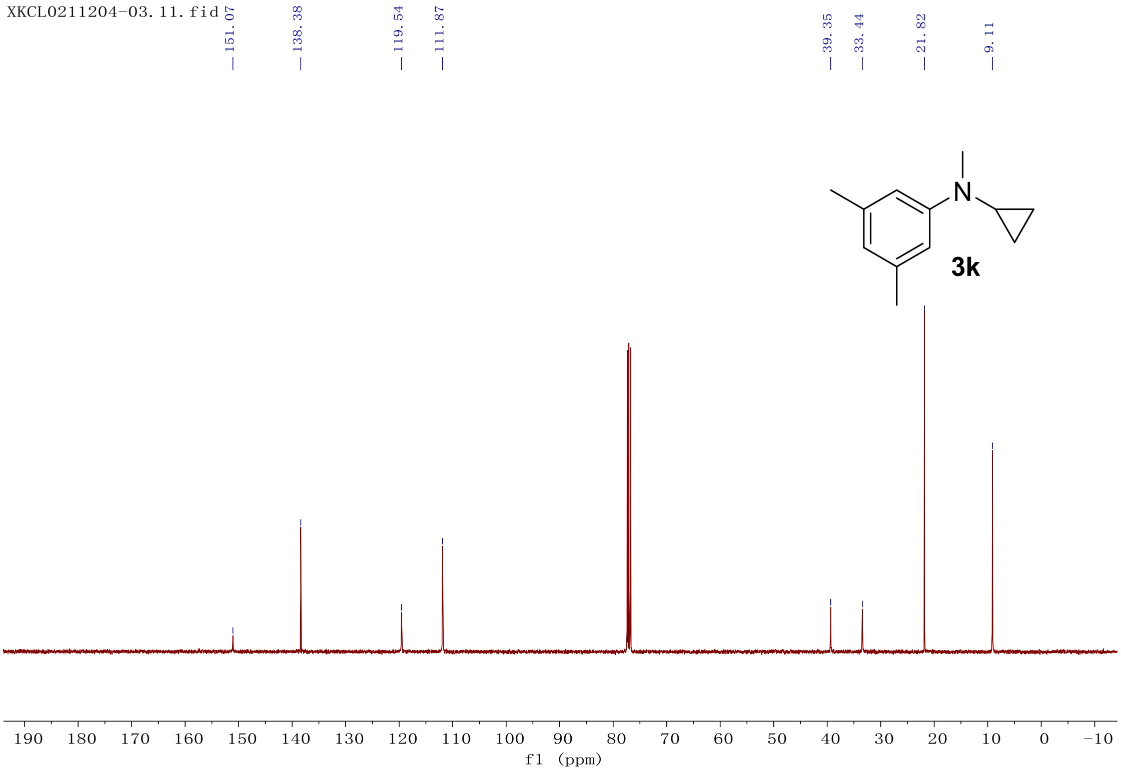

NMR spectra of 3l

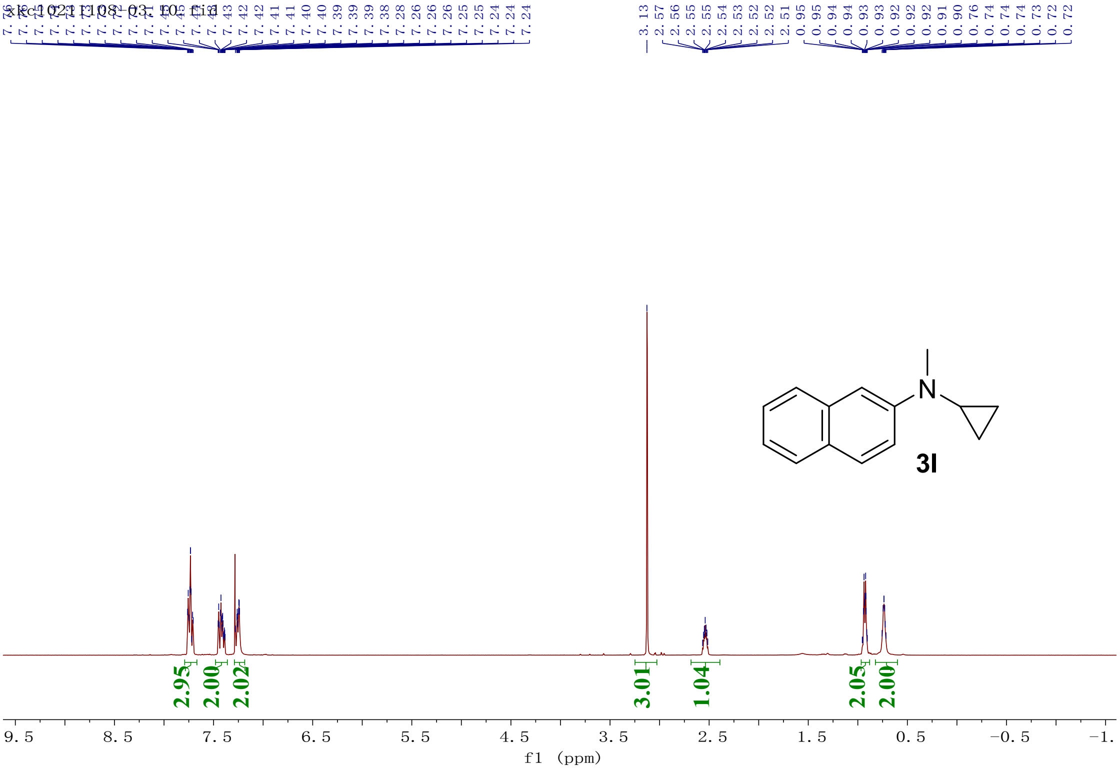

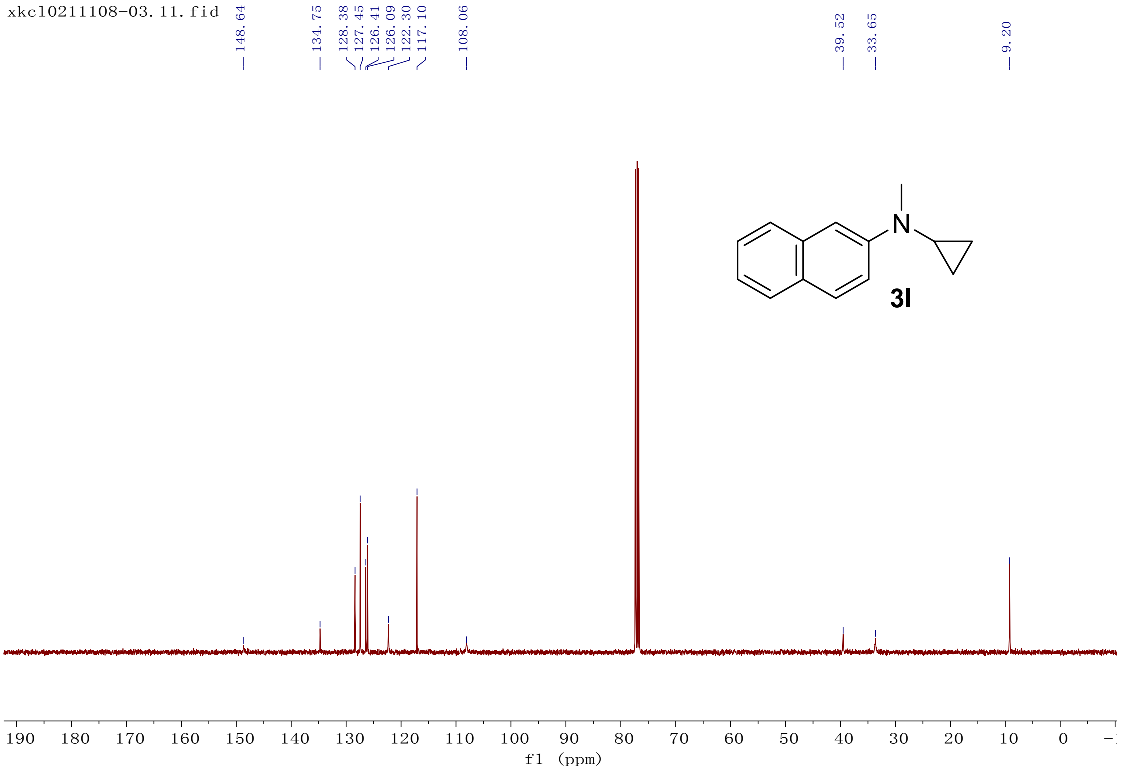

NMR spectra of 3m

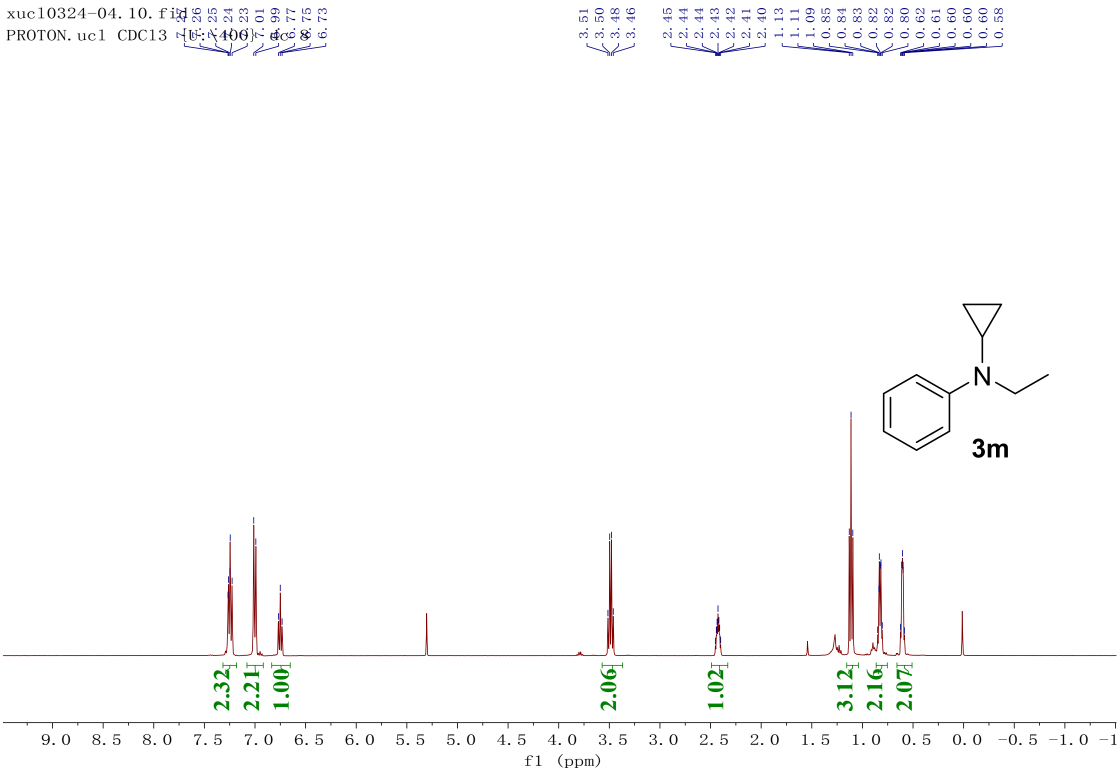

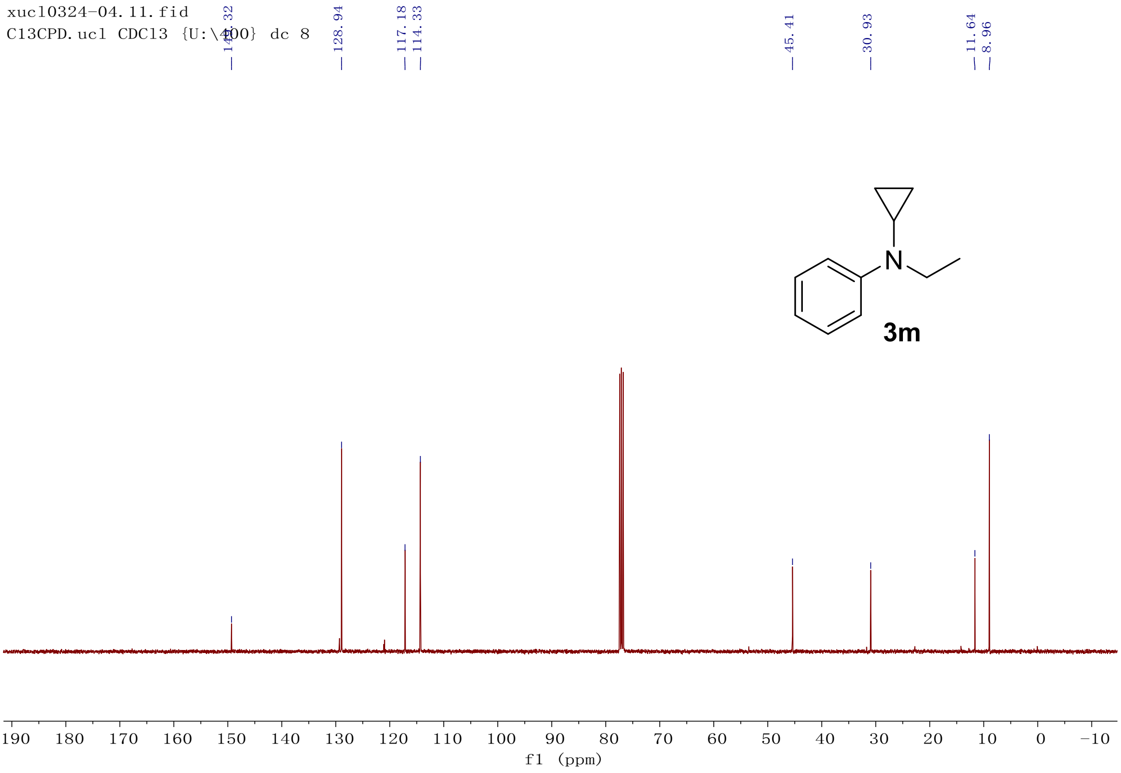

NMR spectra of 5a

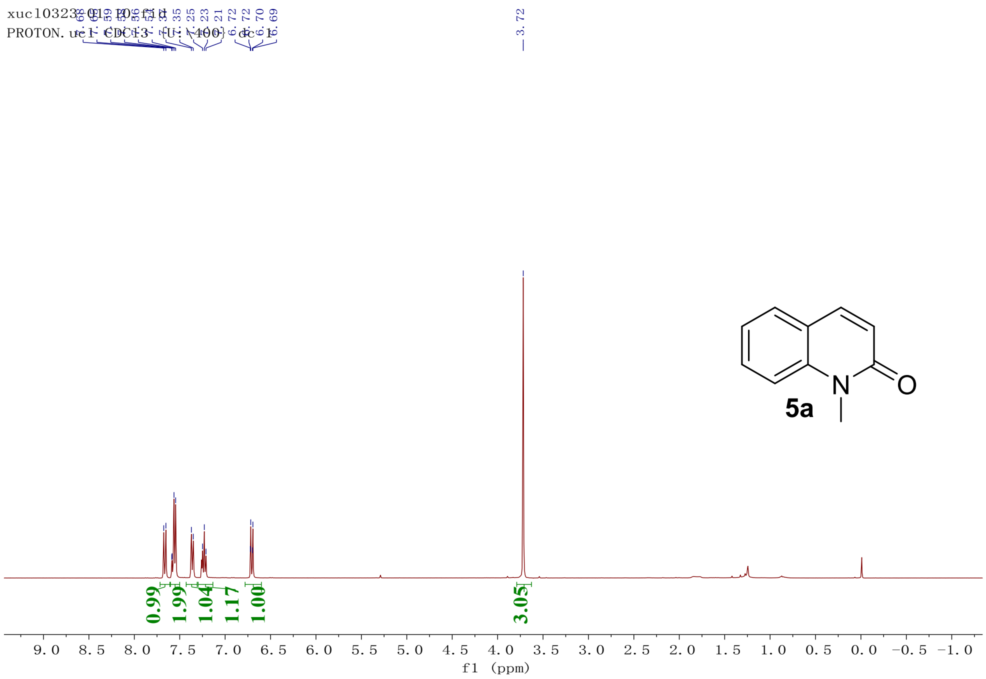

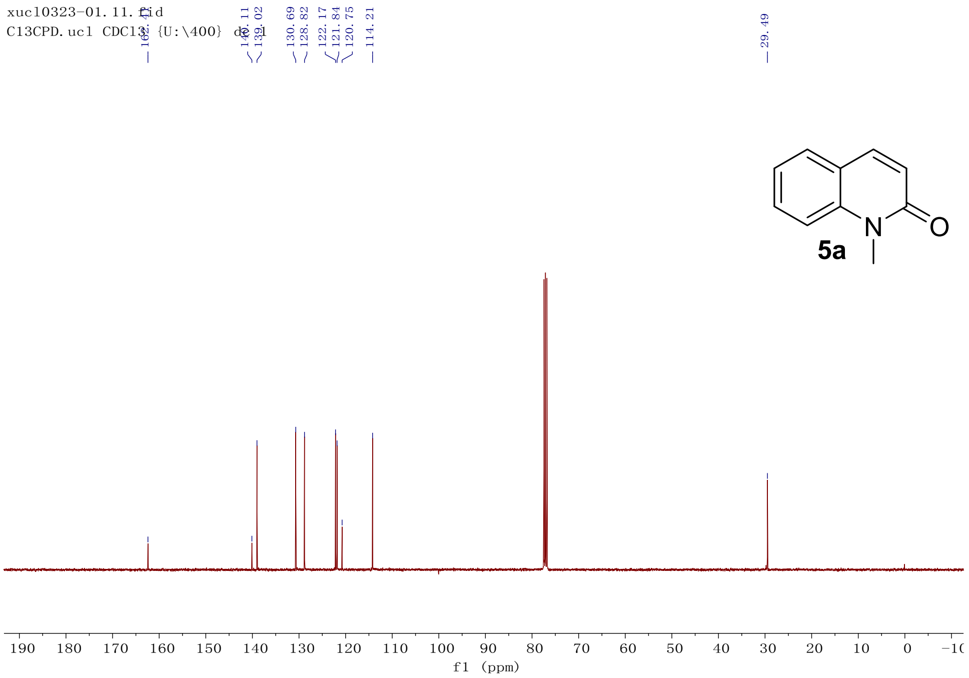

NMR spectra of 5e

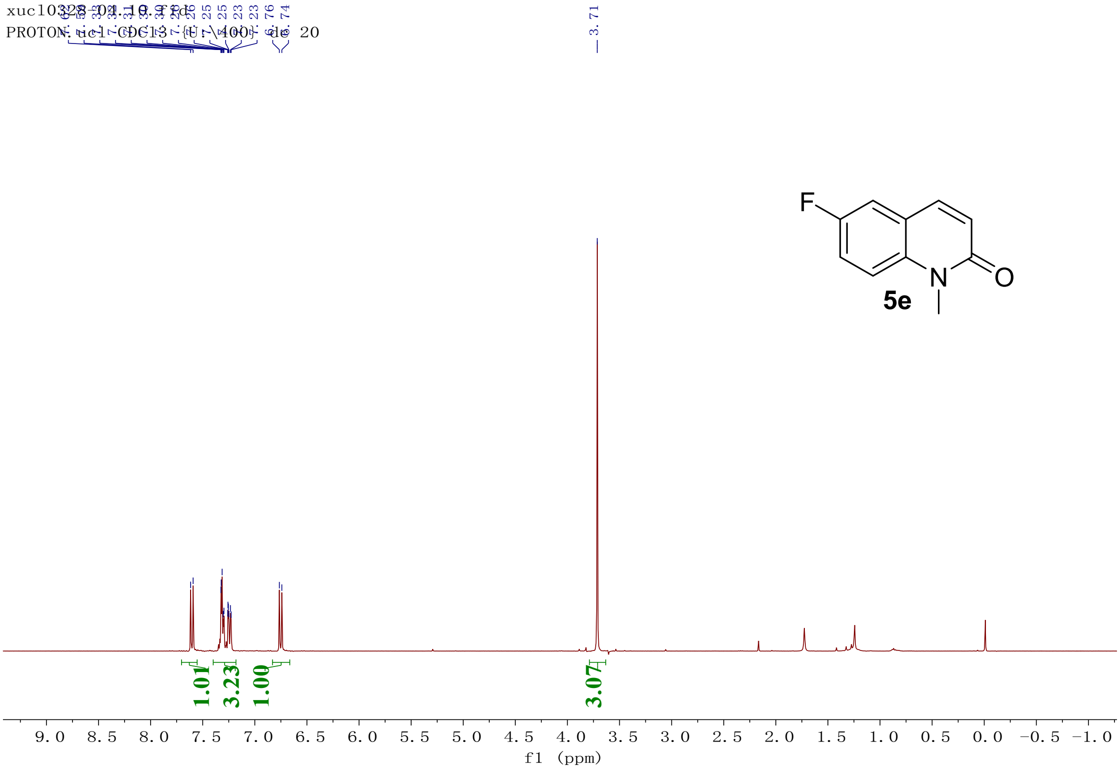

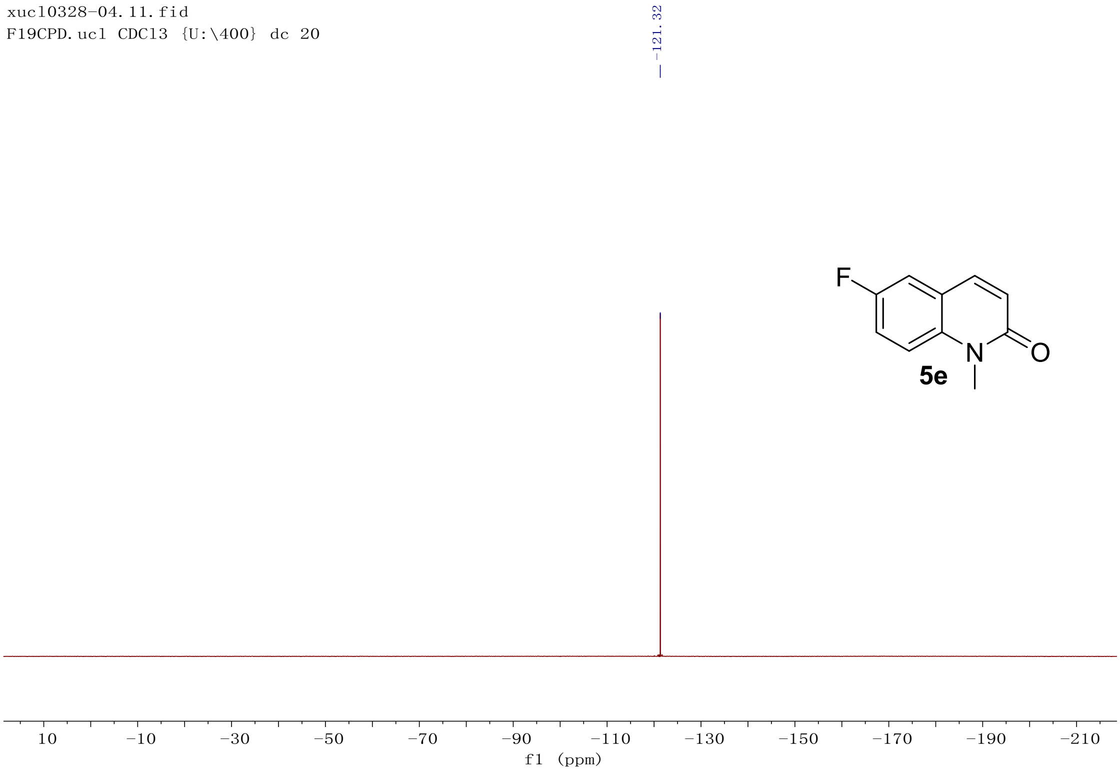

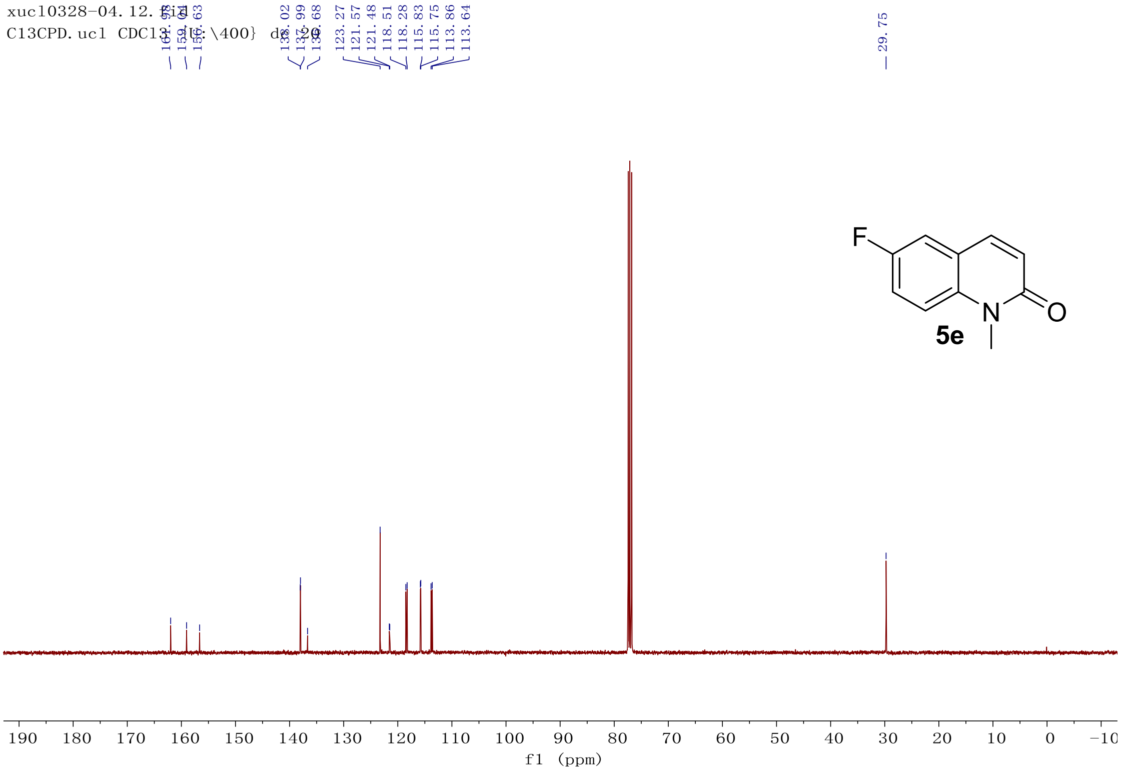
xuc10328-04.12.fid
C13CPD.ucl CDCl3 {U:\400} dc 20
16[illegible].98
159.04
156.63
138.02
137.99
136.68
123.27
121.57
121.48
118.51
118.28
115.83
115.75
113.86
113.64
29.75
F
N
O
5e
190 180 170 160 150 140 130 120 110 100 90 80 70 60 50 40 30 20 10 0 −10
f1 (ppm)

NMR spectra of 5k

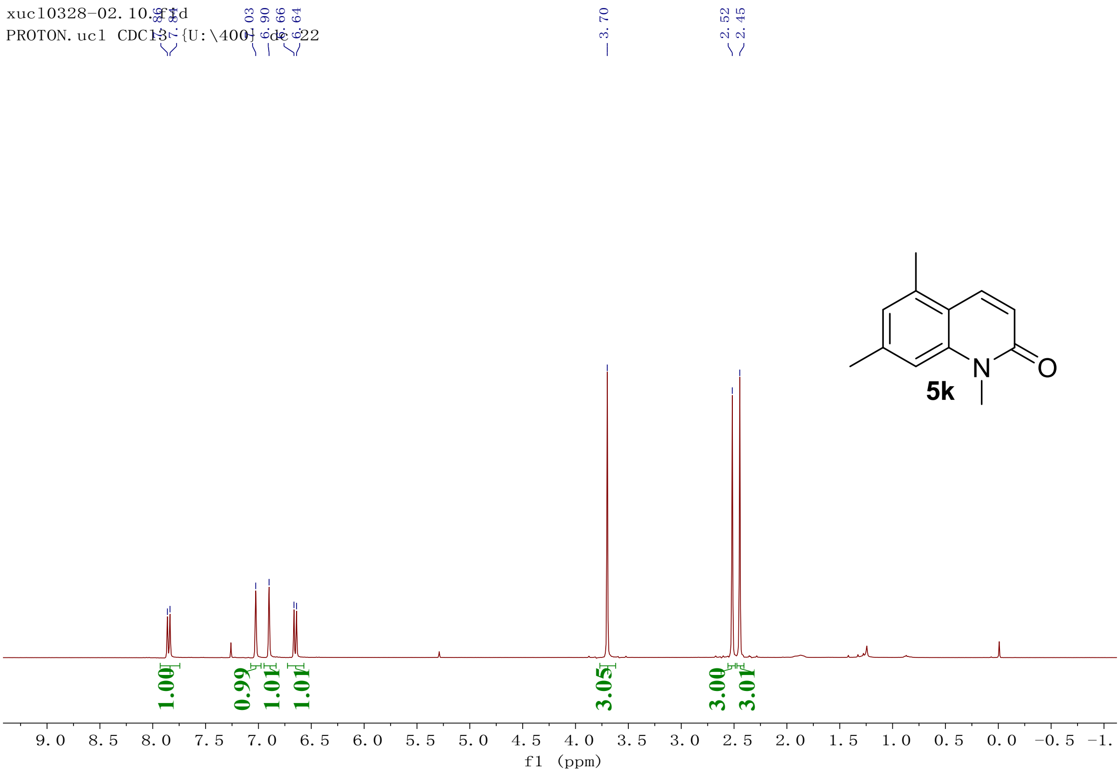

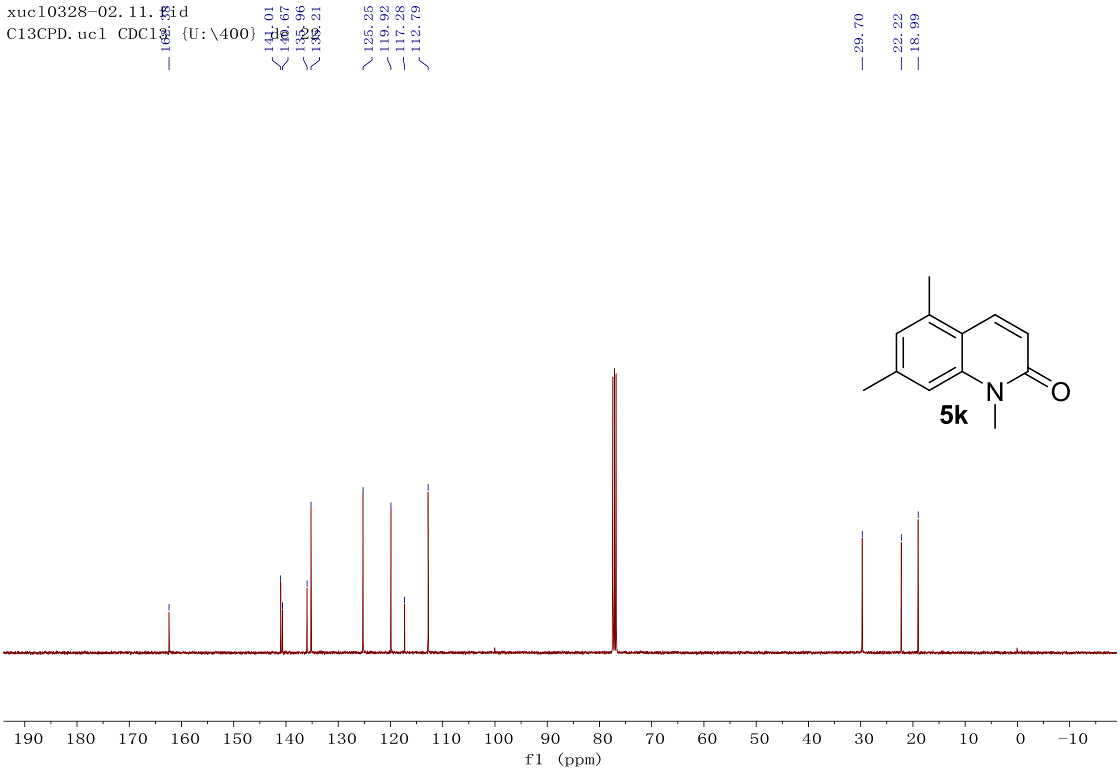

NMR spectra of 5j

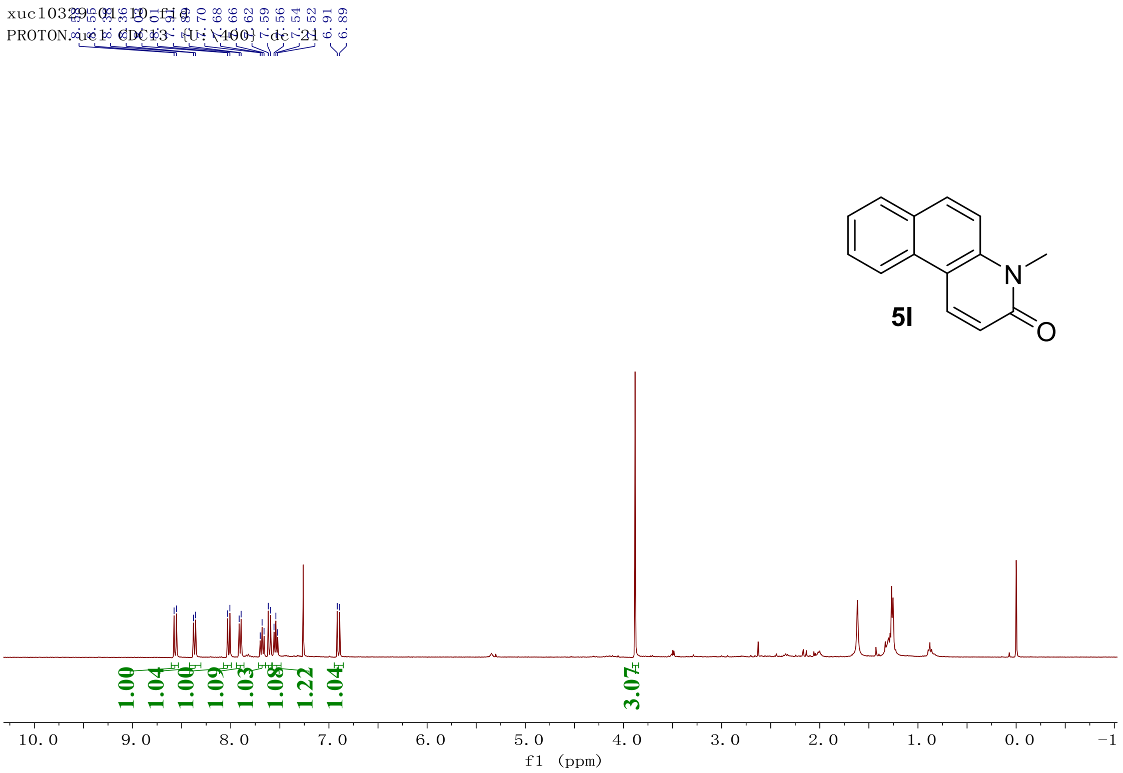

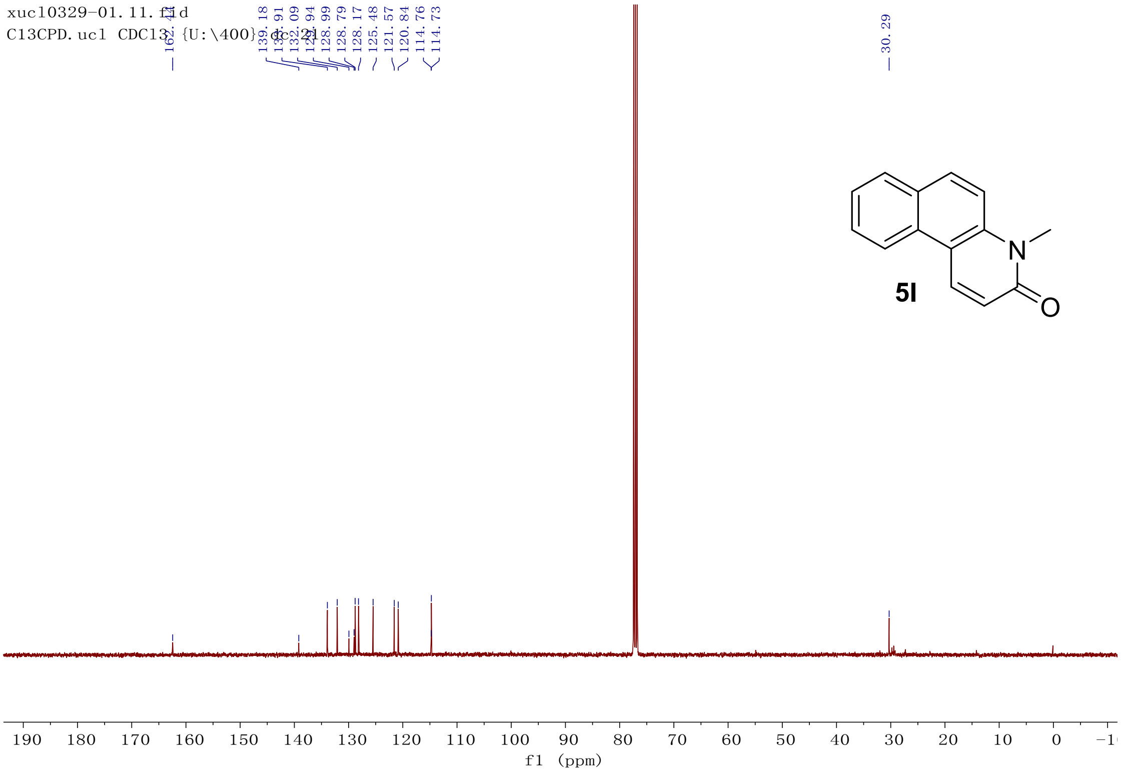

NMR spectra of 5m

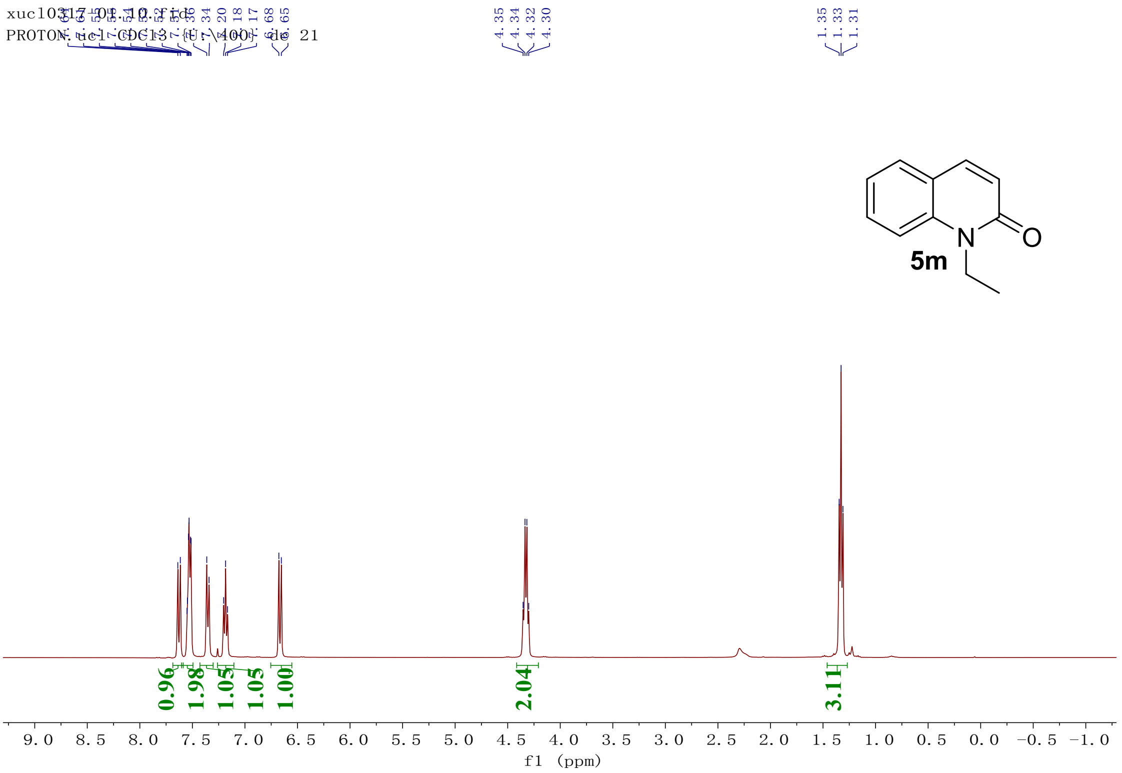

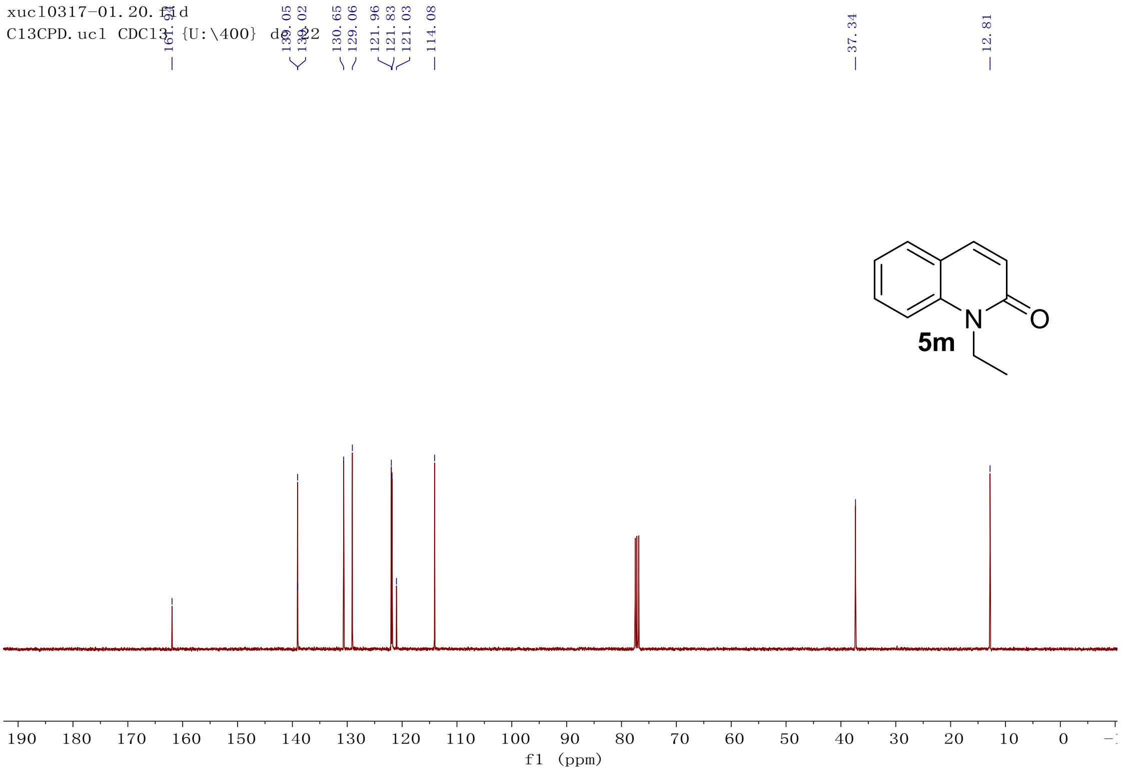

Supplement: Supplementary file 1 — cs2c05902_si_001.pdf [file cs2c05902_si_001.pdf]
